# Supplementary material for: Circular RNAs in Hedgehog Signaling Activation and Hedgehog-Mediated Medulloblastoma Tumors
Source: Cancers (Basel). 2021 Oct 13;13(20):5138. doi: 10.3390/cancers13205138 (PMC8533754; doi:10.3390/cancers13205138)

Sanger sequencing electropherograms for the 29 selected circRNAs (Table 1) and the 2 poly(A) back-spliced RNAs, COL1A2 and TTC21B are shown below.

ARHGAP12

Clip. 1 BQ 20 WL 10 Sequence: ARHGAP

Clipped length:	93	Samples:	12968	Quality: 0 - 9	
Left clip:	11	Bases:	233	10 - 19	
Right clip:	103	Average spacing:	56.0	20 - 29	
Avg. qual. in clip.:	48.0	Average quality >=	10: 102, 20: 11, 30: 72	>= 30	

22.12.2019

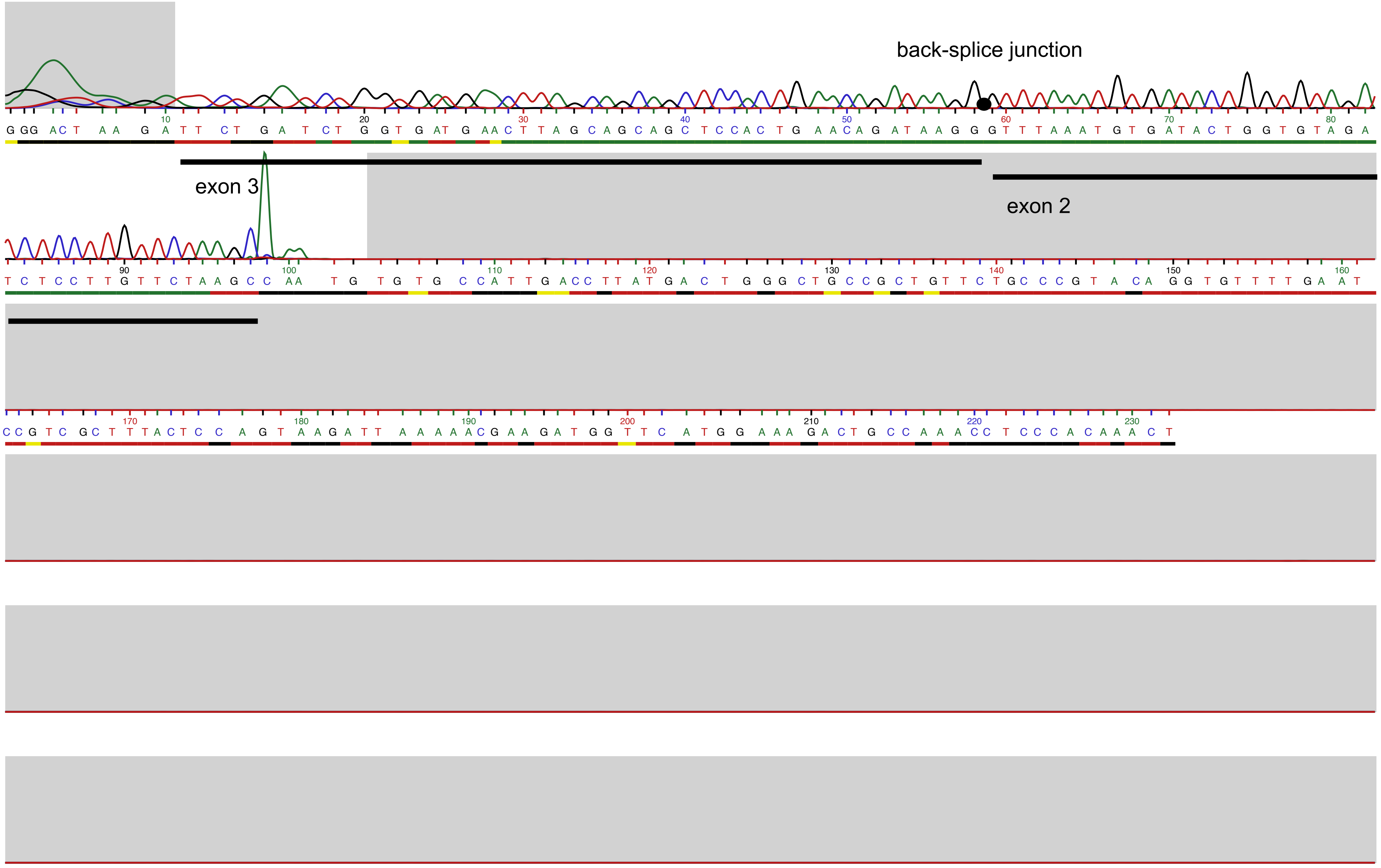

ASXL1

Sequence: ASXL1

Samples:	13007
Bases:	5
Average spacing:	2602.0
Average quality >=	10: 0, 20: 0, 30: 0

Quality:	
0 - 9	black
10 - 19	red
20 - 29	yellow
>= 30	green

13.09.2019

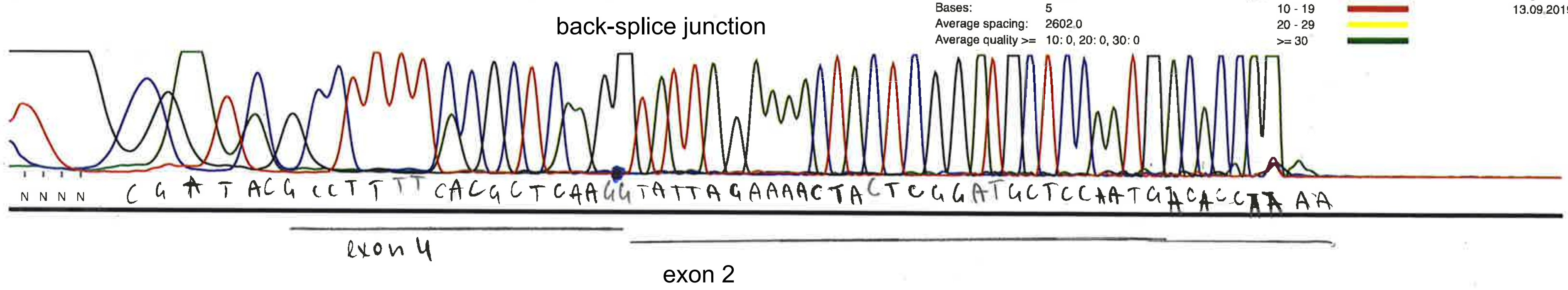

ATXN10

Clip. 1 BQ 20 WL 10 Sequence: ATXN10-1

Clipped length: 113
Left clip: 13
Right clip: 125
Avg. qual. in clip.: 44.34

Samples: 12968
Bases: 126
Average spacing: 103.0
Average quality >= 10: 14, 20: 6, 30: 89

Quality: 0 - 9, 10 - 19, 20 - 29, >= 30

Page: 1 / 3
22.08.2019

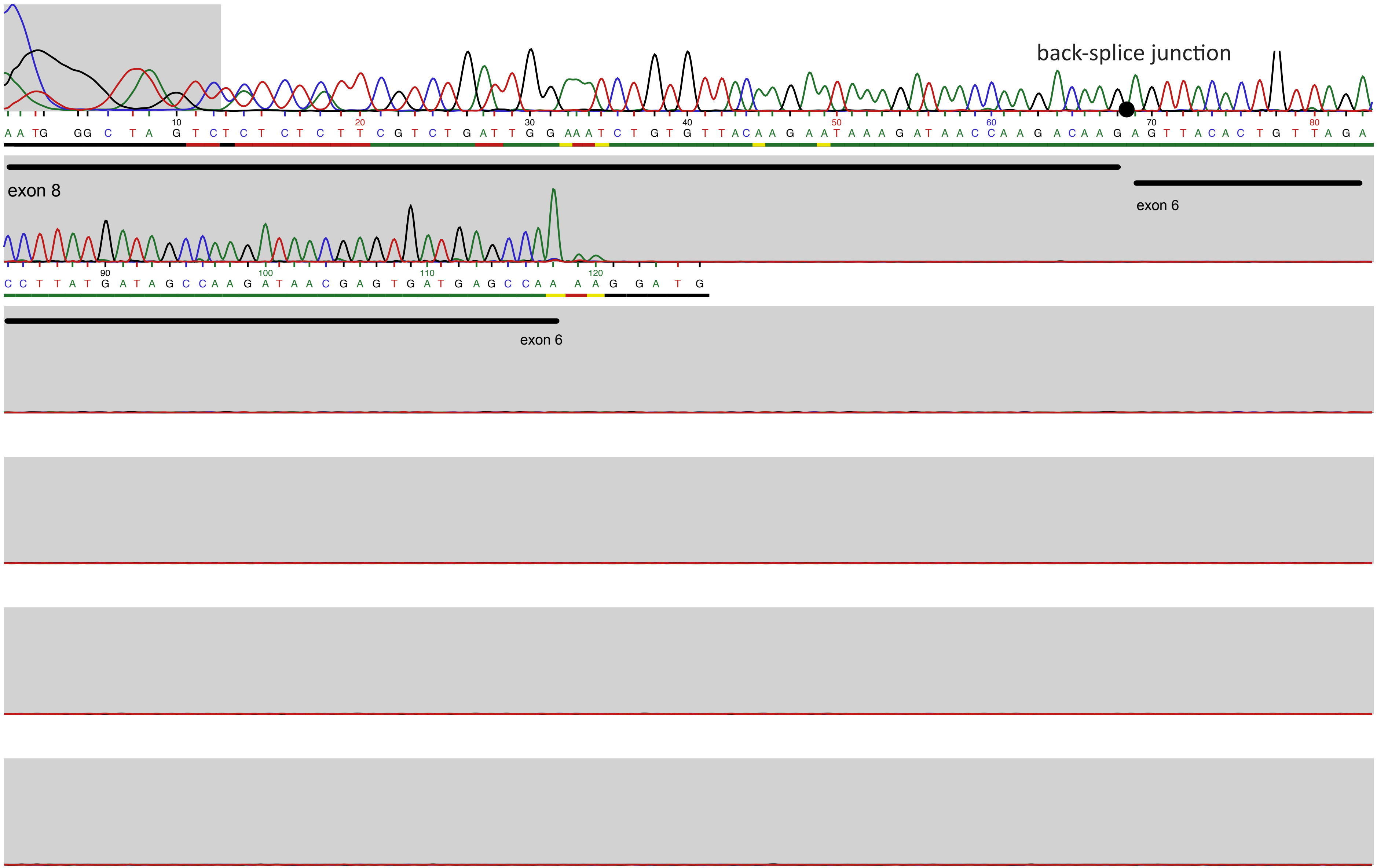

ATXN10-2

Clip. 1 BQ 20 WL 10 Sequence: ATXN10

Clipped length:	113	Samples:	12966	Quality: 0 - 9	
Left clip:	11	Bases:	181	10 - 19	
Right clip:	123	Average spacing:	72.0	20 - 29	
Avg. qual. in clip.:	42.76	Average quality >=	10: 28, 20: 7, 30: 135	>= 30	

02.01.2020

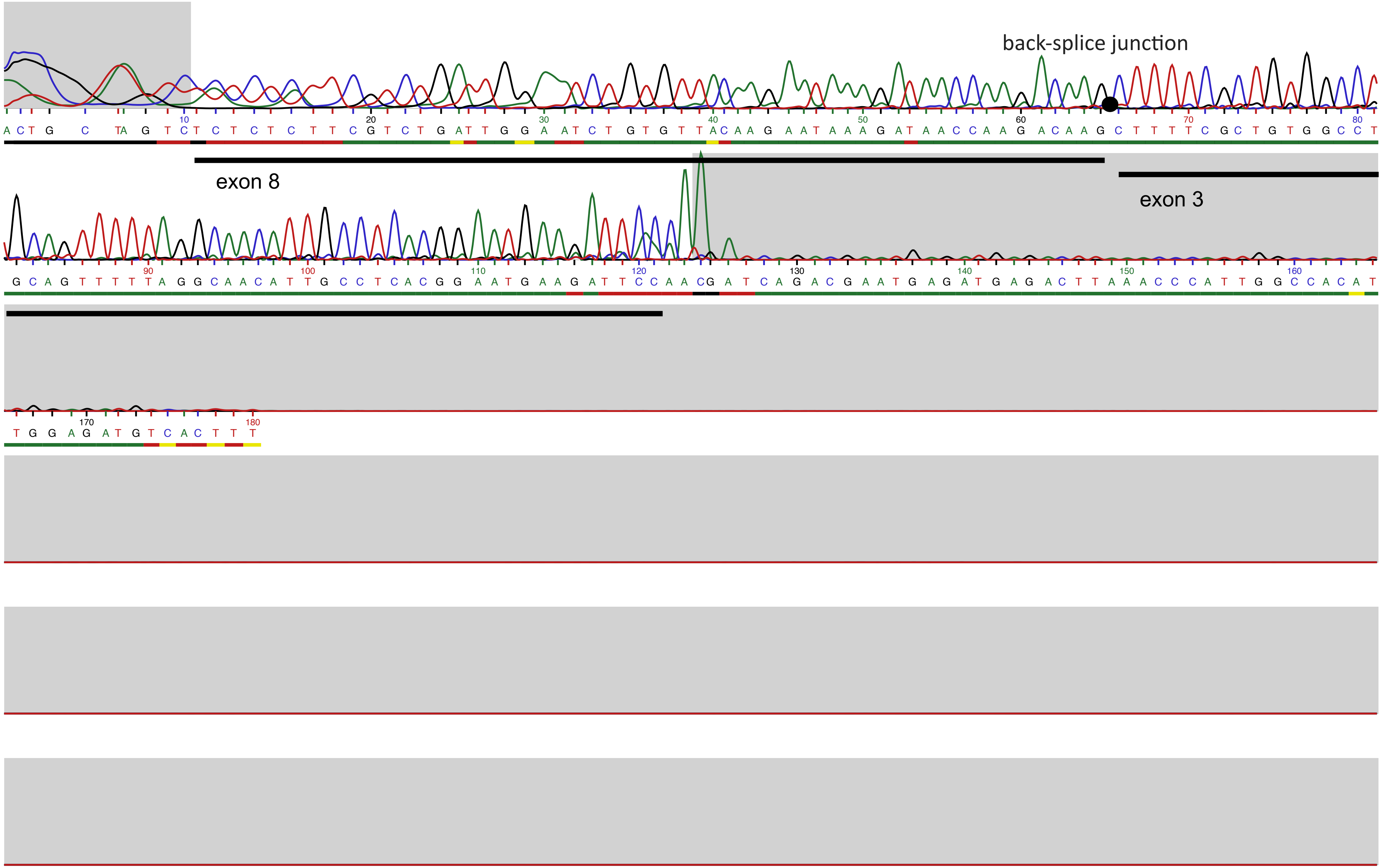

BACH1

Clip. 1 BQ 20 WL 10 Sequence: BACH1

Clipped length:	69	Samples:	12961	Quality: 0 - 9	
Left clip:	13	Bases:	395	10 - 19	
Right clip:	81	Average spacing:	33.0	20 - 29	
Avg. qual. in clip.:	38.85	Average quality >=	10: 239, 20: 32, 30: 52	>= 30	

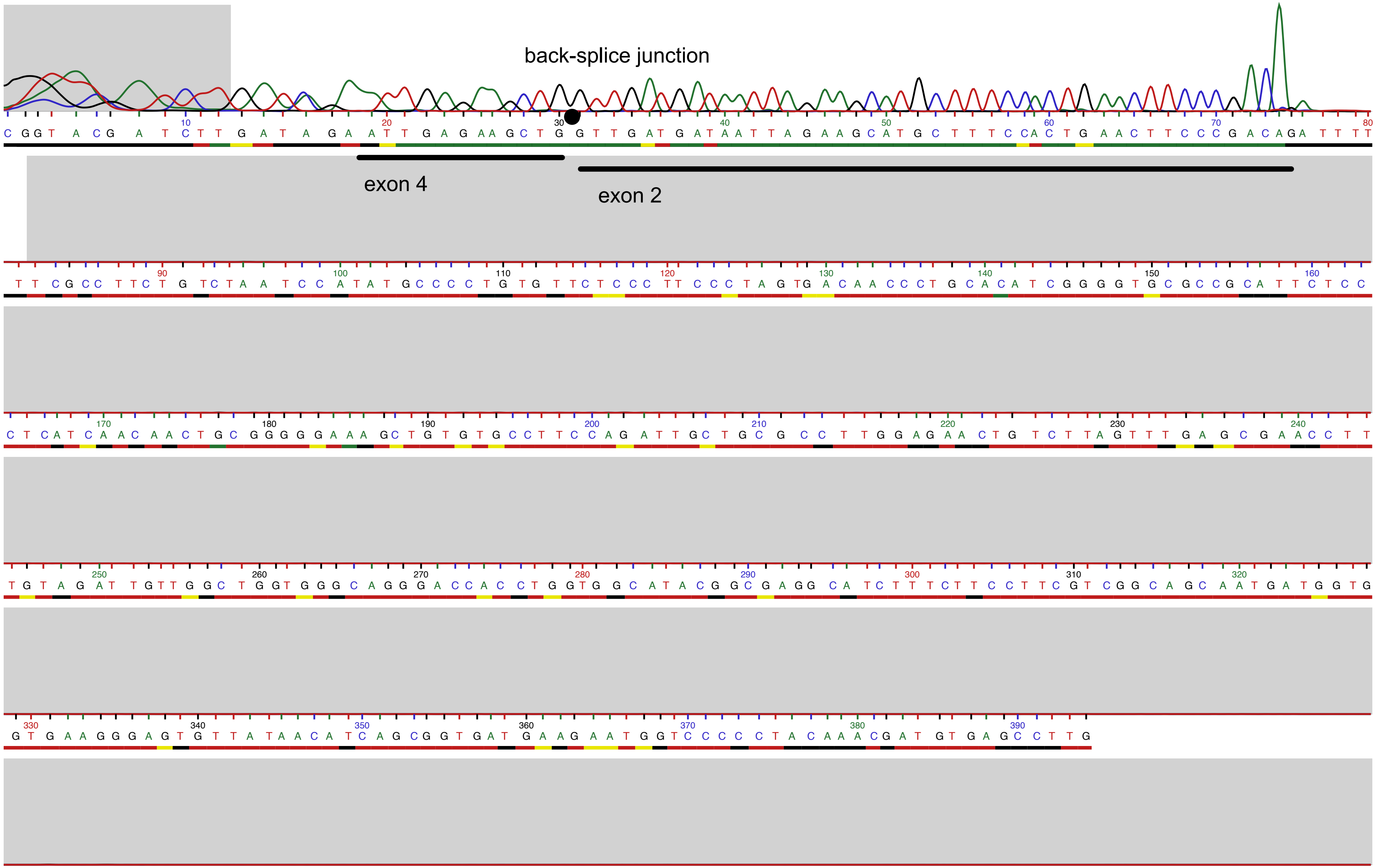

CBFA2T2

Clip. 1 BQ 20 WL 10 Sequence: CBFA2T2

Clipped length:	67	Samples:	12963	Quality:	0 - 9		Page: 1 / 3
Left clip:	13	Bases:	333		10 - 19		22.12.2019
Right clip:	79	Average spacing:	39.0		20 - 29		
Avg. qual. in clip.:	43.35	Average quality >=	10: 163, 20: 42, 30: 51		>= 30		

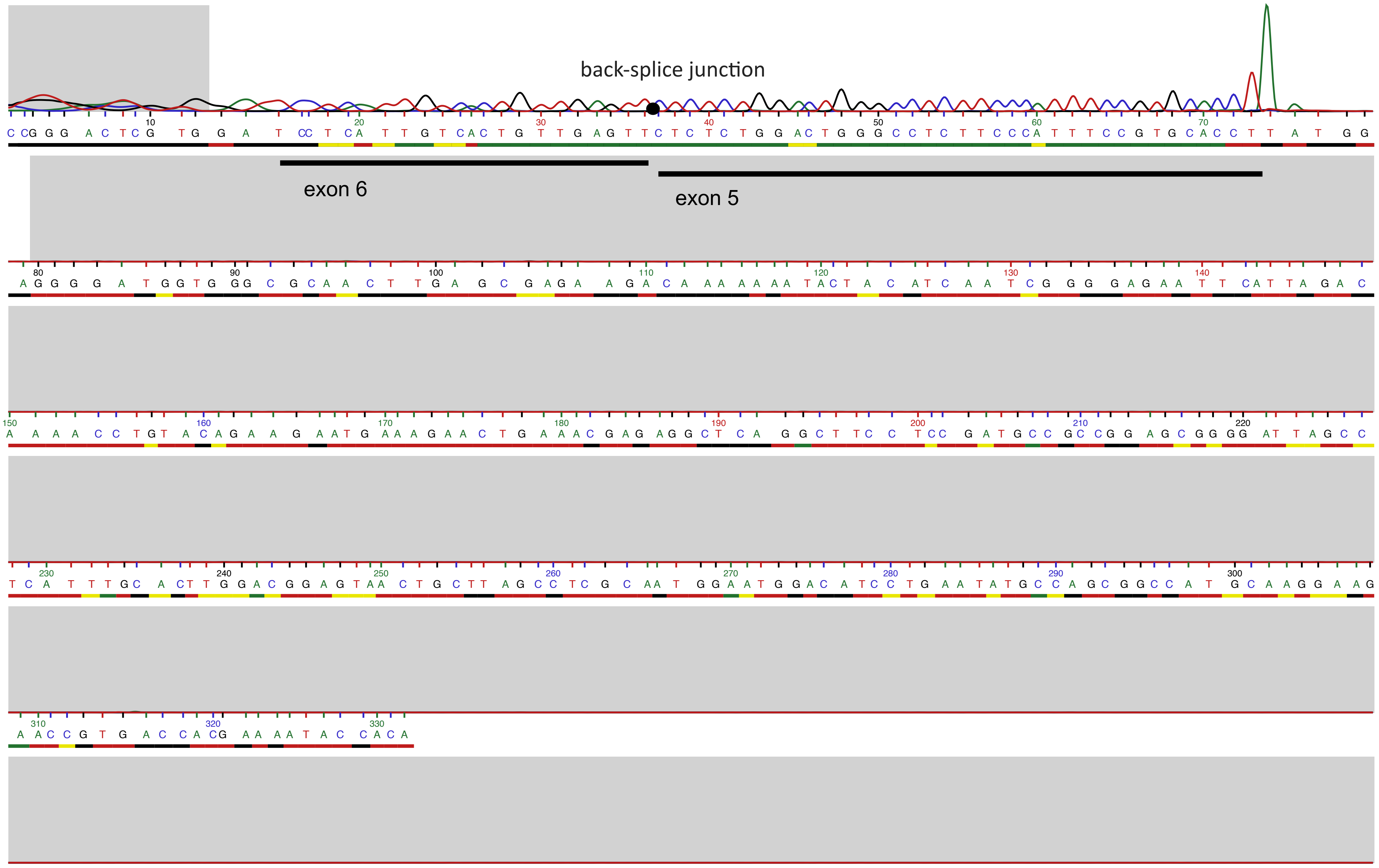

CCDC134

reverse primer

Clip. 1 BQ 20 WL 10 Sequence: CCDC134

Clipped length:	120	Samples:	4350
Left clip:	9	Bases:	128
Right clip:	128	Average spacing:	34.0
Avg. qual. in clip.:	43.83	Average quality >=	10: 7, 20: 15, 30: 98

Quality: 0 - 9, 10 - 19, 20 - 29, >= 30

22.02.2020

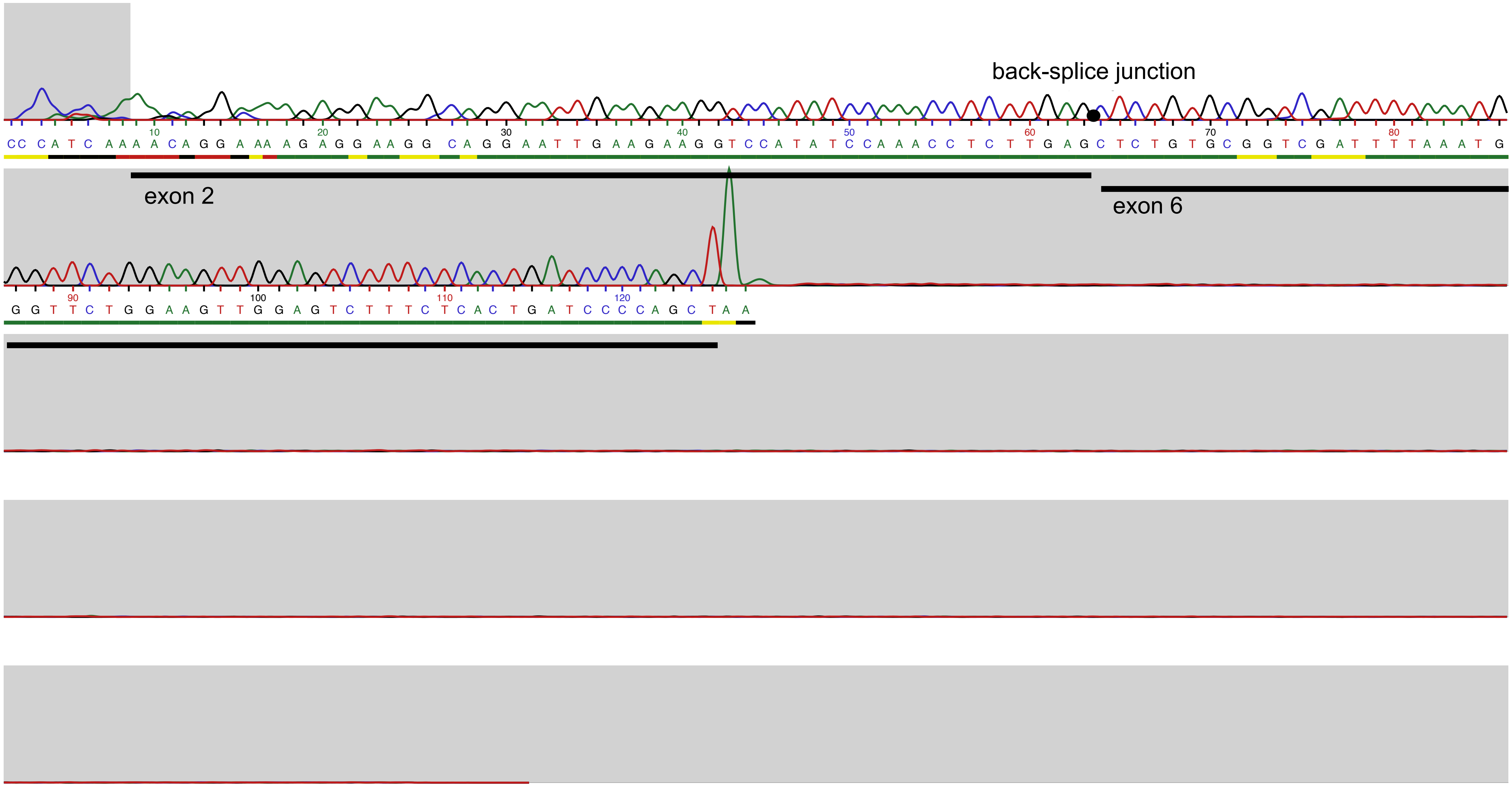

CDYL

Clip. 1 BQ 20 WL 10 Sequence: CDYL-1

Clipped length:	91	Samples:	12964	Quality:	0 - 9	Page: 1 / 3
Left clip:	44	Bases:	134		10 - 19	19.08.2019
Right clip:	134	Average spacing:	97.0		20 - 29	
Avg. qual. in clip.:	36.69	Average quality >=	10: 17, 20: 19, 30: 72		>= 30	

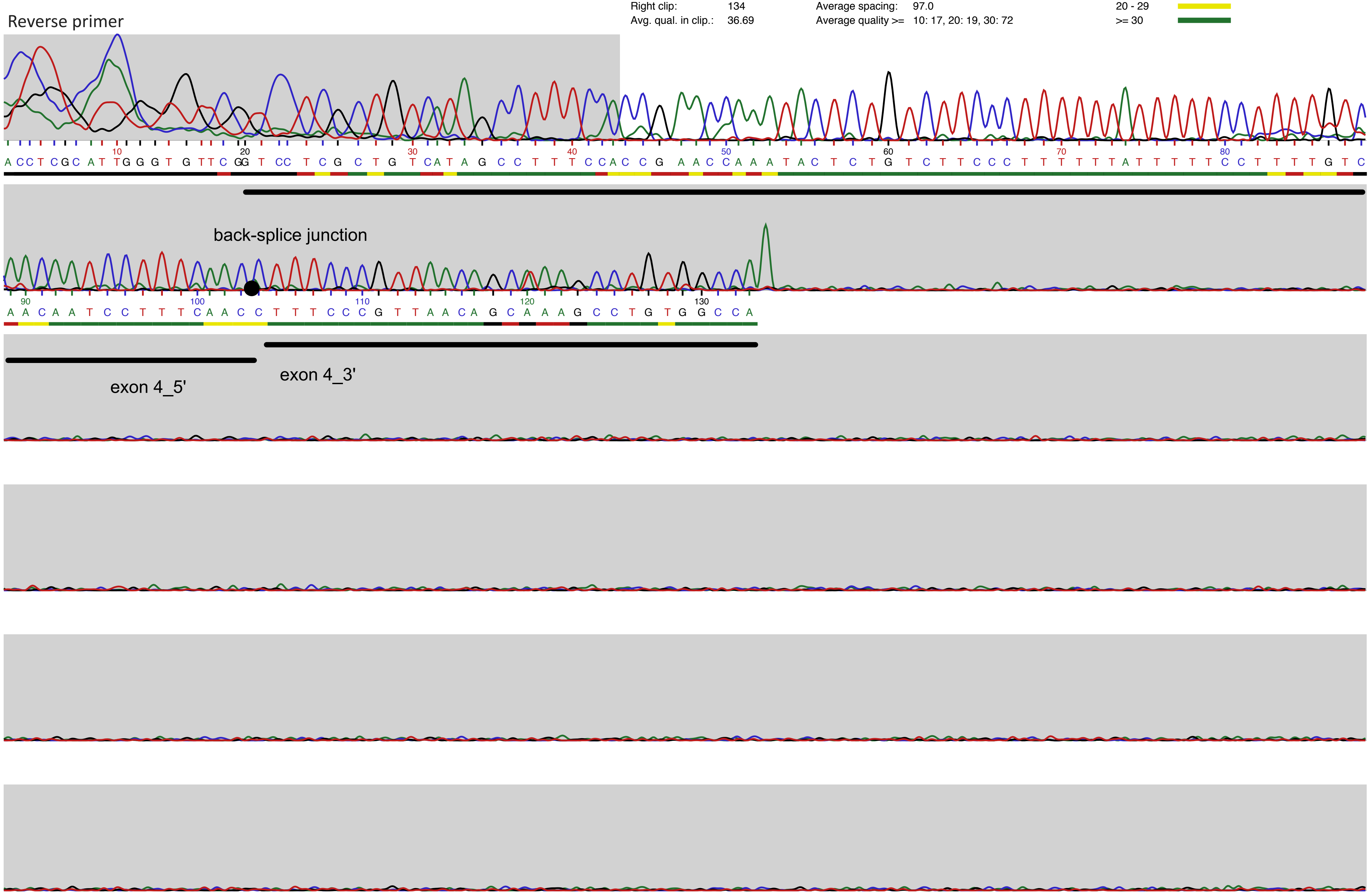

CORO1C

Clip. 1 BQ 20 WL 10 Sequence: CORO1C-1

Clipped length:	112	Samples:	12967	Quality: 0 - 9	
Left clip:	14	Bases:	250	10 - 19	
Right clip:	125	Average spacing:	52.0	20 - 29	
Avg. qual. in clip.:	47.01	Average quality >=	10: 73, 20: 28, 30: 116	>= 30	

22.08.2019

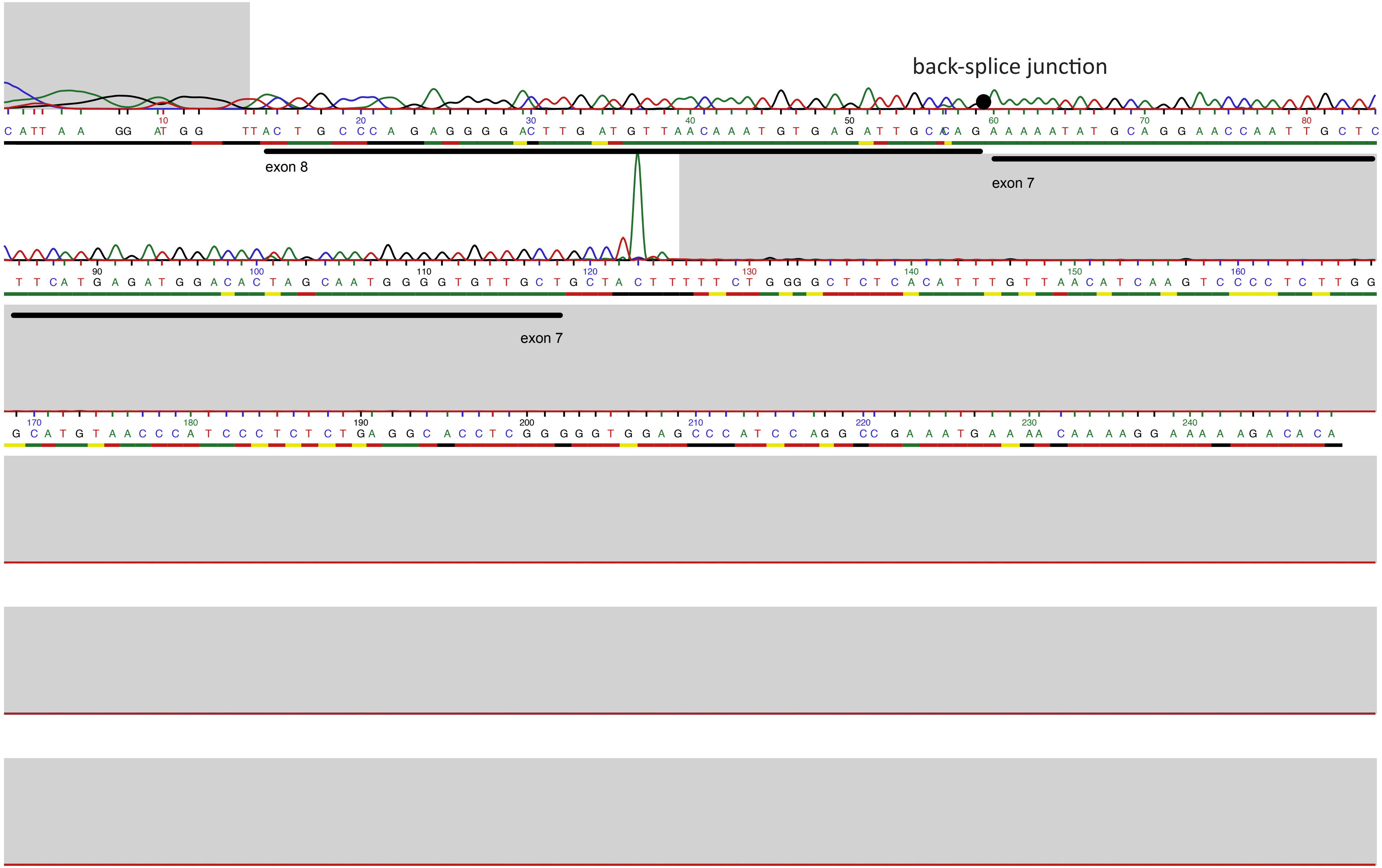

FAM13B

Clip. 1 BQ 20 WL 10 Sequence: FAM13B

Clipped length: 89
Left clip: 10
Right clip: 98
Avg. qual. in clip.: 46.61

Samples: 12960
Bases: 419
Average spacing: 31.0
Average quality >= 10: 227, 20: 47, 30: 83

Quality: 0 - 9, 10 - 19, 20 - 29, >= 30

13.09.2019

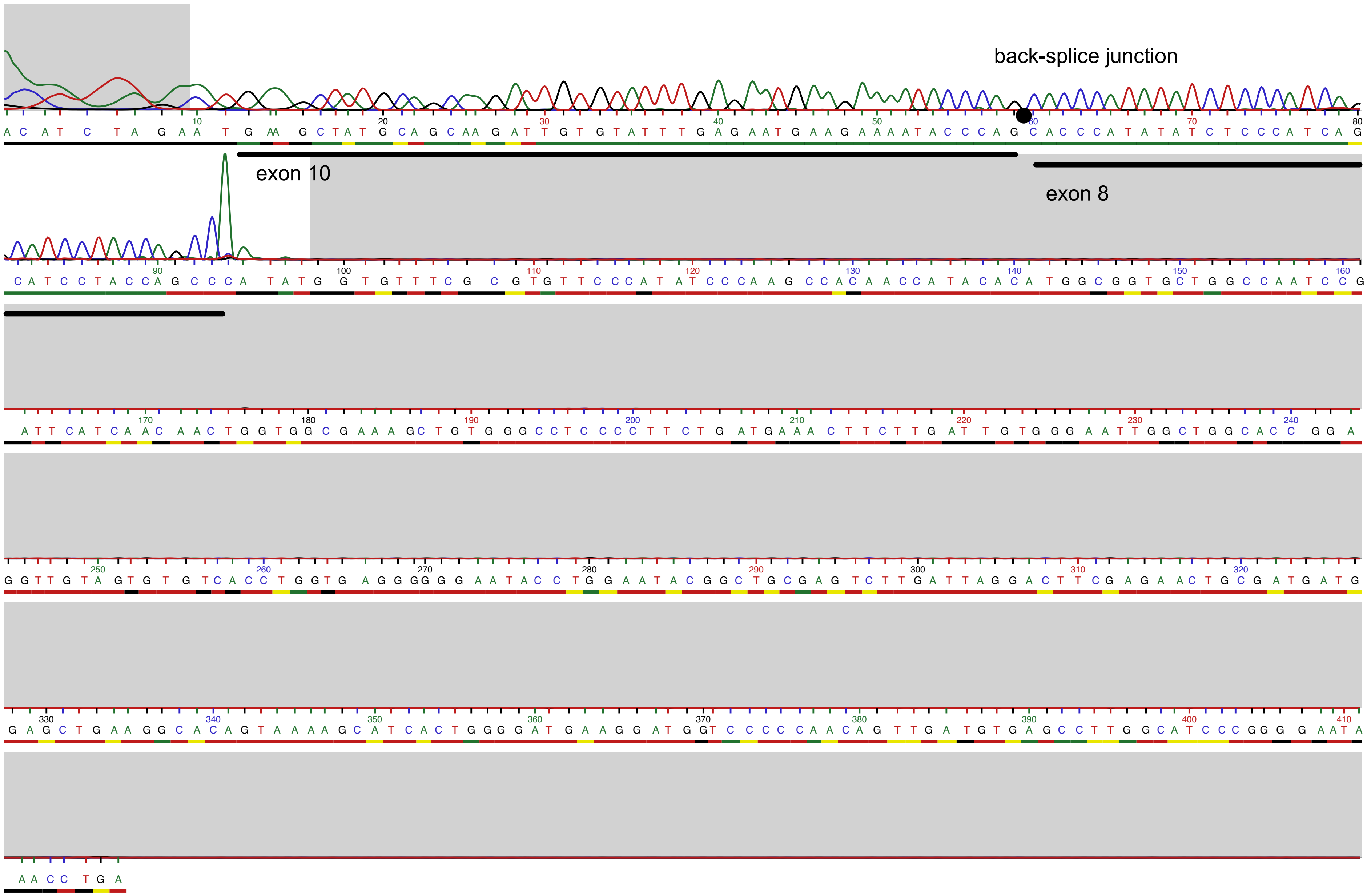

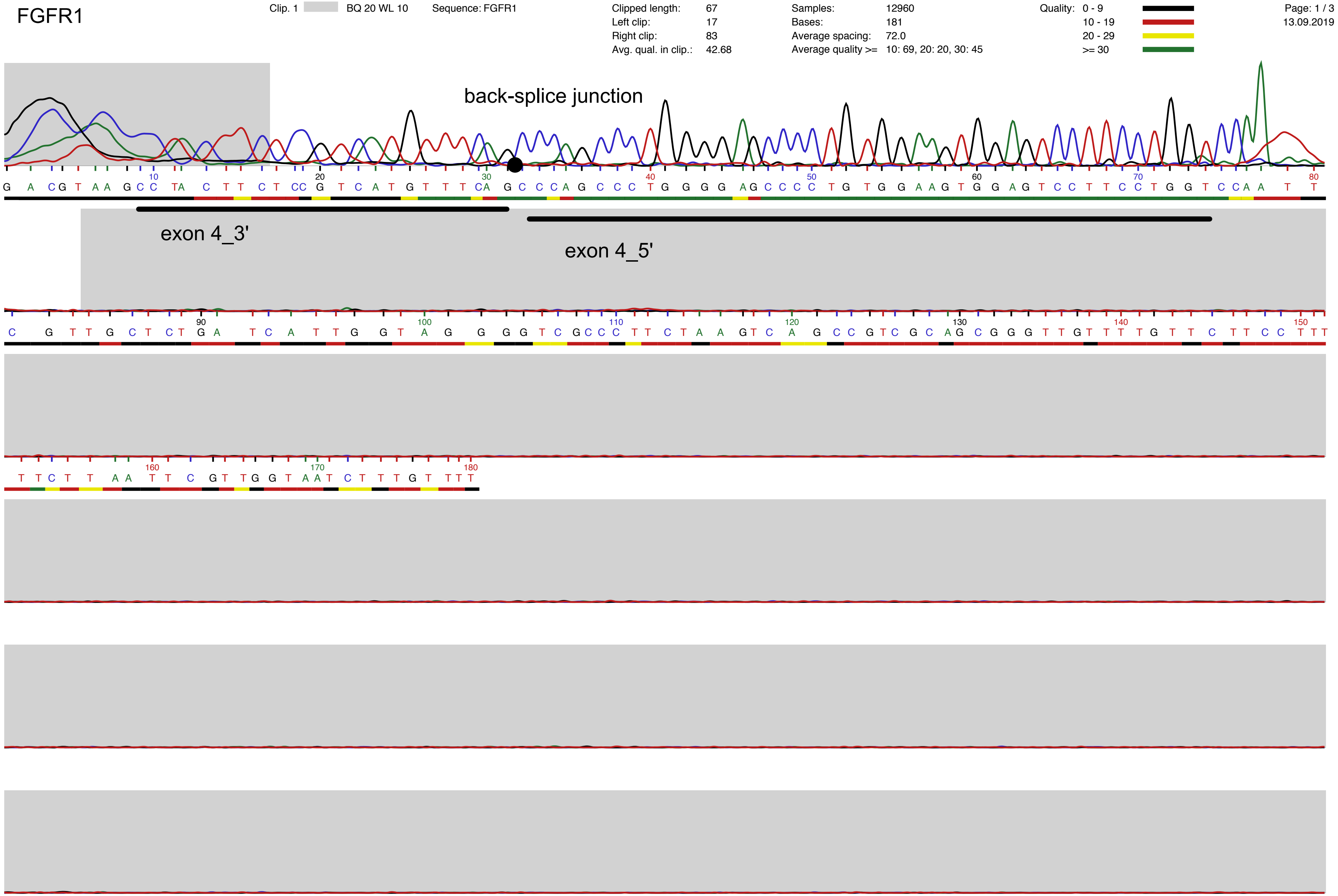

FGFR1
Clip. 1
BQ 20 WL 10
Sequence: FGFR1
Clipped length: 67
Left clip: 17
Right clip: 83
Avg. qual. in clip.: 42.68
Samples: 12960
Bases: 181
Average spacing: 72.0
Average quality >= 10: 69, 20: 20, 30: 45
Quality: 0 - 9
10 - 19
20 - 29
>= 30
Page: 1 / 3
13.09.2019
back-splice junction
exon 4_3'
exon 4_5'

FKBP8

Clip. 1 BQ 20 WL 10 Sequence: FKBP8-1

Clipped length:	93	Samples:	12964	Quality: 0 - 9	
Left clip:	16	Bases:	557	10 - 19	
Right clip:	108	Average spacing:	24.0	20 - 29	
Avg. qual. in clip.:	46.32	Average quality >=	10: 202, 20: 155, 30: 158	>= 30	

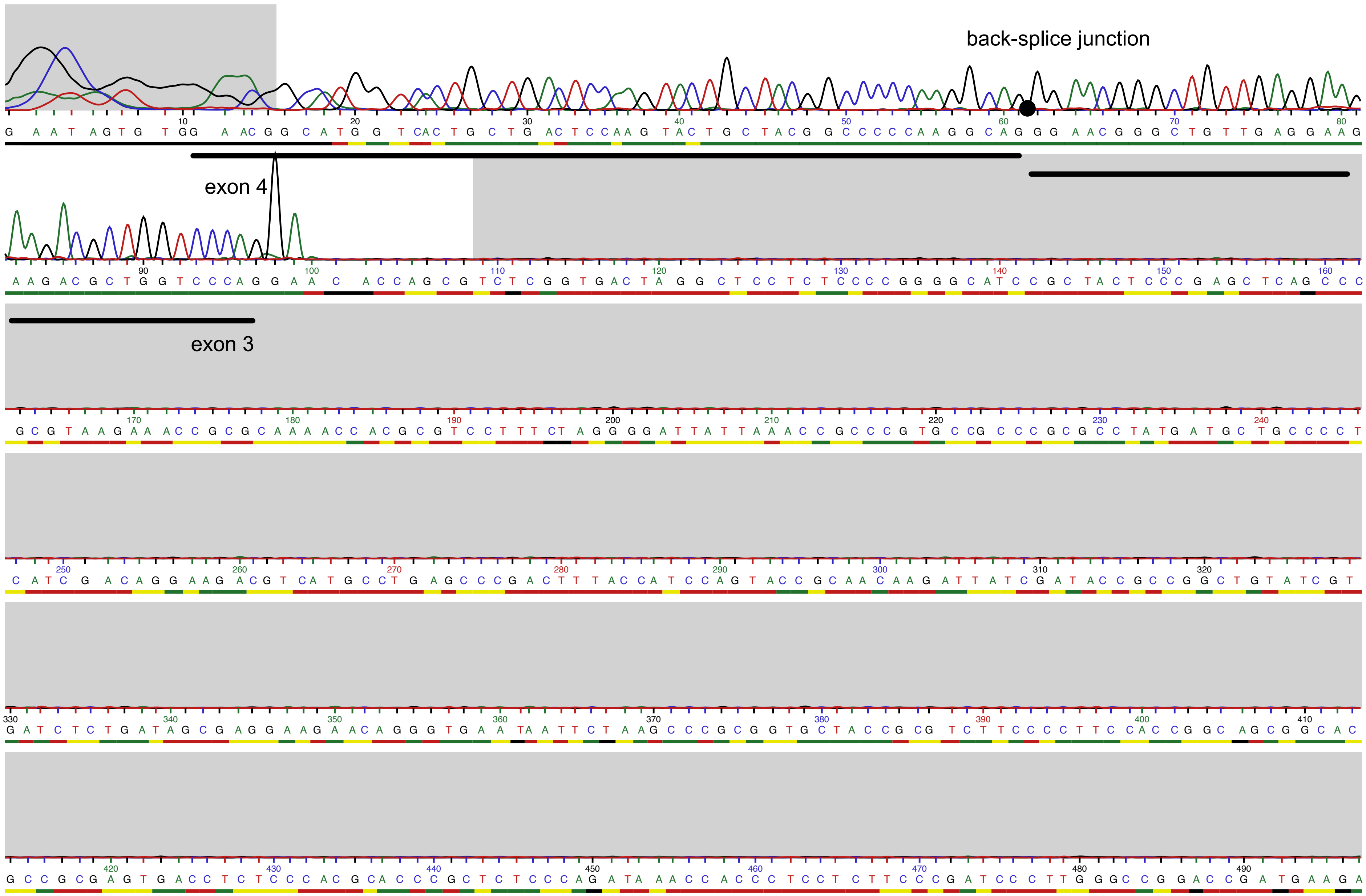

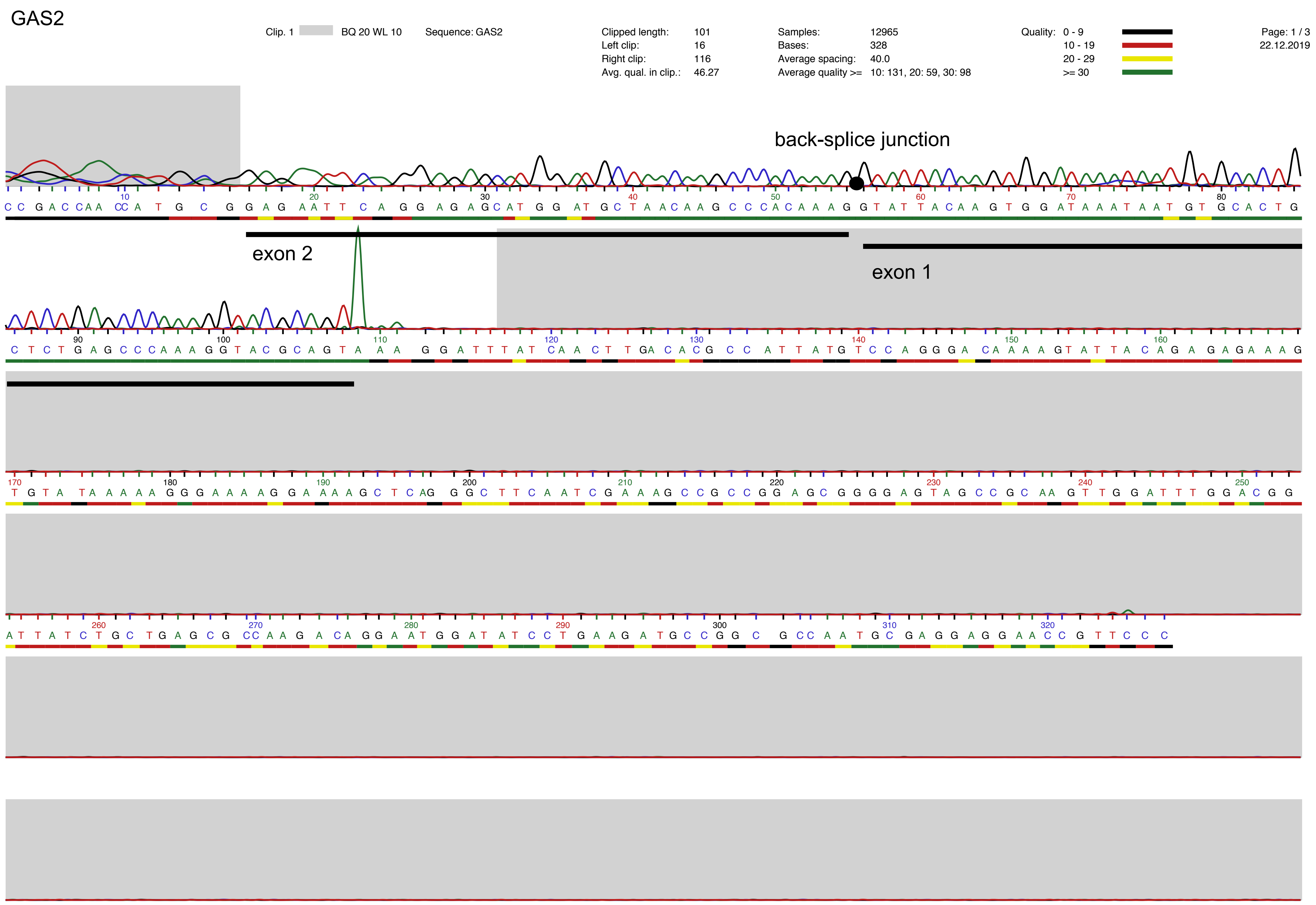
GAS2
Clip. 1 BQ 20 WL 10 Sequence: GAS2
Clipped length: 101
Left clip: 16
Right clip: 116
Avg. qual. in clip.: 46.27
Samples: 12965
Bases: 328
Average spacing: 40.0
Average quality >= 10: 131, 20: 59, 30: 98
Quality: 0 - 9
10 - 19
20 - 29
>= 30
Page: 1 / 3
22.12.2019
back-splice junction
exon 2
exon 1

GLIS1

Clip. 1 | BQ 20 WL 10 | Sequence: GLIS1-1

Clipped length:	67	Samples:	12964	Quality: 0 - 9		Page: 1 / 3
Left clip:	18	Bases:	148	10 - 19		27.08.2019
Right clip:	84	Average spacing:	88.0	20 - 29		
Avg. qual. in clip.:	42.56	Average quality >=	10: 55, 20: 10, 30: 47	>= 30		

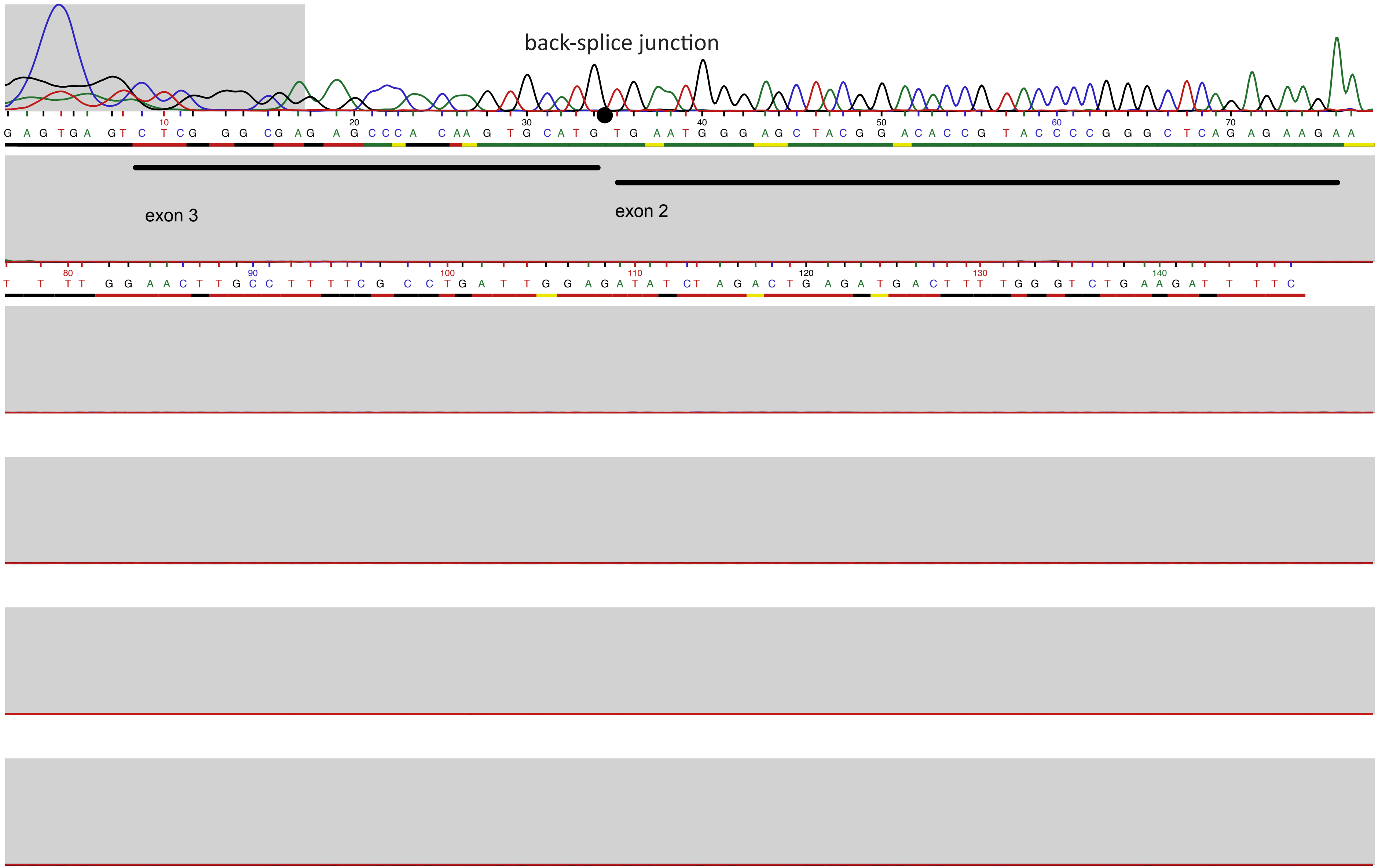

GLIS2

reverse primer

Clip. 1 BQ 20 WL 10 Sequence: GLIS2-1

Clipped length:	91	Samples:	12964	Quality: 0 - 9	
Left clip:	13	Bases:	147	10 - 19	
Right clip:	103	Average spacing:	89.0	20 - 29	
Avg. qual. in clip.:	34.0	Average quality >=	10: 24, 20: 26, 30: 57	>= 30	

27.08.2019

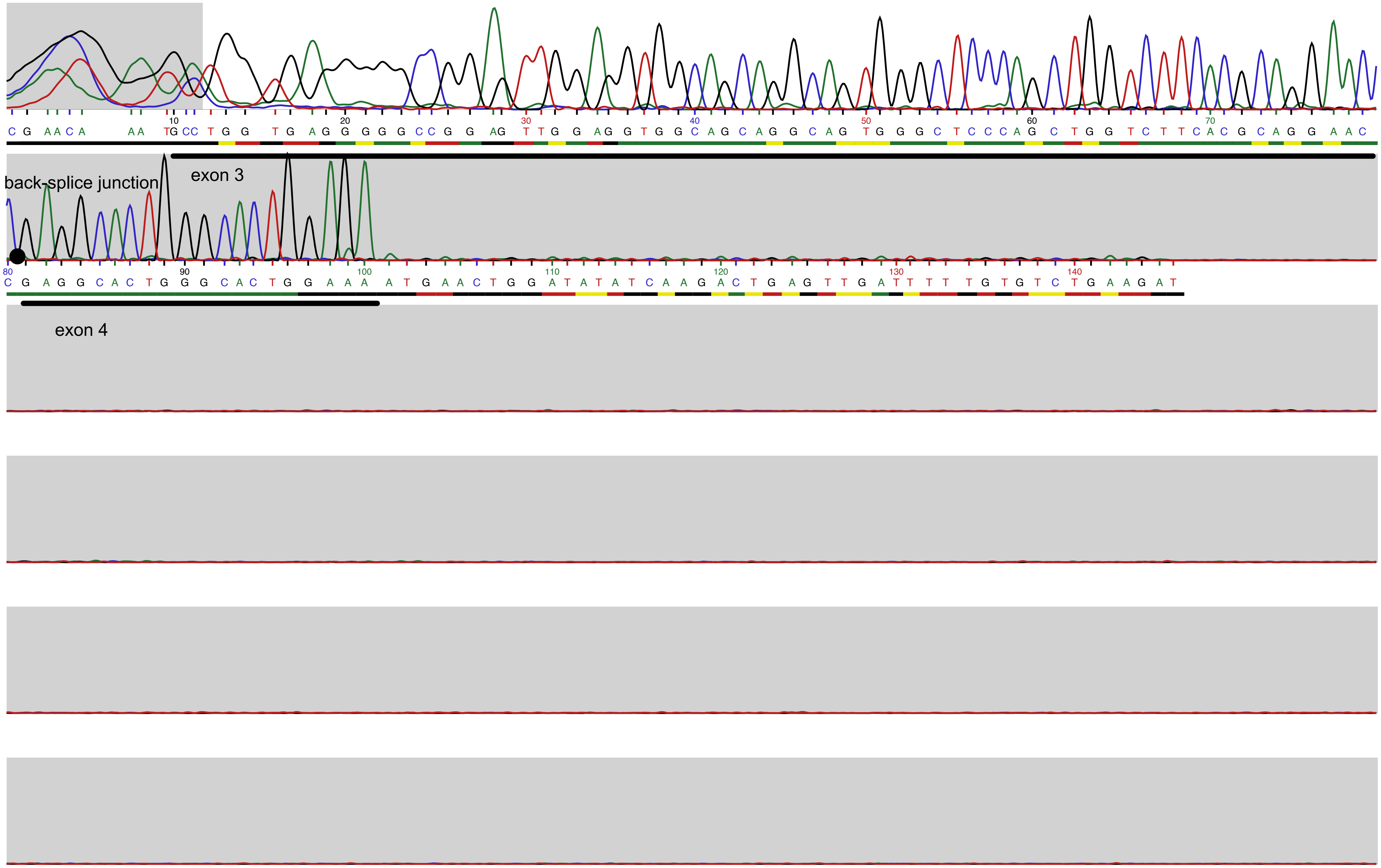

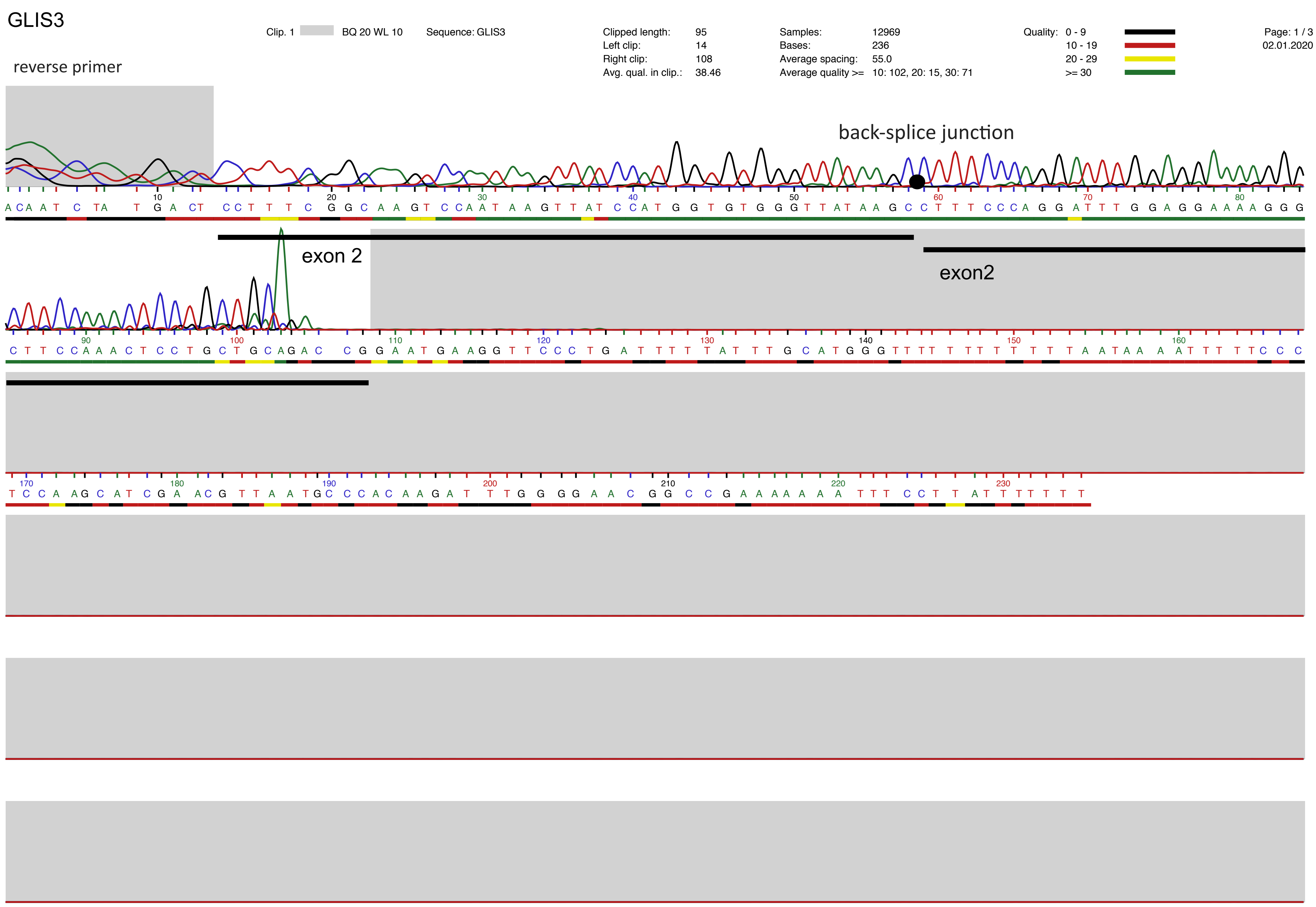

GLIS3
Clip. 1
BQ 20 WL 10
Sequence: GLIS3
Clipped length: 95
Left clip: 14
Right clip: 108
Avg. qual. in clip.: 38.46
Samples: 12969
Bases: 236
Average spacing: 55.0
Average quality >= 10: 102, 20: 15, 30: 71
Quality: 0 - 9
10 - 19
20 - 29
>= 30
Page: 1 / 3
02.01.2020
reverse primer
back-splice junction
exon 2
exon2

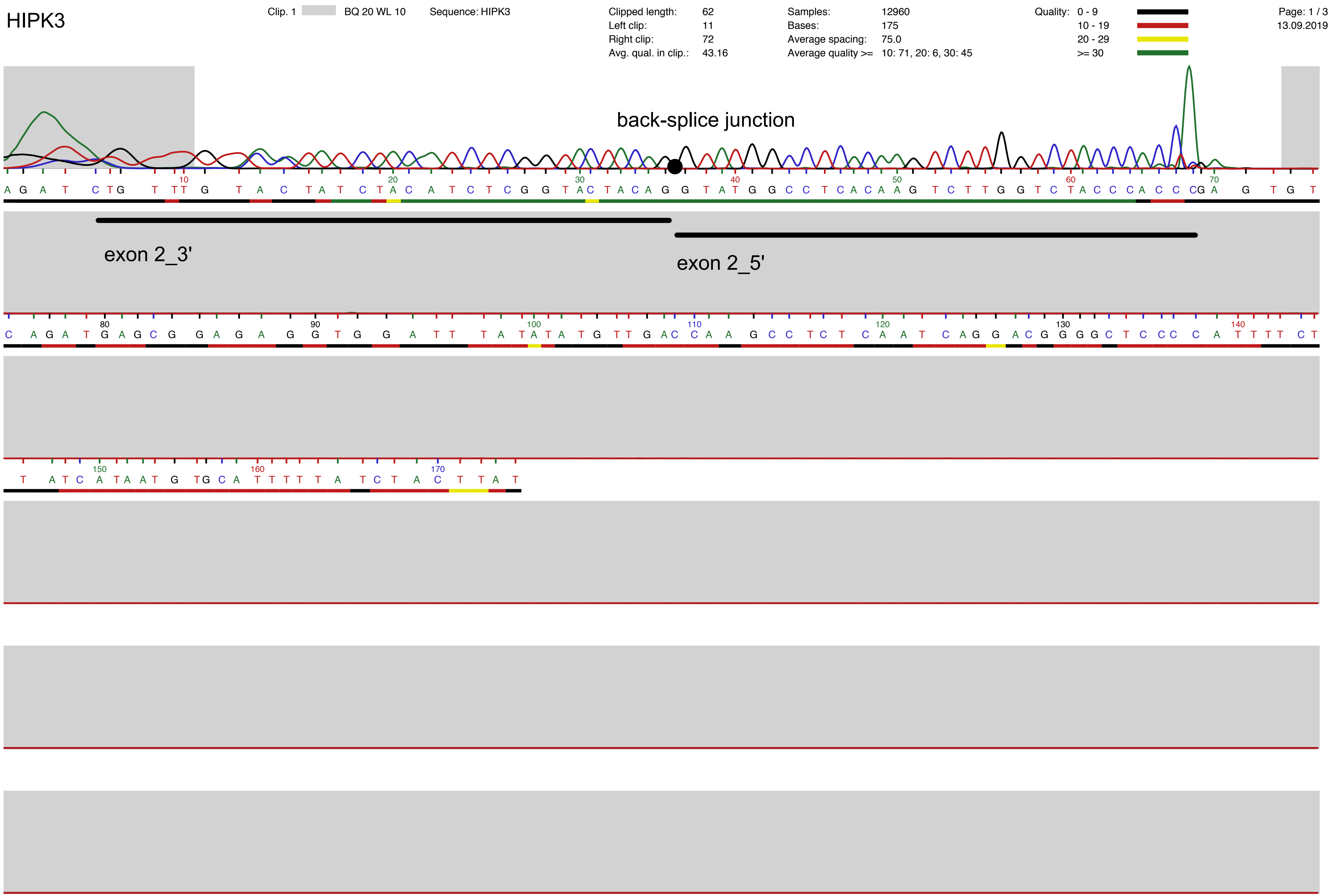

HIPK3
Clip. 1
BQ 20 WL 10
Sequence: HIPK3
Clipped length: 62
Left clip: 11
Right clip: 72
Avg. qual. in clip.: 43.16
Samples: 12960
Bases: 175
Average spacing: 75.0
Average quality >= 10: 71, 20: 6, 30: 45
Quality: 0 - 9
10 - 19
20 - 29
>= 30
Page: 1 / 3
13.09.2019
back-splice junction
AGATCTGTTTGTACTATCTACATCTCGGTACTACAGGTATGGCCTCACAAGTCTTGGTCTACCCACCCGAGTGT
exon 2_3'
exon 2_5'
CAGATGAGCGGAGAGGTGGATTTATATATGTTGACCAAGCCTCTCAATCAGGACGGGCTCCCCATTTTCT
TATCATAATGTGCATTTTTATCTACTTAT

LPAR1

Clip. 1 | BQ 20 WL 10 | Sequence: LPAR1

Clipped length:	90	Samples:	12960	Quality: 0 - 9	
Left clip:	12	Bases:	166	10 - 19	
Right clip:	101	Average spacing:	79.0	20 - 29	
Avg. qual. in clip.:	46.01	Average quality >=	10: 52, 20: 6, 30: 72	>= 30	

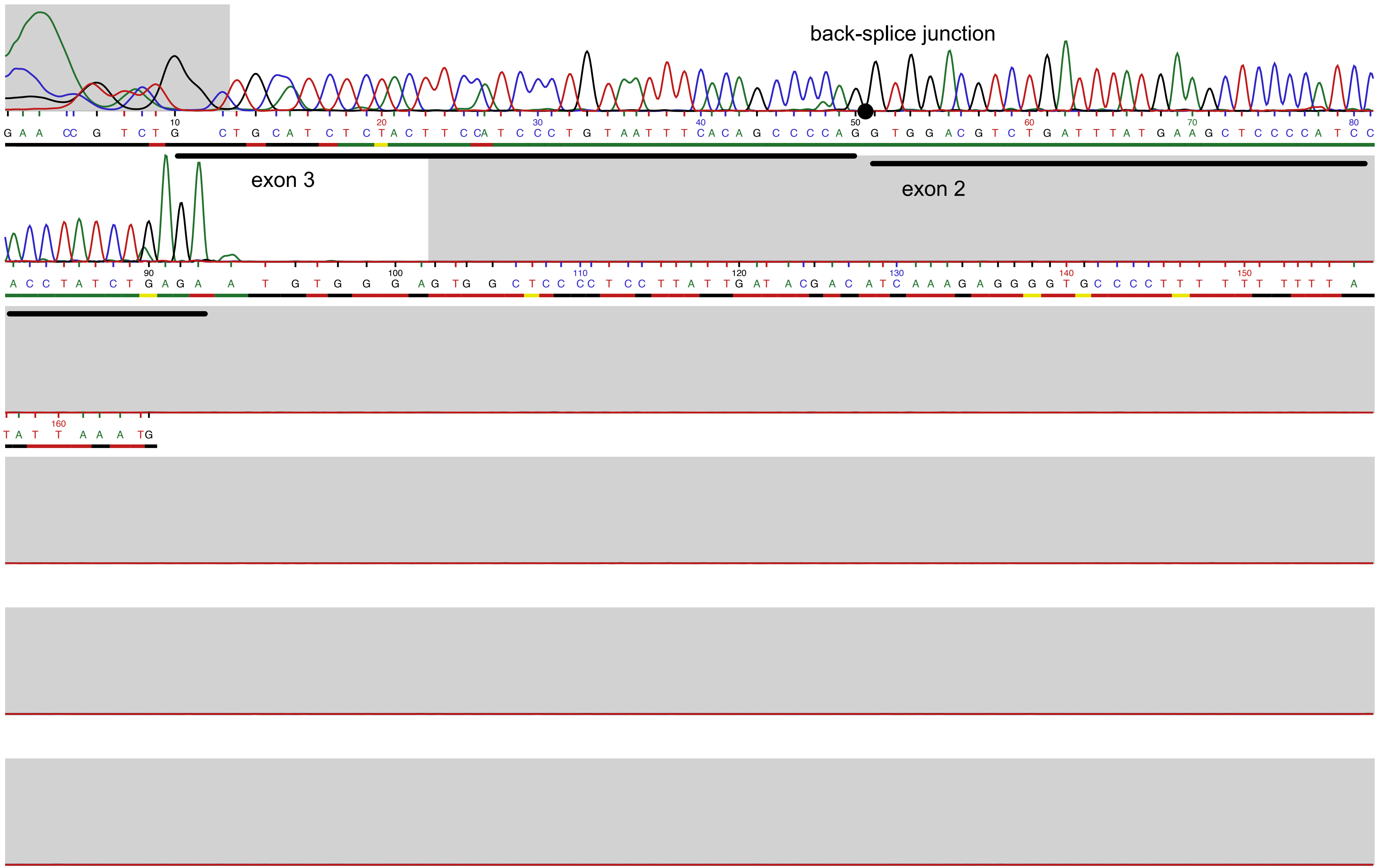

LRBA

Clip. 1 | BQ 20 WL 10 | Sequence: LRBA

Clipped length: 97
Left clip: 12
Right clip: 108
Avg. qual. in clip.: 47.13

Samples: 12961
Bases: 173
Average spacing: 75.0
Average quality >= 10: 62, 20: 8, 30: 76

Quality: 0 - 9, 10 - 19, 20 - 29, >= 30

13.09.2019

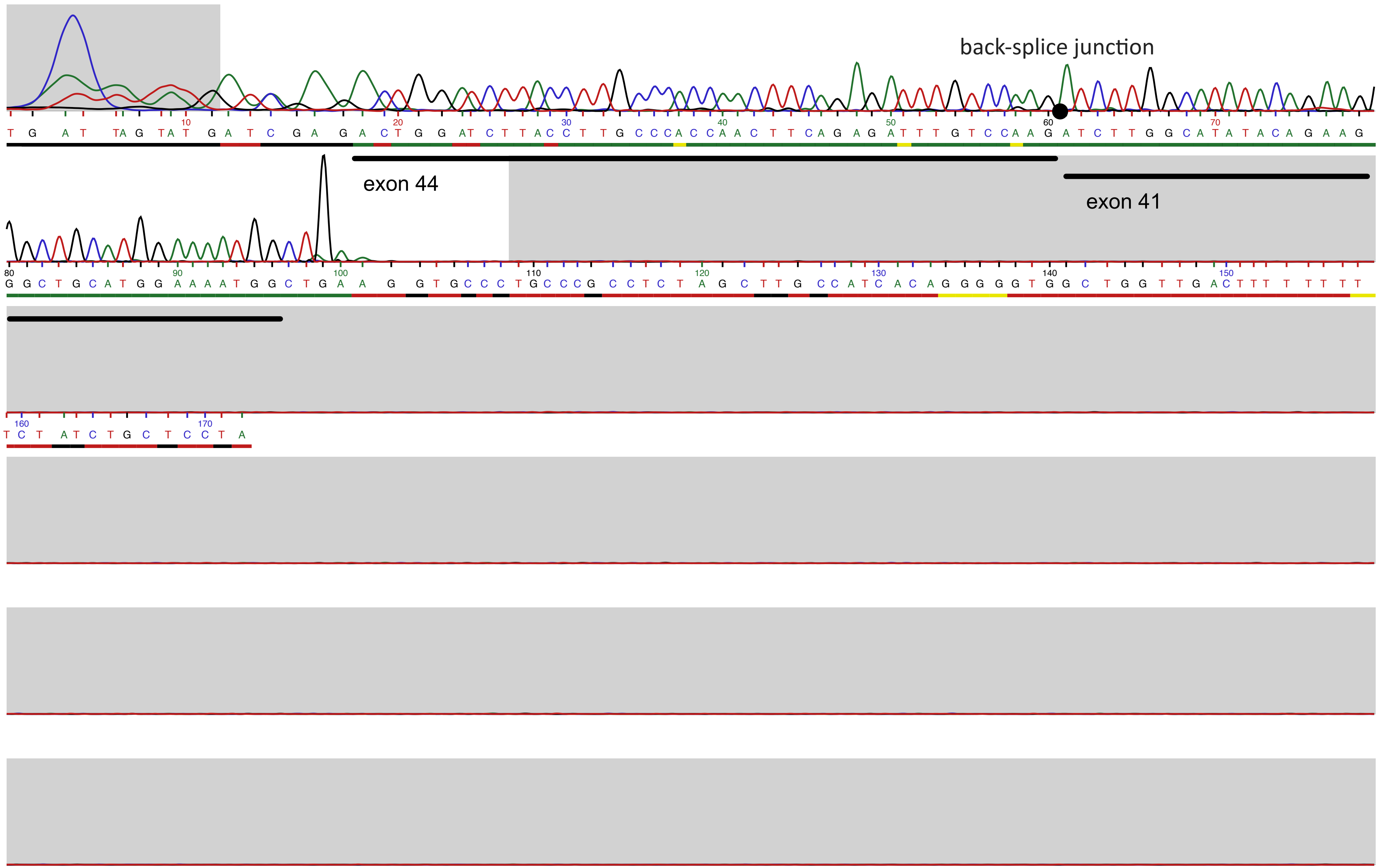

MARK4
reverse primer

Clip. 1 BQ 20 WL 10 Sequence: MARK4-1

Clipped length: 65
Left clip: 15
Right clip: 79
Avg. qual. in clip.: 38.36

Samples: 12964
Bases: 360
Average spacing: 37.0
Average quality >= 10: 221, 20: 25, 30: 43

Quality: 0 - 9
10 - 19
20 - 29
>= 30

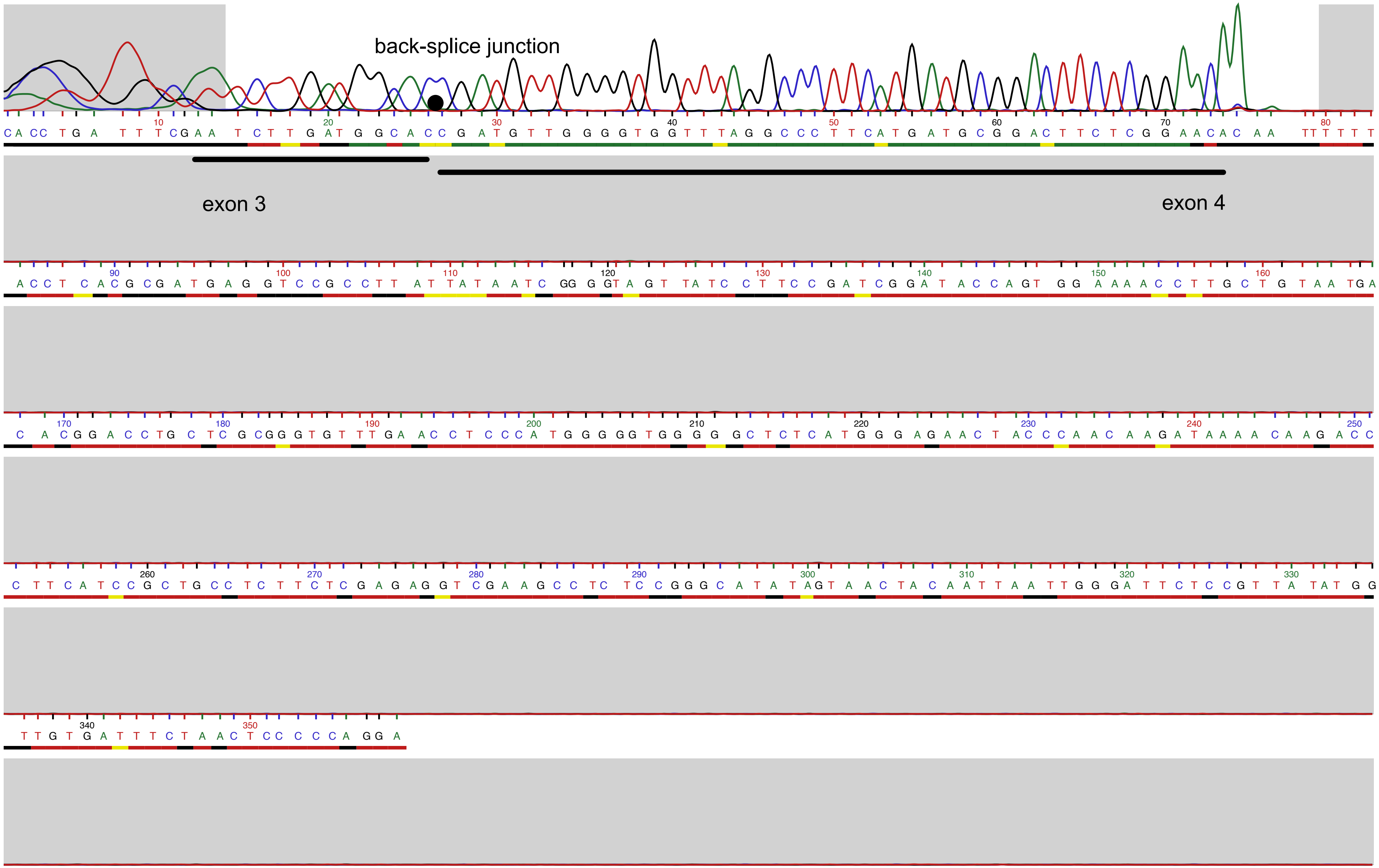

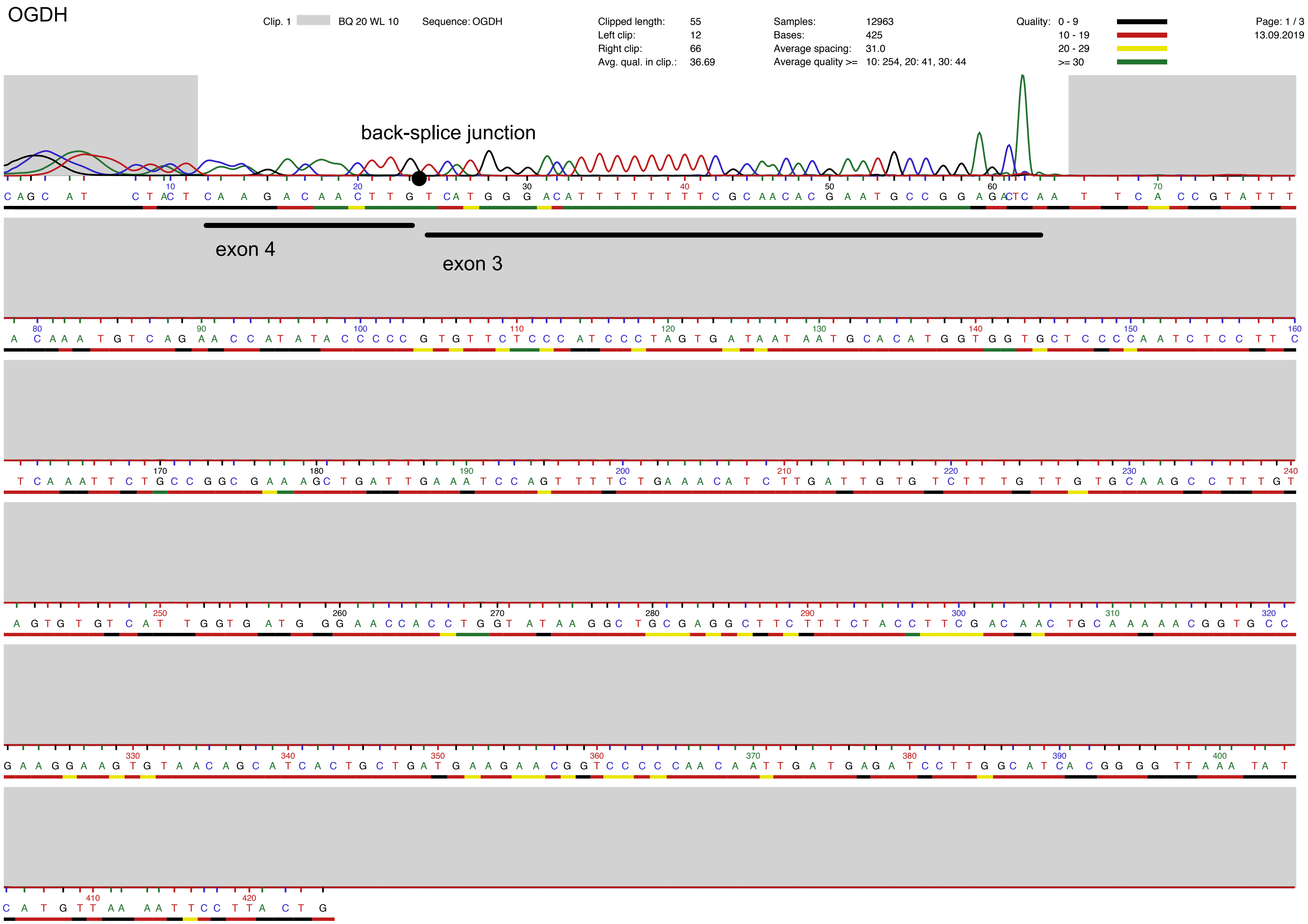
OGDH
Clip. 1
BQ 20 WL 10
Sequence: OGDH
Clipped length: 55
Left clip: 12
Right clip: 66
Avg. qual. in clip.: 36.69
Samples: 12963
Bases: 425
Average spacing: 31.0
Average quality >= 10: 254, 20: 41, 30: 44
Quality: 0 - 9
10 - 19
20 - 29
>= 30
Page: 1 / 3
13.09.2019
back-splice junction
exon 4
exon 3

RARS

reverse primer

Clip. 1 BQ 20 WL 10 Sequence: RARS

Clipped length:	72	Samples:	12963	Quality: 0 - 9	Page: 1 / 3
Left clip:	11	Bases:	413	10 - 19	13.09.2019
Right clip:	82	Average spacing:	32.0	20 - 29	
Avg. qual. in clip.:	44.93	Average quality >=	10: 225, 20: 48, 30: 66	>= 30	

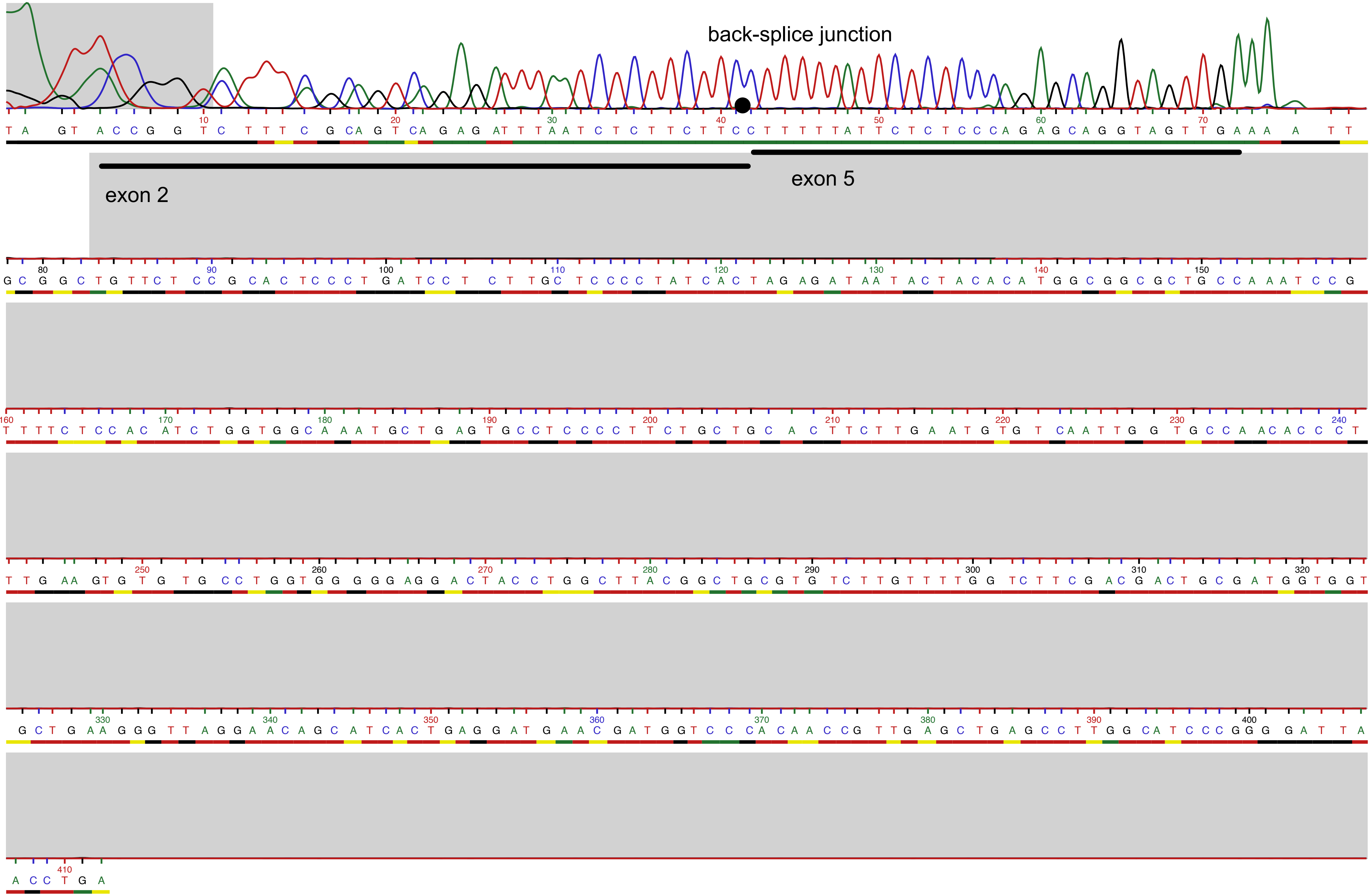

RHOBTB3

Sequence: RHOBTB3-1

Samples:	13007	Quality:	0 - 9	
Bases:	5		10 - 19	
Average spacing:	2602.0		20 - 29	
Average quality >=	10: 0, 20: 0, 30: 0		>= 30	

22.08.2019

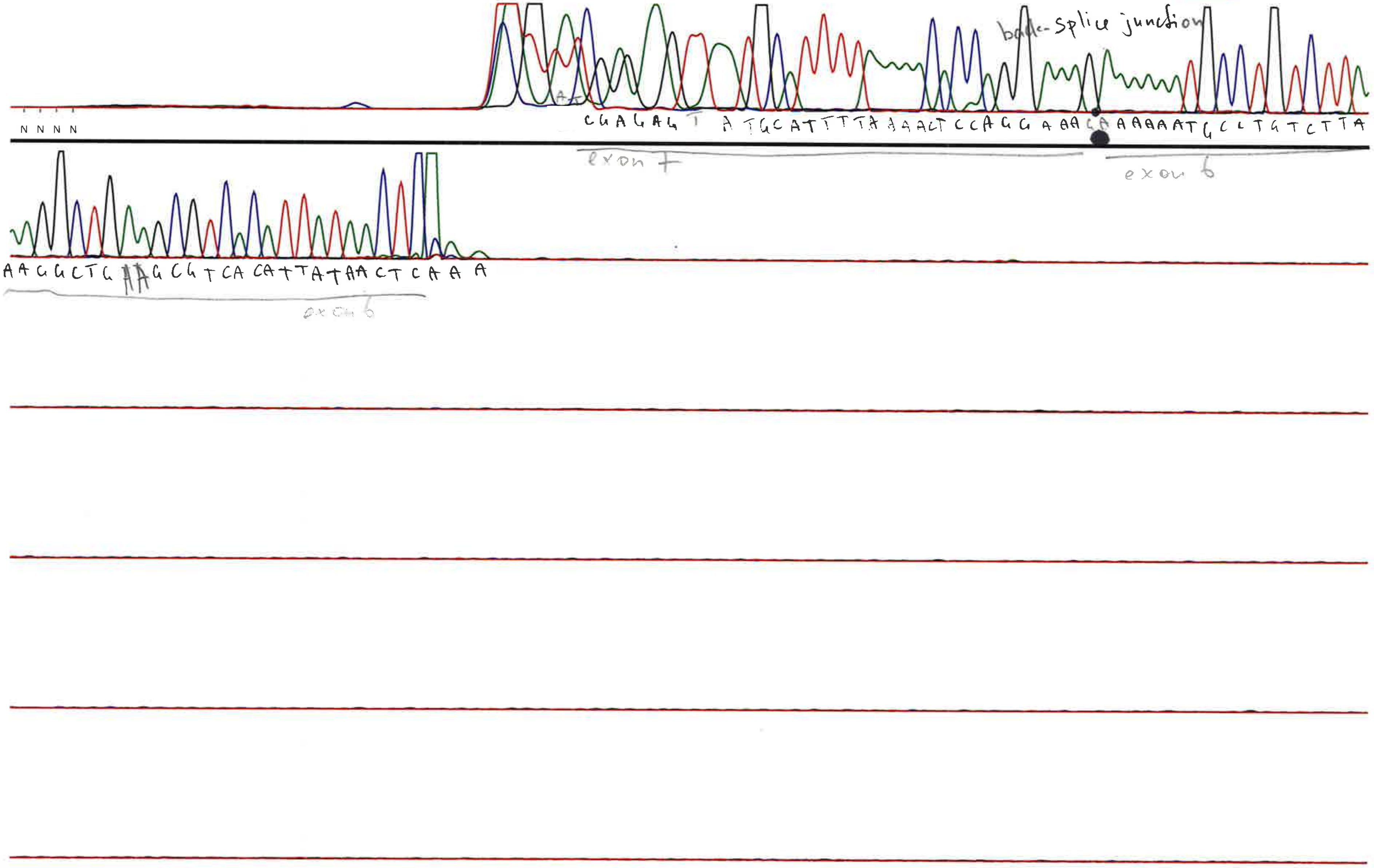

RSRC1

Clip. 1 BQ 20 WL 10 Sequence: RSRC1

Clipped length: 36
Left clip: 18
Right clip: 53
Avg. qual. in clip.: 27.52

Samples: 12965
Bases: 608
Average spacing: 22.0
Average quality >= 10: 386, 20: 69, 30: 38

Quality: 0 - 9
10 - 19
20 - 29
>= 30

22.02.2020

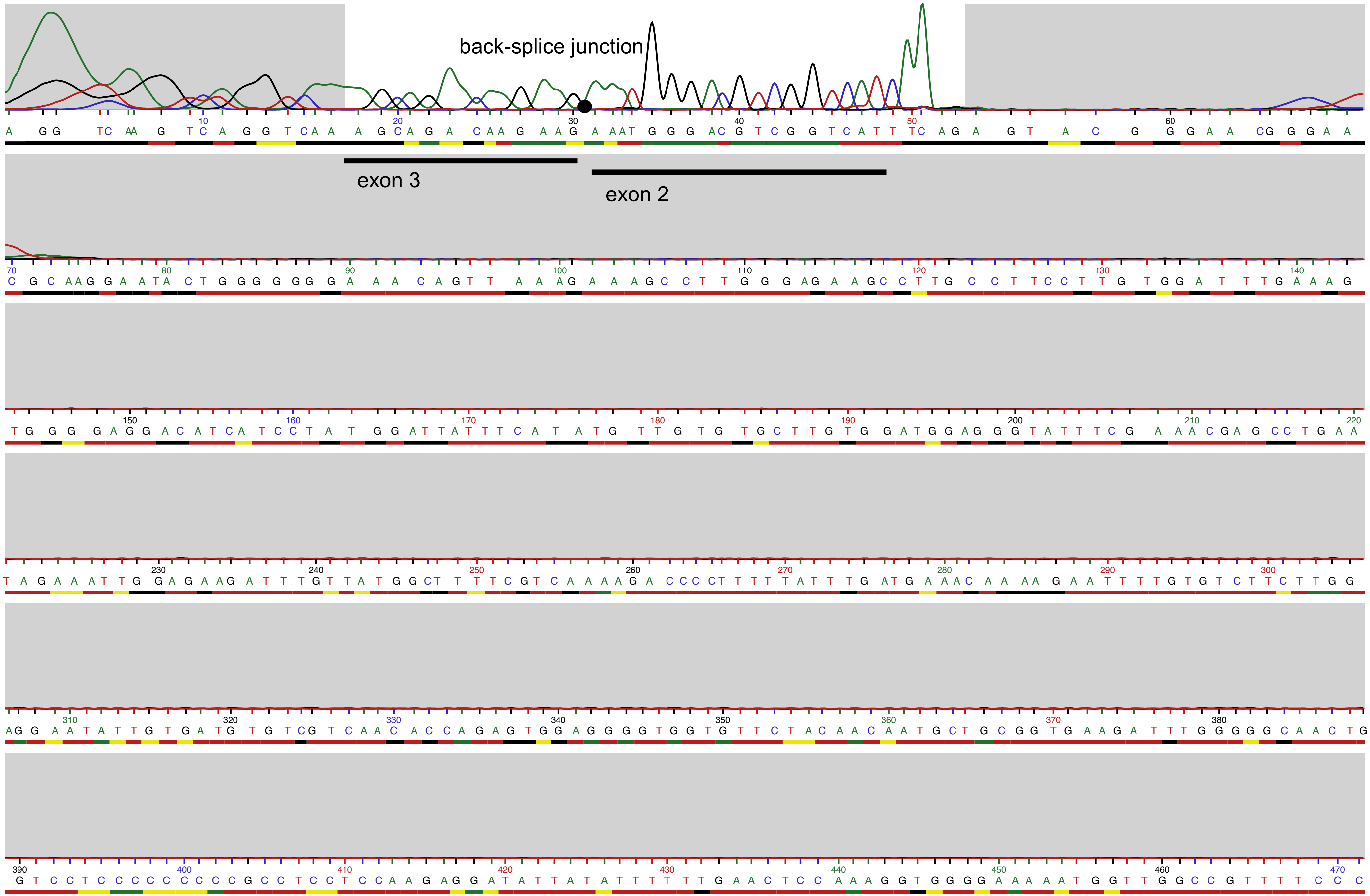

SFMBT2

reverse primer

Clip. 1 | BQ 20 WL 10 | Sequence: SFMBT

Clipped length:	67	Samples:	12966	Quality:	0 - 9	Page: 1 / 3
Left clip:	7	Bases:	357		10 - 19	22.12.2019
Right clip:	73	Average spacing:	37.0		20 - 29	
Avg. qual. in clip.:	39.82	Average quality >=	10: 152, 20: 69, 30: 97		>= 30	

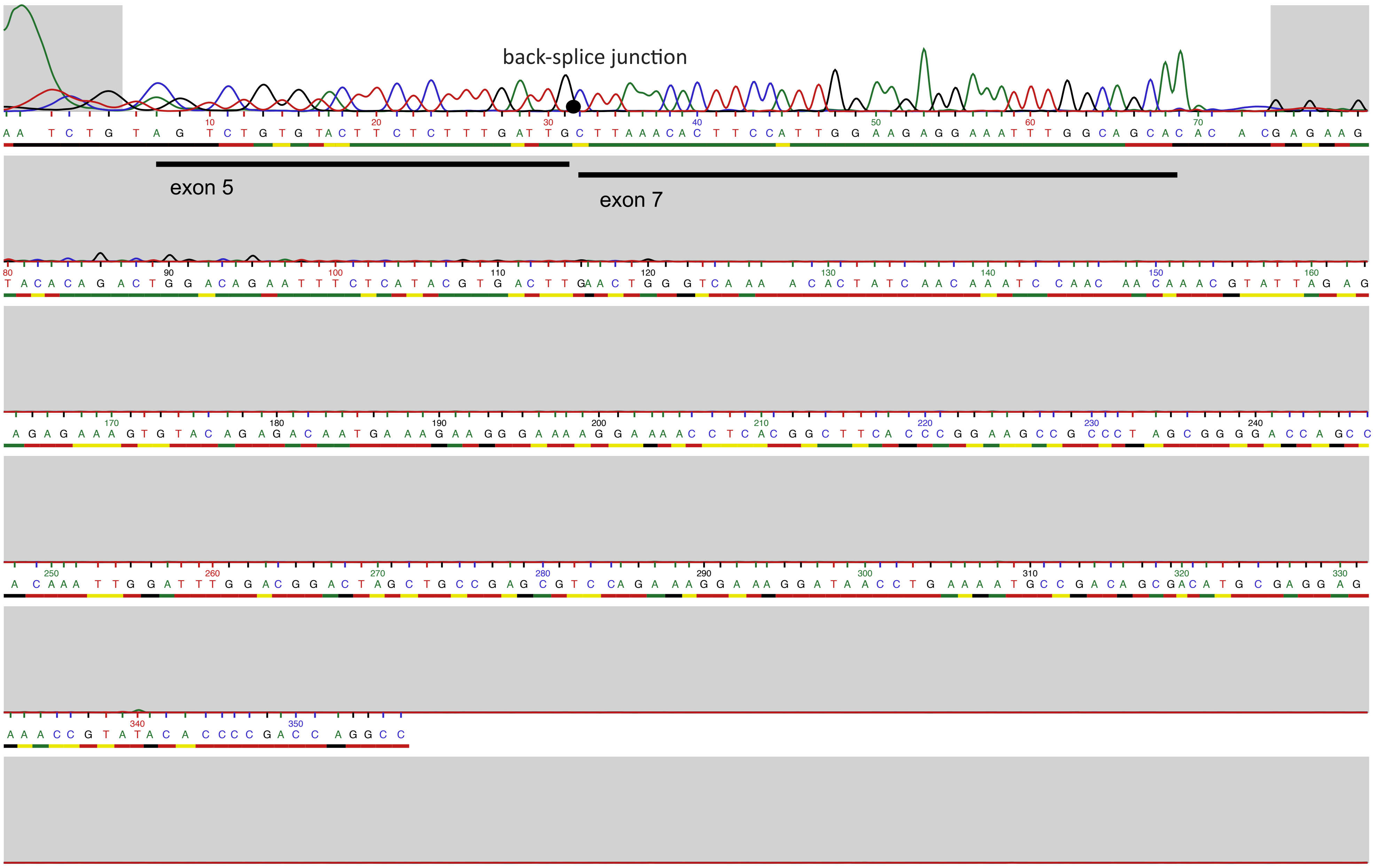

SMARCA5

Clip. 1 BQ 20 WL 10 Sequence: SMARCA5-1

Clipped length:	156	Samples:	12965	Quality: 0 - 9	
Left clip:	10	Bases:	165	10 - 19	
Right clip:	165	Average spacing:	79.0	20 - 29	
Avg. qual. in clip.:	55.37	Average quality >=	10: 4, 20: 3, 30: 145	>= 30	

22.08.2019

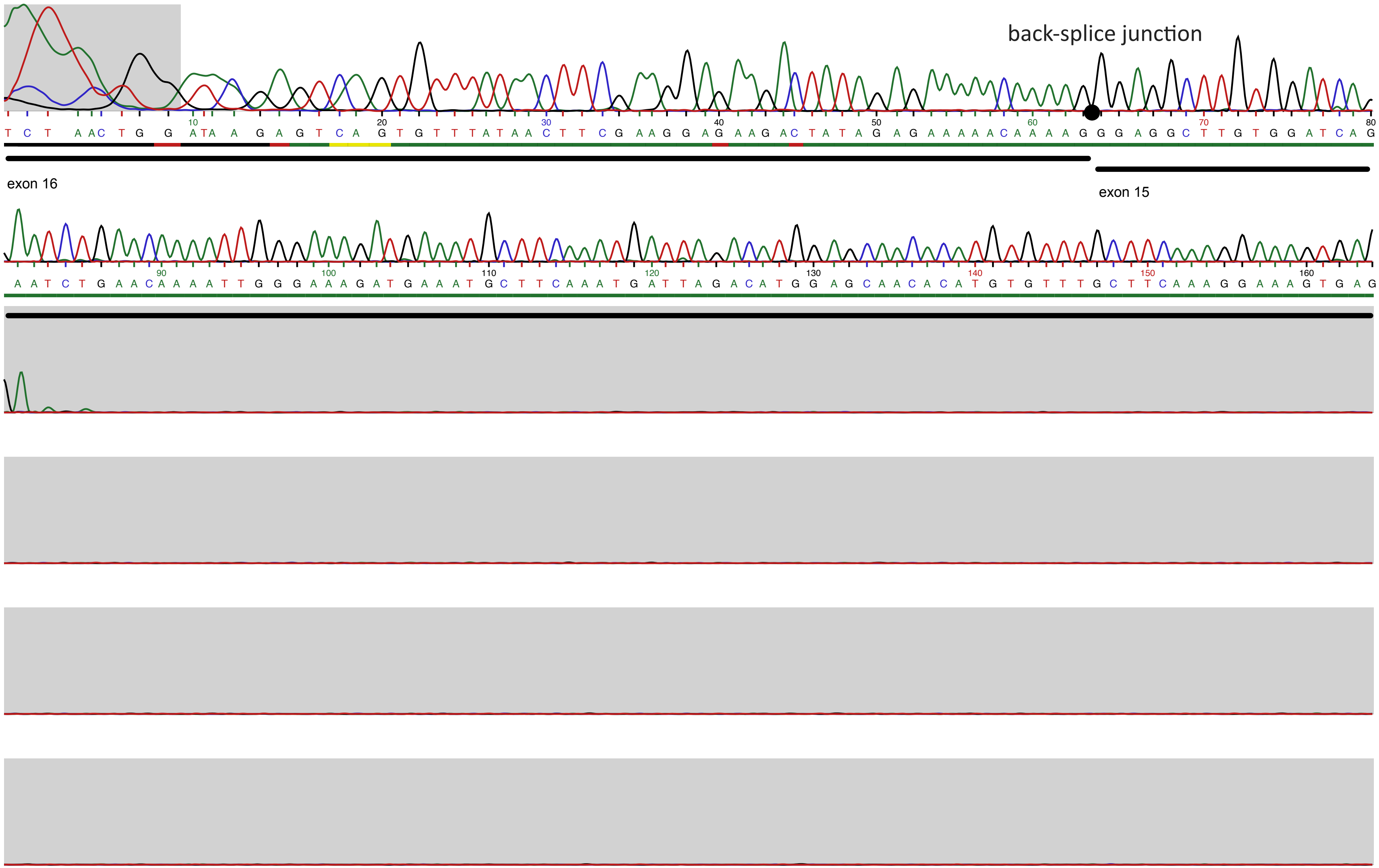

SMO

Reverse primer

Sequence: SMO-1

Samples: 13007
Bases: 5
Average spacing: 2602.0
Average quality >= 10: 0, 20: 0, 30: 0

Quality: 0 - 9, 10 - 19, 20 - 29, >= 30

27.08.2019

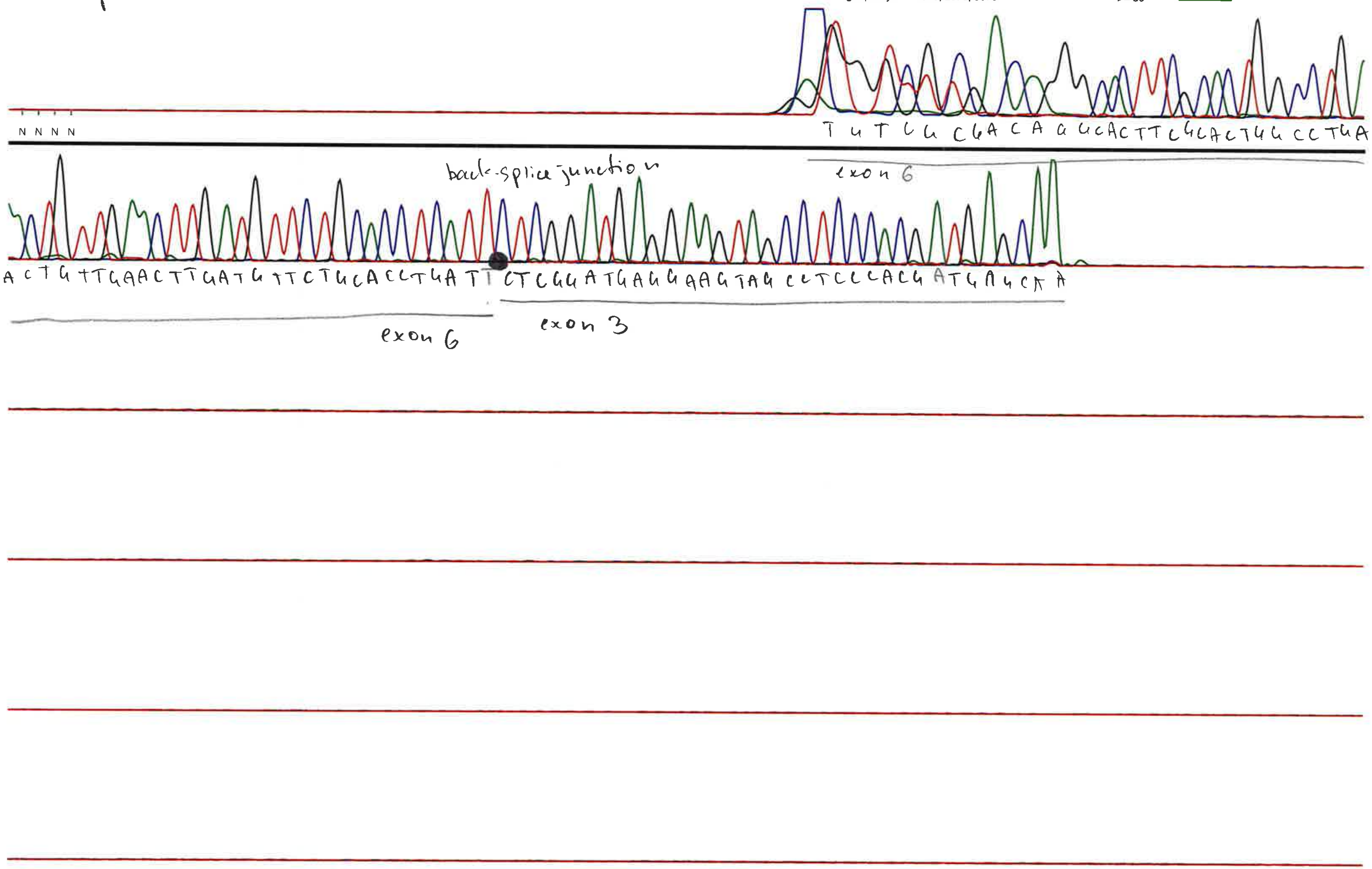

UBXN7

Clip. 1 BQ 20 WL 10 Sequence: UBXN7

Clipped length:	78	Samples:	12964	Quality: 0 - 9	
Left clip:	11	Bases:	155	10 - 19	
Right clip:	88	Average spacing:	84.0	20 - 29	
Avg. qual. in clip.:	45.24	Average quality >=	10: 55, 20: 1, 30: 57	>= 30	

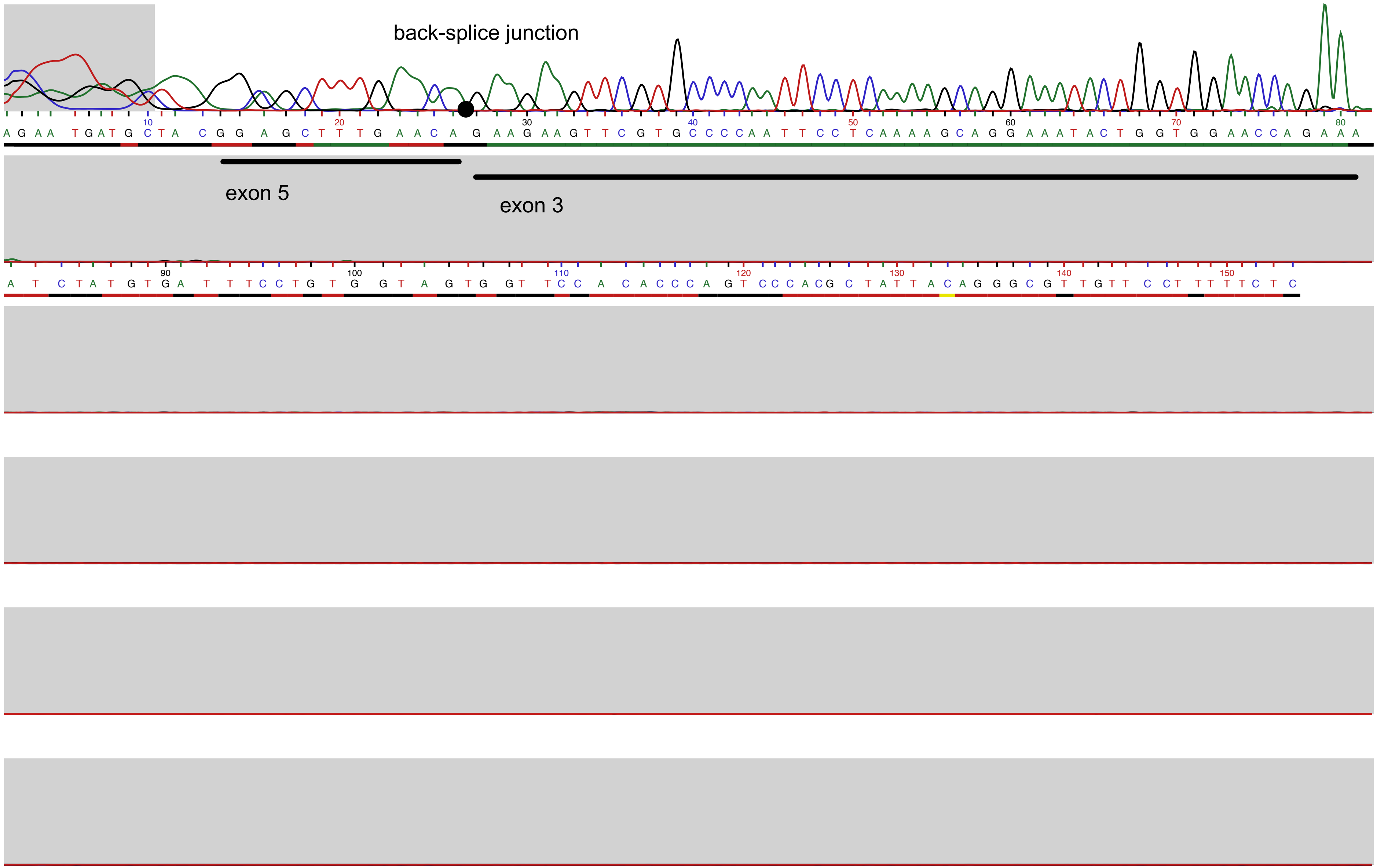

ZKSCAN1

Clip. 1 BQ 20 WL 10 Sequence: ZKSCAN1

Clipped length:	94	Samples:	12963	Quality:	0 - 9
Left clip:	10	Bases:	427		10 - 19
Right clip:	103	Average spacing:	31.0		20 - 29
Avg. qual. in clip.:	48.63	Average quality >=	10: 254, 20: 38, 30: 81		>= 30

13.09.2019

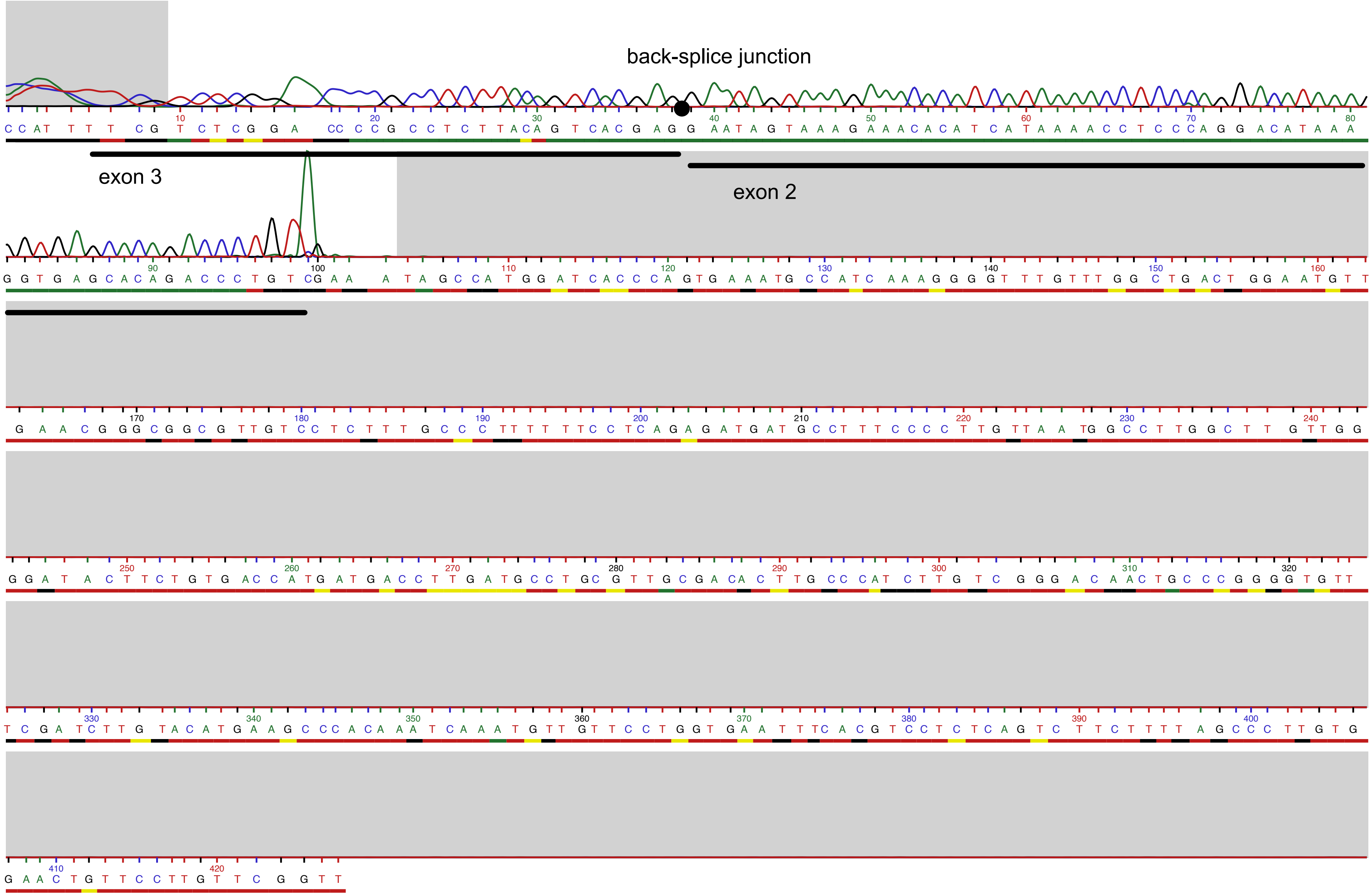

COL1A2

R-primer

Clip. 1 BQ 20 WL 10 Sequence: COL1A2-1

Clipped length:	15	Samples:	12964	Quality: 0 - 9	
Left clip:	100	Bases:	147	10 - 19	
Right clip:	114	Average spacing:	89.0	20 - 29	
Avg. qual. in clip.:	18.46	Average quality >=	10: 30, 20: 7, 30: 6	>= 30	

22.08.2019

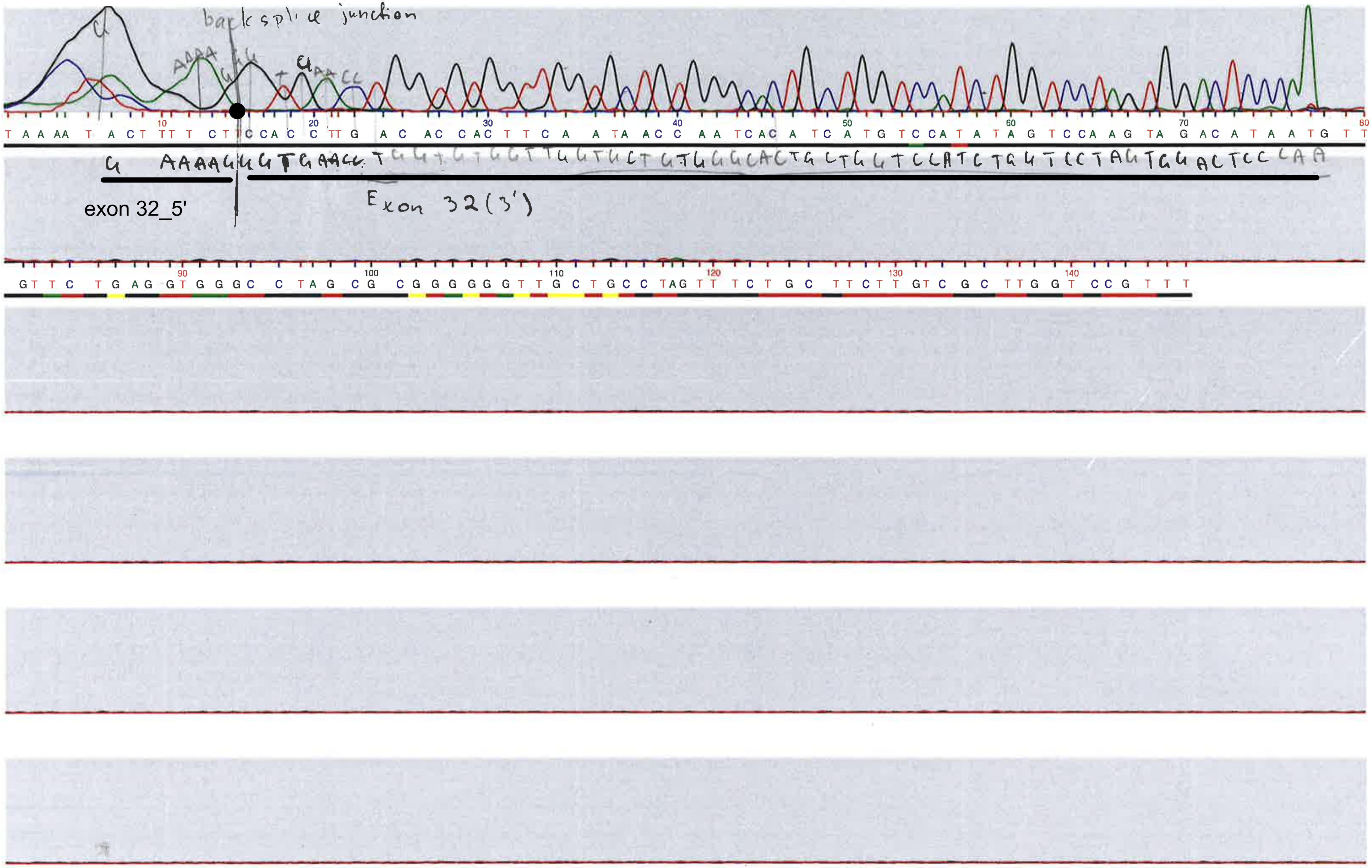

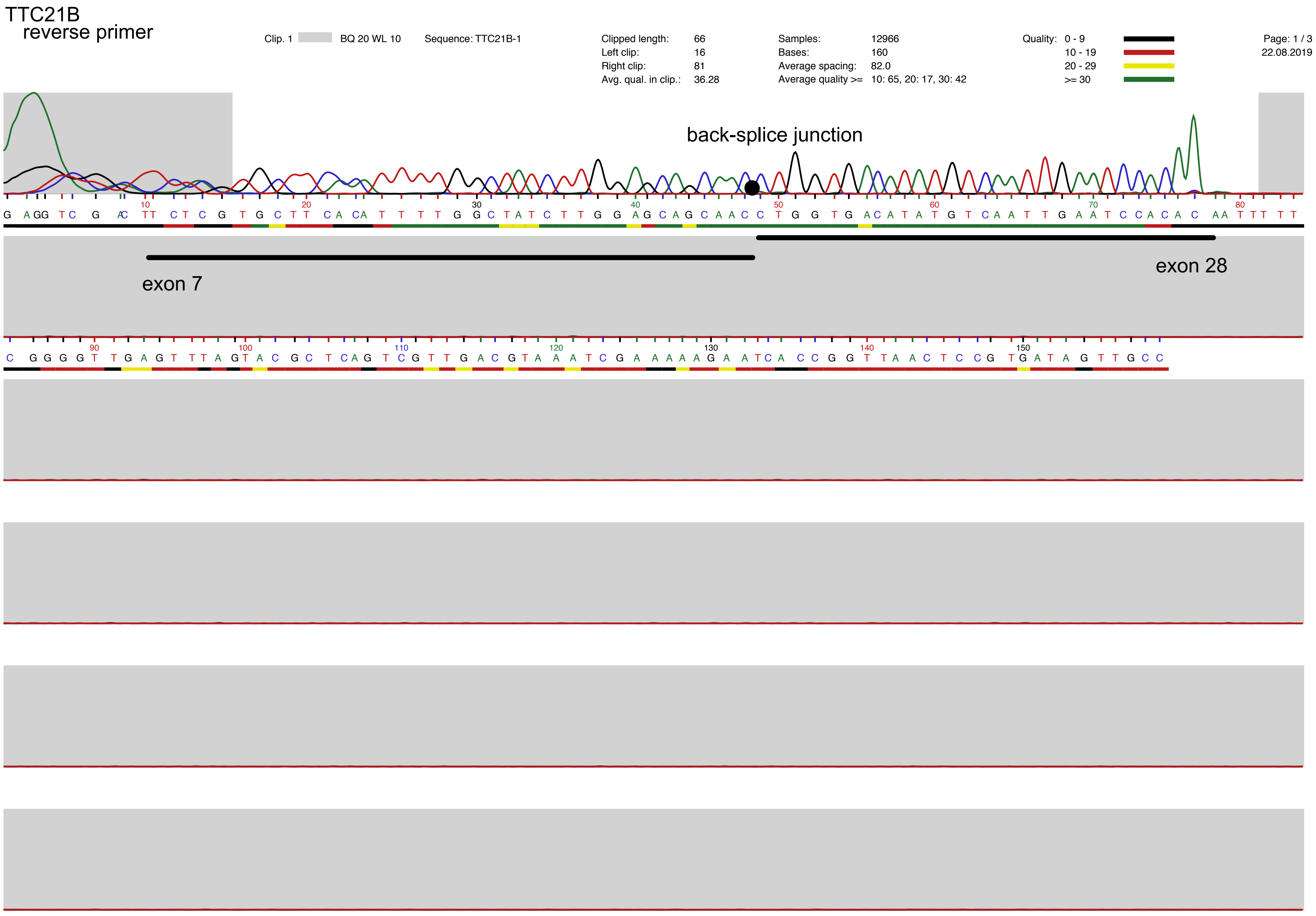
TTC21B
reverse primer
Clip. 1
BQ 20 WL 10
Sequence: TTC21B-1
Clipped length: 66
Left clip: 16
Right clip: 81
Avg. qual. in clip.: 36.28
Samples: 12966
Bases: 160
Average spacing: 82.0
Average quality >= 10: 65, 20: 17, 30: 42
Quality: 0 - 9
10 - 19
20 - 29
>= 30
Page: 1 / 3
22.08.2019
back-splice junction
exon 7
exon 28

Sanger sequencing electropherograms for the 5 circRNA overexpression plasmid constructs, CDYL, BACH1, GLIS1, SMARCA5 and ZKSCAN1 are shown below.

CDYL - overexpression

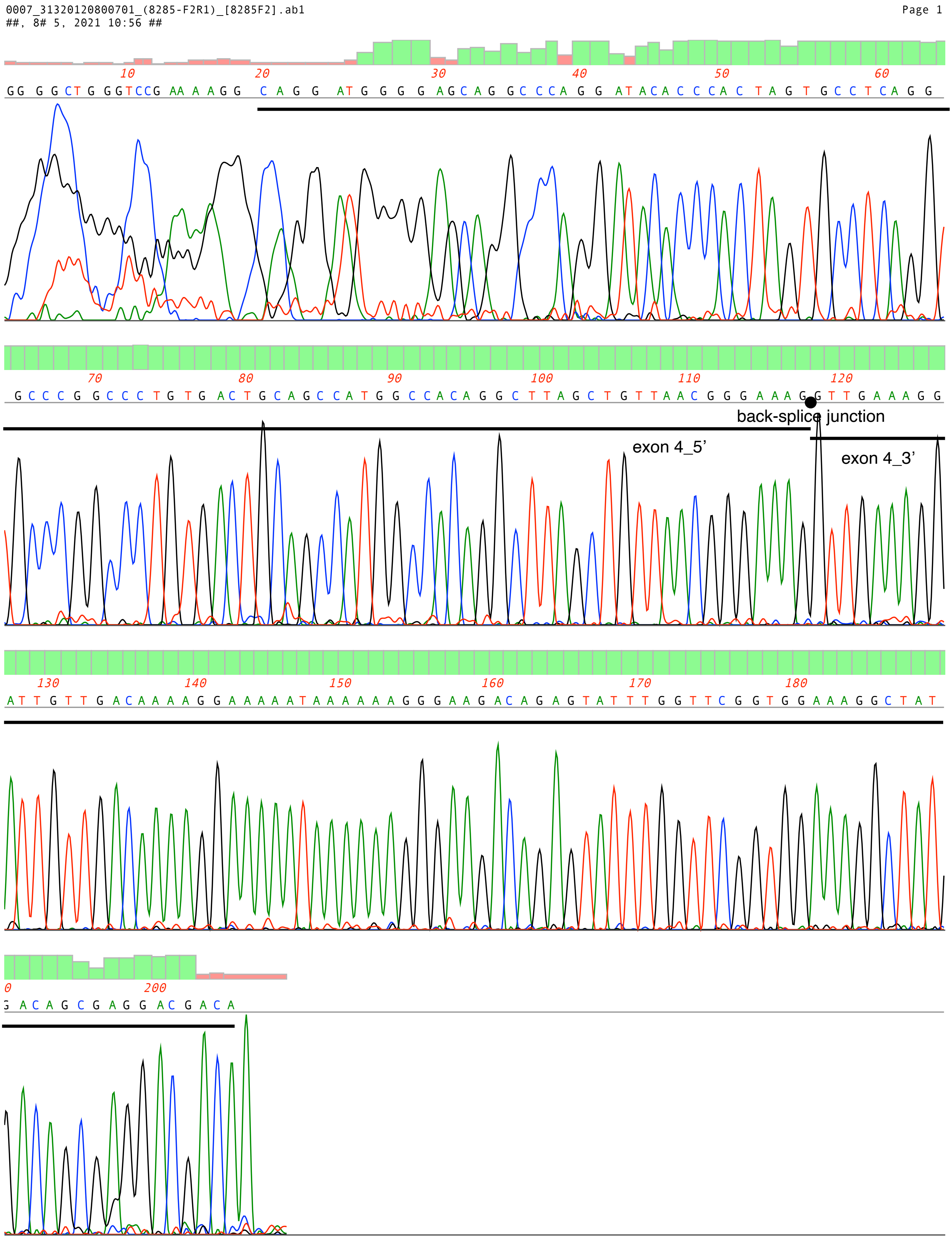

BACH1 - overexpression

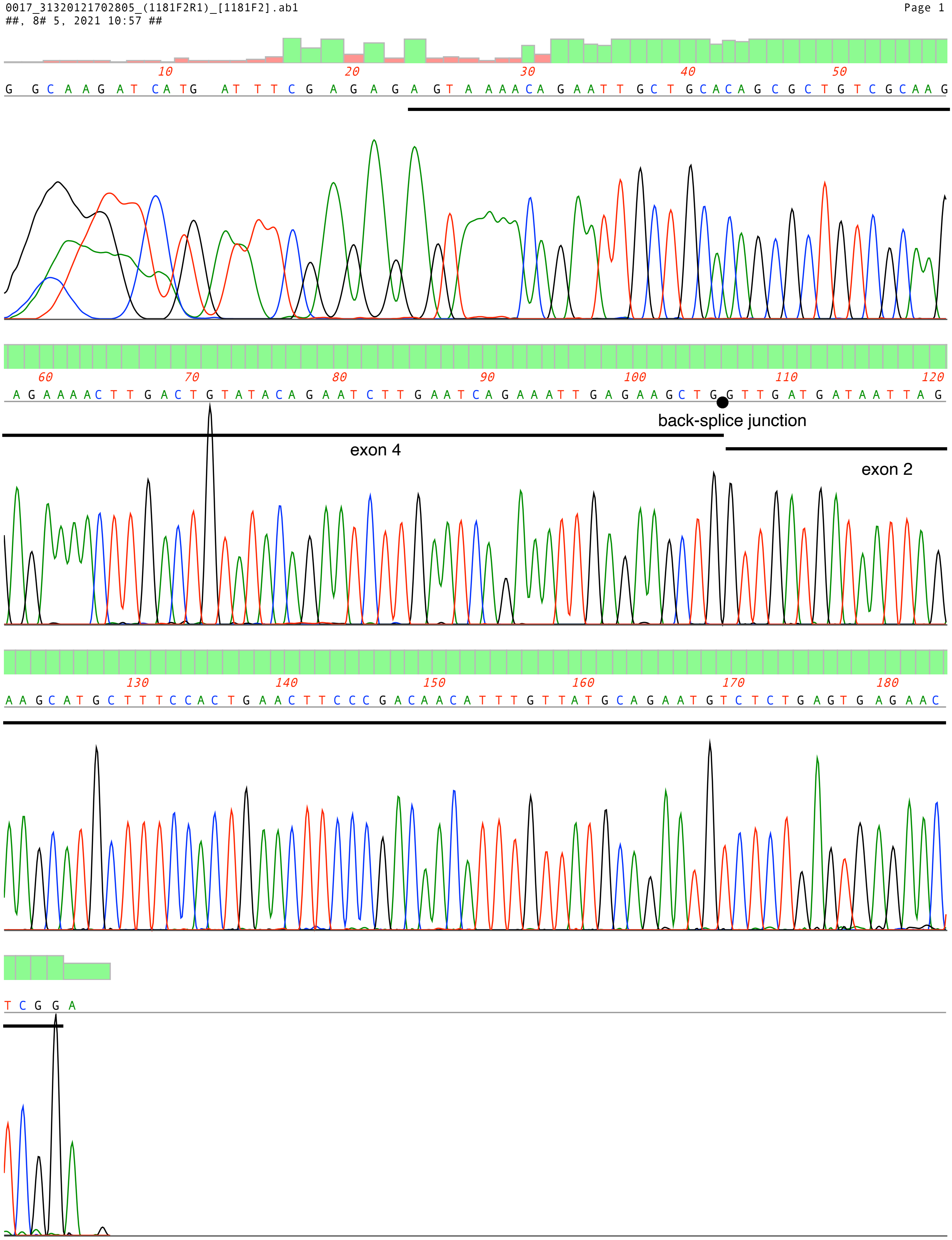

GLIS1 - overexpression

0003_31320122802130_(2079F2R3)_[2079F2].ab1 Page 1
##, 8# 5, 2021 10:56 ##

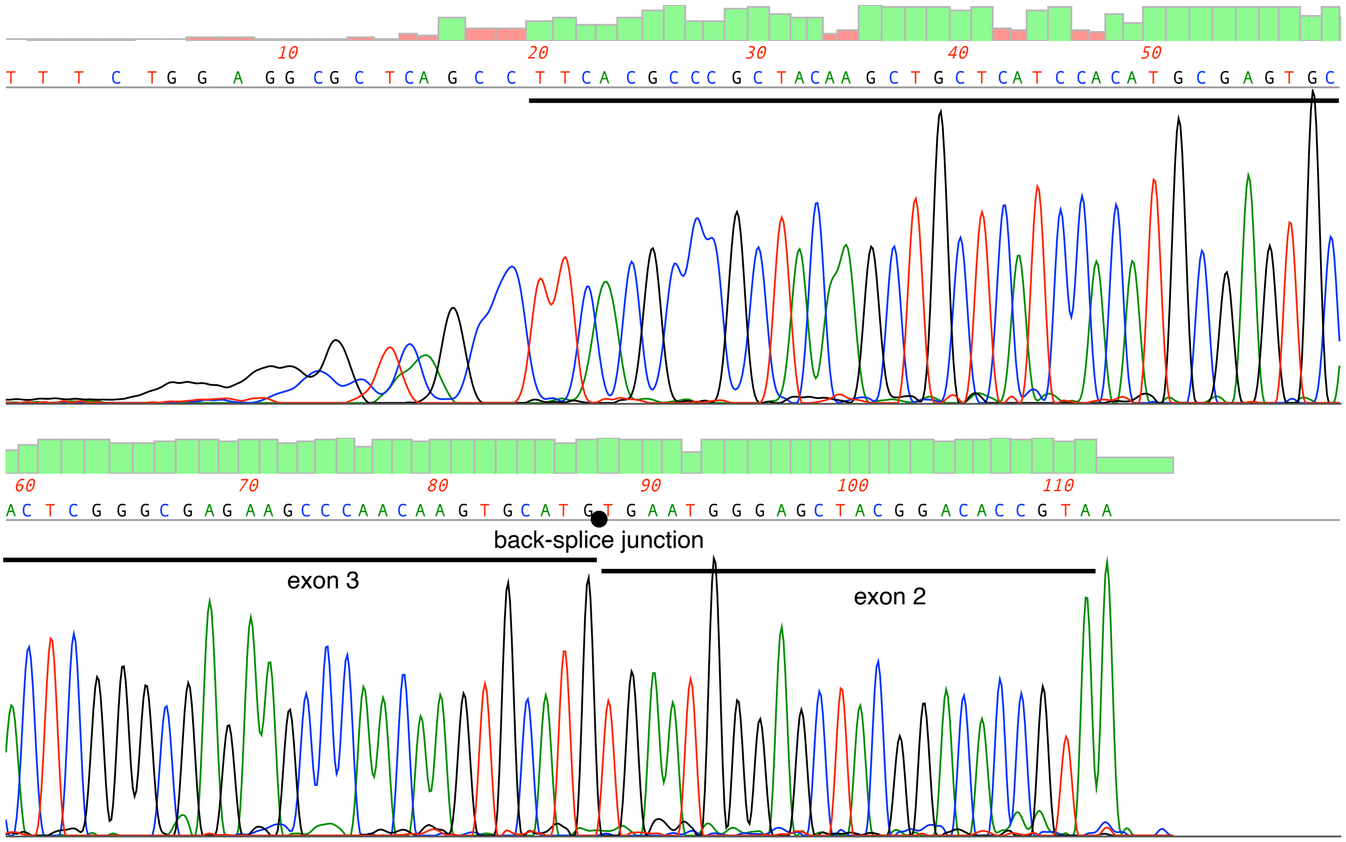

SMARCA5 - overexpression

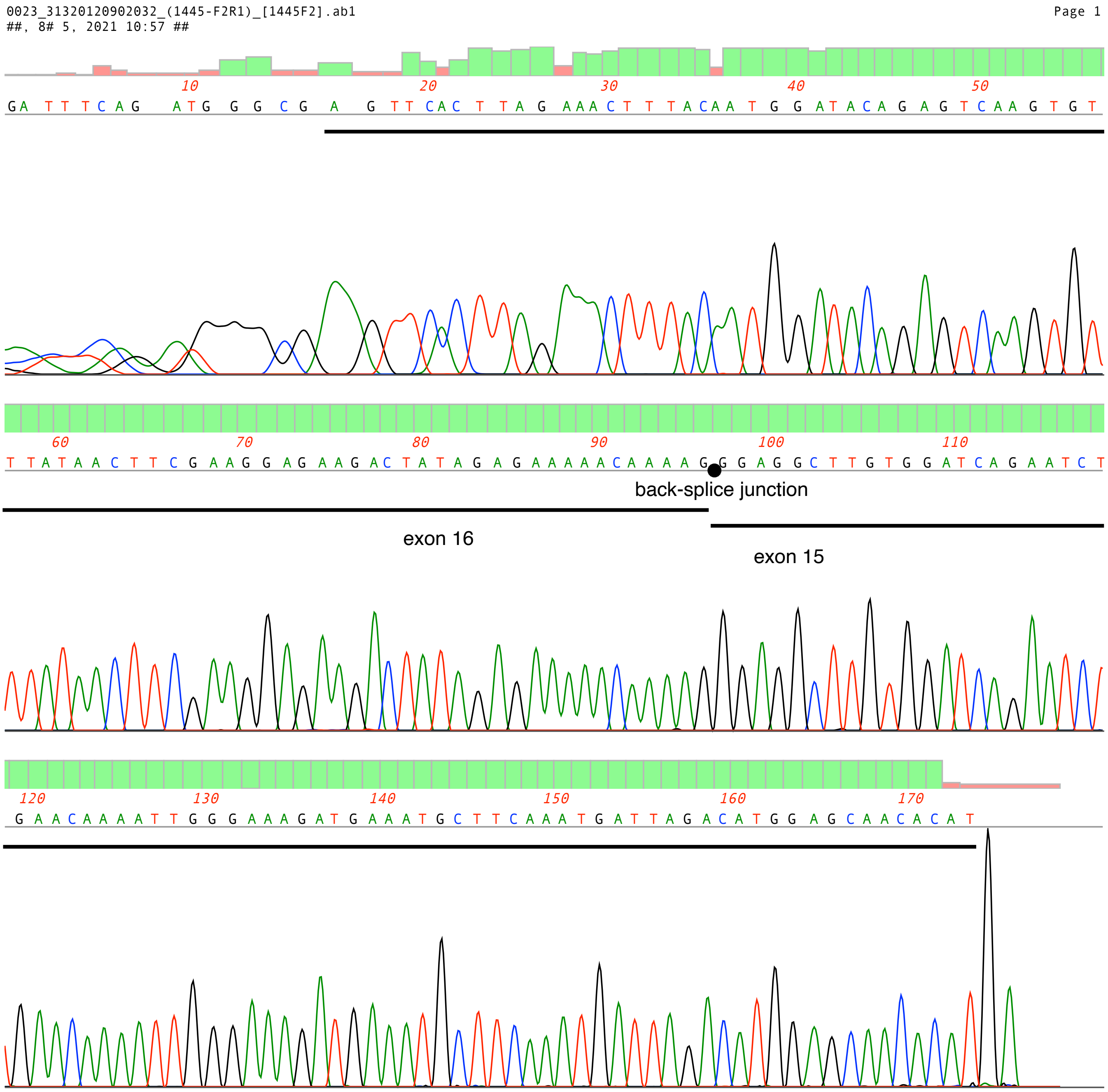

ZKSCAN1- overexpression

0002_31320121702733_(1727-F2R1)_[1727F2].ab1 Page 1
##, 8# 5, 2021 10:56 ##

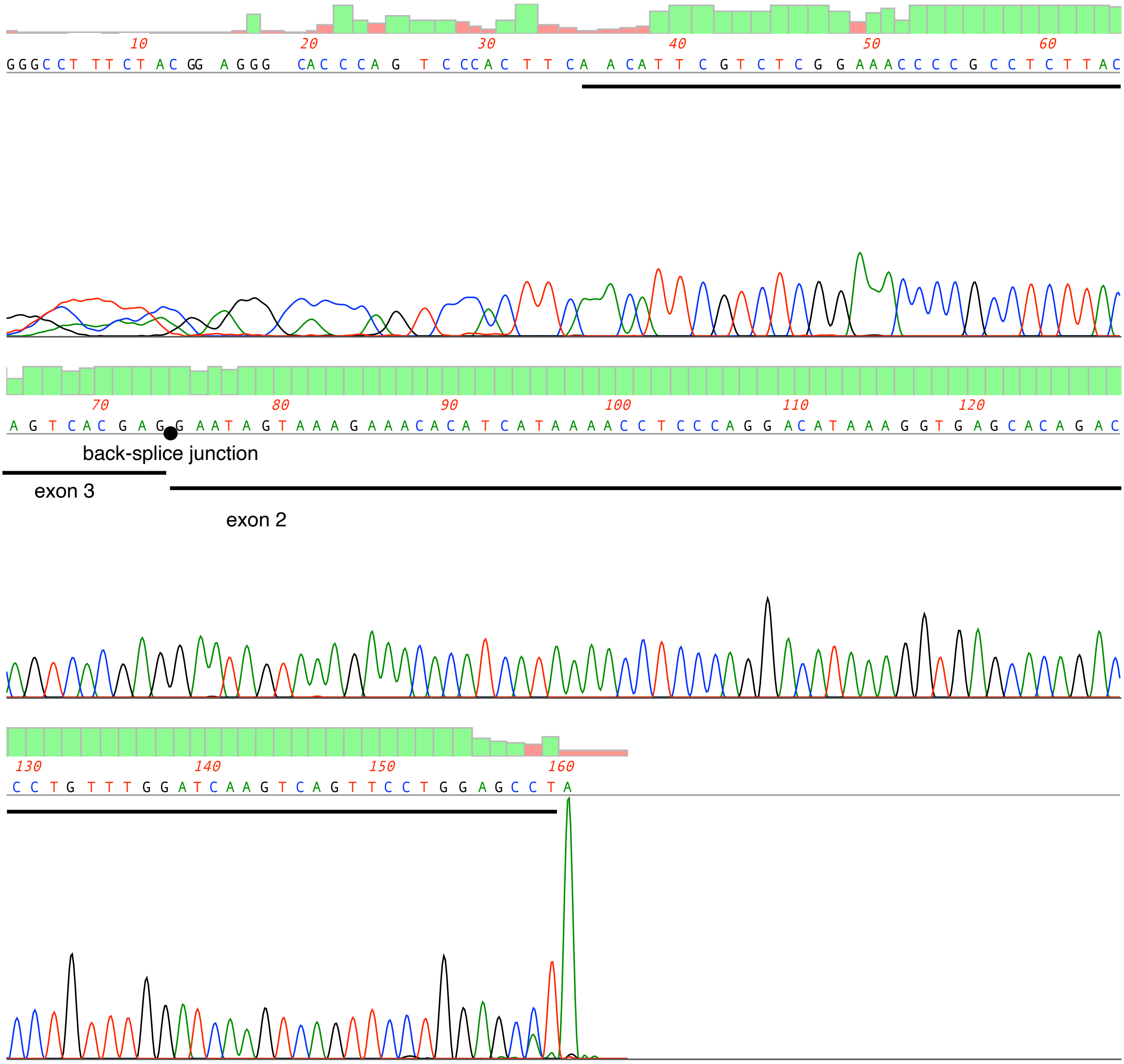

Supplement: Supplementary file 1 [file cancers-13-05138-s001.zip › Supplementary files/S6_Sanger-seq validations.pdf]
