# Supplementary material for: Circular RNAs in Hedgehog Signaling Activation and Hedgehog-Mediated Medulloblastoma Tumors
Source: Cancers (Basel). 2021 Oct 13;13(20):5138. doi: 10.3390/cancers13205138 (PMC8533754; doi:10.3390/cancers13205138)
Supplement: Supplementary file 1 [file cancers-13-05138-s001.zip › Supplementary files/Supplementary figures.pdf]

Figure S1

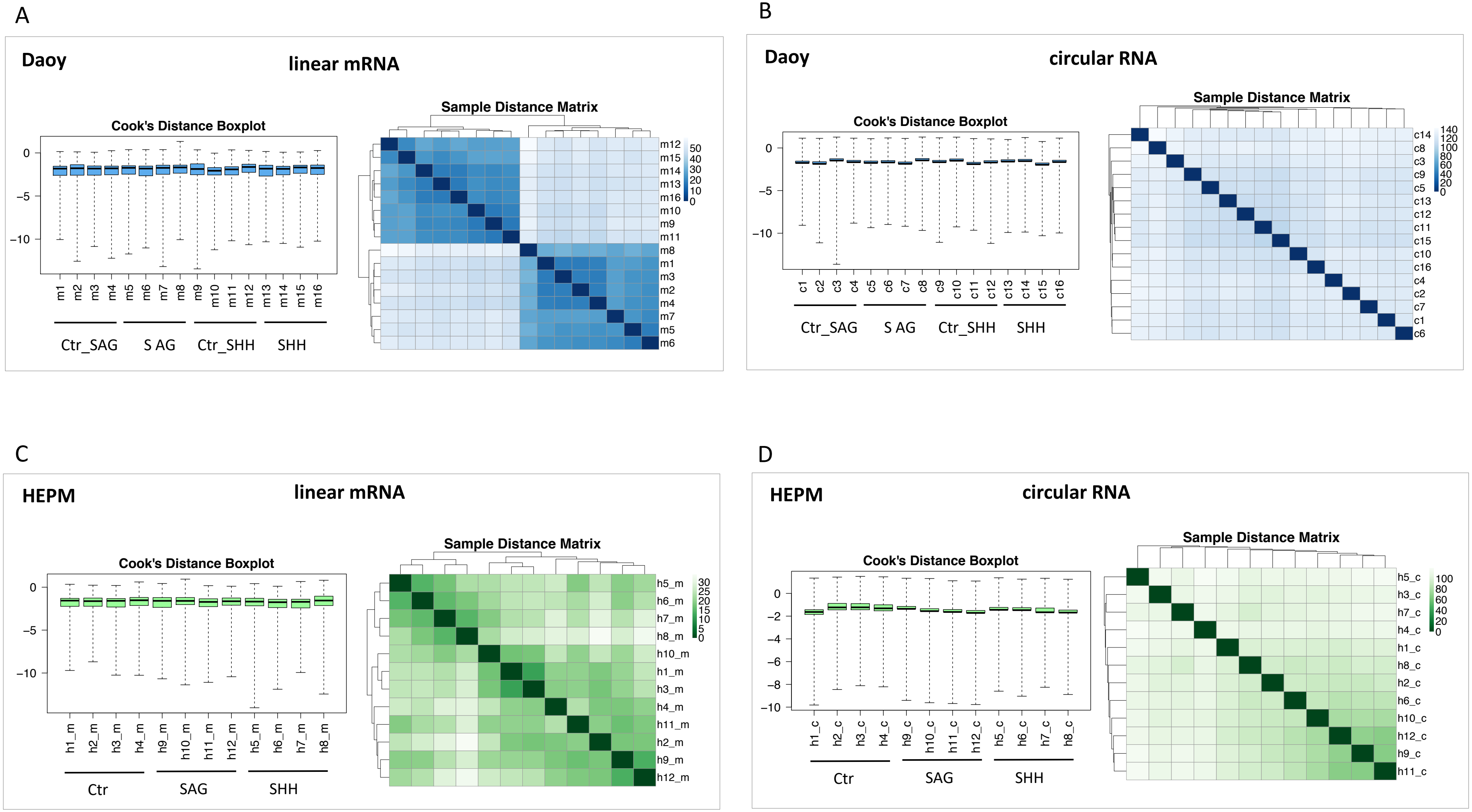

A
Daoy
linear mRNA
Cook's Distance Boxplot
Sample Distance Matrix
0
−5
−10
m1 m2 m3 m4 m5 m6 m7 m8 m9 m10 m11 m12 m13 m14 m15 m16
Ctr_SAG
S AG
Ctr_SHH
SHH
m12 m15 m14 m13 m16 m10 m9 m11 m8 m1 m3 m2 m4 m7 m5 m6
50 40 30 20 10 0
B
Daoy
circular RNA
Cook's Distance Boxplot
Sample Distance Matrix
0
−5
−10
c1 c2 c3 c4 c5 c6 c7 c8 c9 c10 c11 c12 c13 c14 c15 c16
Ctr_SAG
S AG
Ctr_SHH
SHH
c14 c8 c3 c9 c5 c13 c12 c11 c15 c10 c16 c4 c2 c7 c1 c6
140 120 100 80 60 40 20 0
C
HEPM
linear mRNA
Cook's Distance Boxplot
Sample Distance Matrix
0
−5
−10
h1_m h2_m h3_m h4_m h9_m h10_m h11_m h12_m h5_m h6_m h7_m h8_m
Ctr
SAG
SHH
h5_m h6_m h7_m h8_m h10_m h1_m h3_m h4_m h11_m h2_m h9_m h12_m
30 25 20 15 10 5 0
D
HEPM
circular RNA
Cook's Distance Boxplot
Sample Distance Matrix
0
−2
−4
−6
−8
−10
h1_c h2_c h3_c h4_c h9_c h10_c h11_c h12_c h5_c h6_c h7_c h8_c
Ctr
SAG
SHH
h5_c h3_c h7_c h4_c h1_c h8_c h2_c h6_c h10_c h12_c h9_c h11_c
100 80 60 40 20 0

Figure S1

E

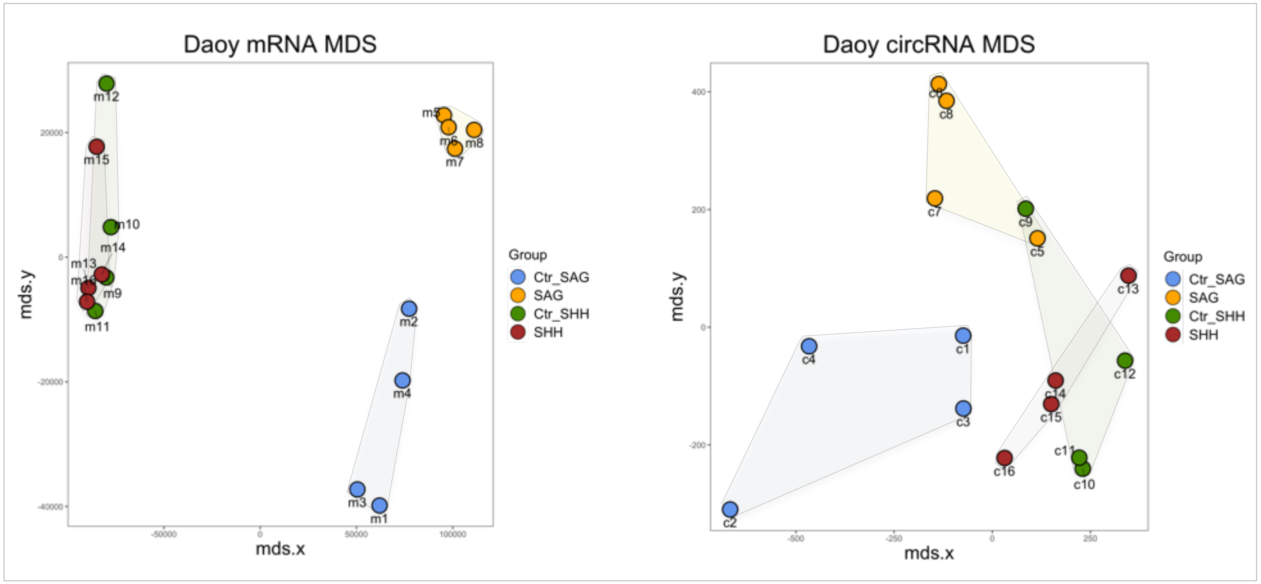
Daoy mRNA MDS
mds.y
mds.x
Group
Ctr_SAG
SAG
Ctr_SHH
SHH
Daoy circRNA MDS
mds.y
mds.x
Group
Ctr_SAG
SAG
Ctr_SHH
SHH

F

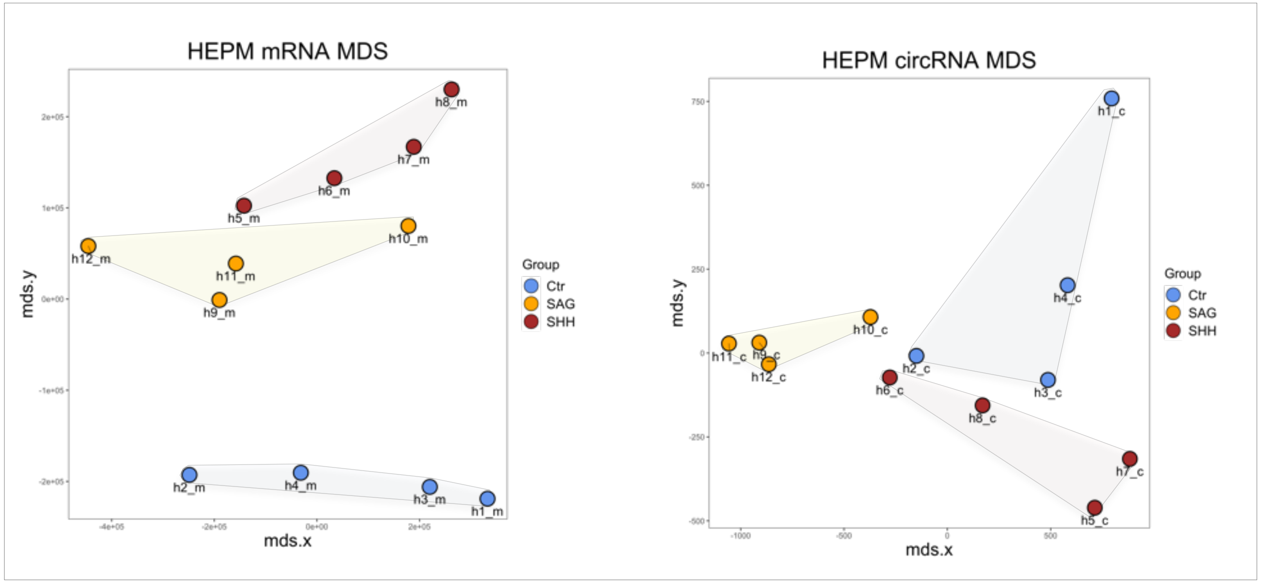
HEPM mRNA MDS
mds.y
mds.x
Group
Ctr
SAG
SHH
HEPM circRNA MDS
mds.y
mds.x
Group
Ctr
SAG
SHH

Supplementary Figure 1. Between-sample variability of DESeq2 normalized RNA-seq data of Daoy and HEPM cells. Related to Figure 1 (A-E).

(**A - D**) (**Left panels in the boxes**) Boxplot highlighting the comparable range of Cook's distances between the samples and the absence of obvious outliers in the RNA-seq data. (**Right panels in the boxes**) Heatmaps of sample distance matrix, depicting the Euclidean distances between the samples. The linear mRNA and circRNA data in Daoy (A, B) and HEPM (C, D) cells are shown. (**E, F**) Kruskal's non-metric multidimensional scaling analysis (MDS) of the count normalized DESeq2 data from the linear mRNA and circRNA fractions of Daoy (E) and HEPM (F) cells, highlighting the overall similarities between the samples in the two-dimensional space.

Figure S2

A

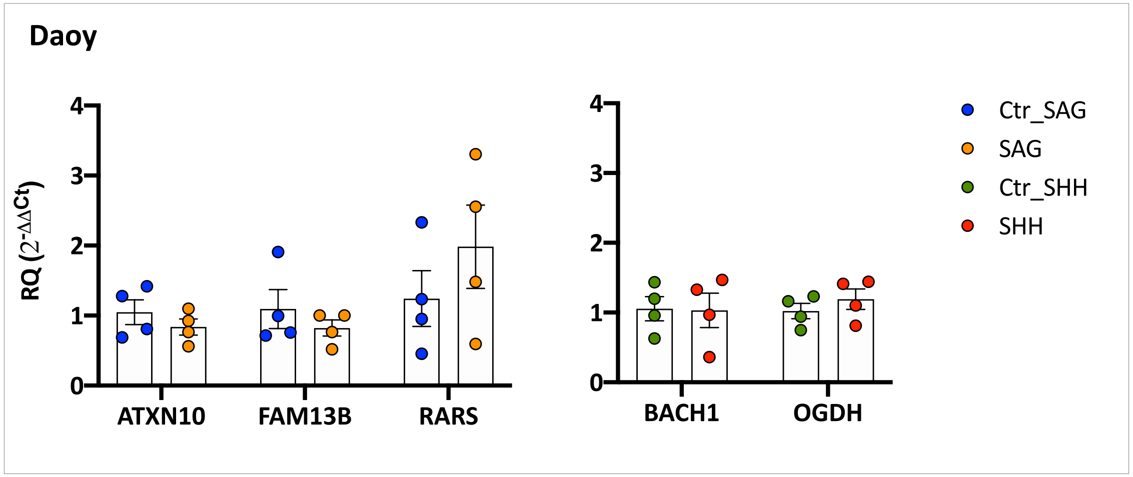
Daoy
RQ ($2^{-\Delta\Delta Ct}$)
0
1
2
3
4
ATXN10
FAM13B
RARS
BACH1
OGDH
Ctr_SAG
SAG
Ctr_SHH
SHH

B

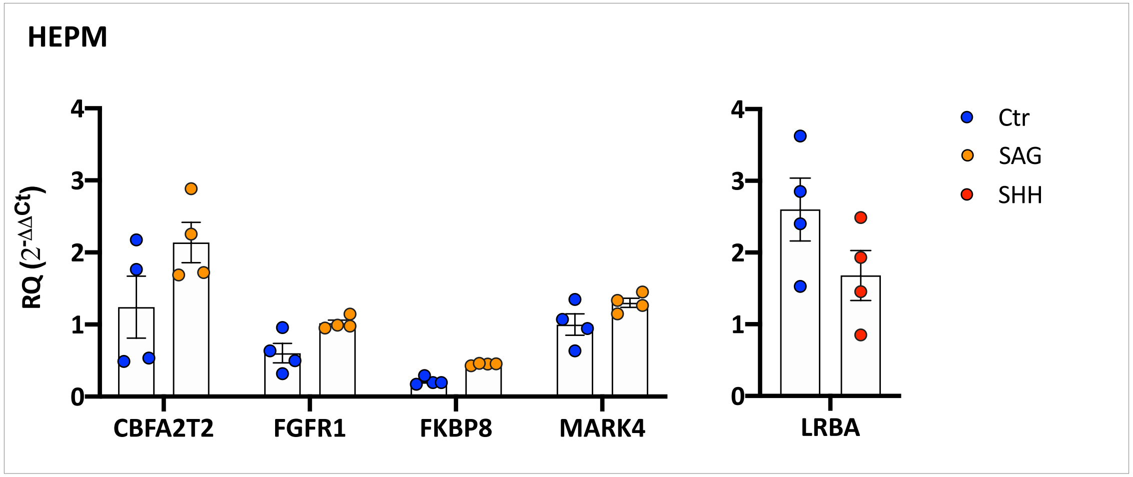
HEPM
RQ ($2^{-\Delta\Delta Ct}$)
0
1
2
3
4
CBFA2T2
FGFR1
FKBP8
MARK4
LRBA
Ctr
SAG
SHH

Supplementary Figure 2. qPCR analysis of the selected DE circRNAs detected in the RNA-seq data analysis by SAG/SHH treatment in Daoy and HEPM cells. Related to Figure 1 (D, E).

(**A, B**) The relative expression ($2^{-\Delta\Delta Ct}$ values) of the selected 5 DE circRNAs in (A) Daoy and 5 DE circRNAs in (B) HEPM cells normalized to the housekeeping gene (TBP) and the corresponding control treatment (DMSO for SAG and PBS for SHH in Daoy cells, DMSO+PBS in HEPM cells) is presented. Error bars indicate the SEM of four independent replicate treatments. RQ denotes the relative quantification of the RNA expression. Note that the same RNA preparations as in the RNA-seq experiments were used. The selected DE RTN4 circRNA could not be PCR amplified and consequently is excluded from this analysis.

Figure S3

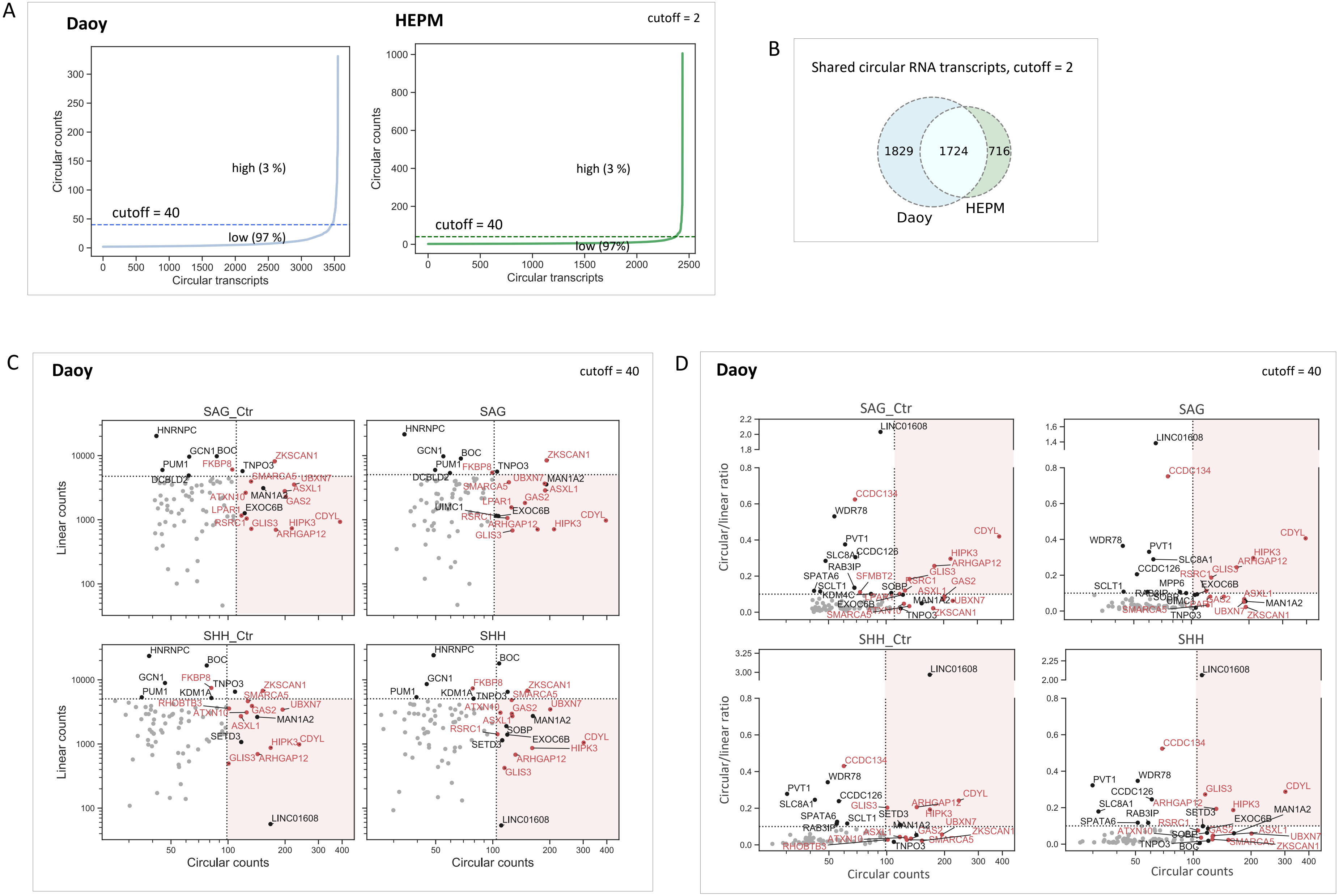
A
Daoy
HEPM
cutoff = 2
Circular counts
Circular transcripts
high (3 %)
low (97 %)
cutoff = 40
low (97%)
B
Shared circular RNA transcripts, cutoff = 2
1829
1724
716
Daoy
HEPM
C
Daoy
cutoff = 40
SAG_Ctr
SAG
SHH_Ctr
SHH
Linear counts
Circular counts
D
Daoy
cutoff = 40
SAG_Ctr
SAG
SHH_Ctr
SHH
Circular/linear ratio
Circular counts

Figure S3

E

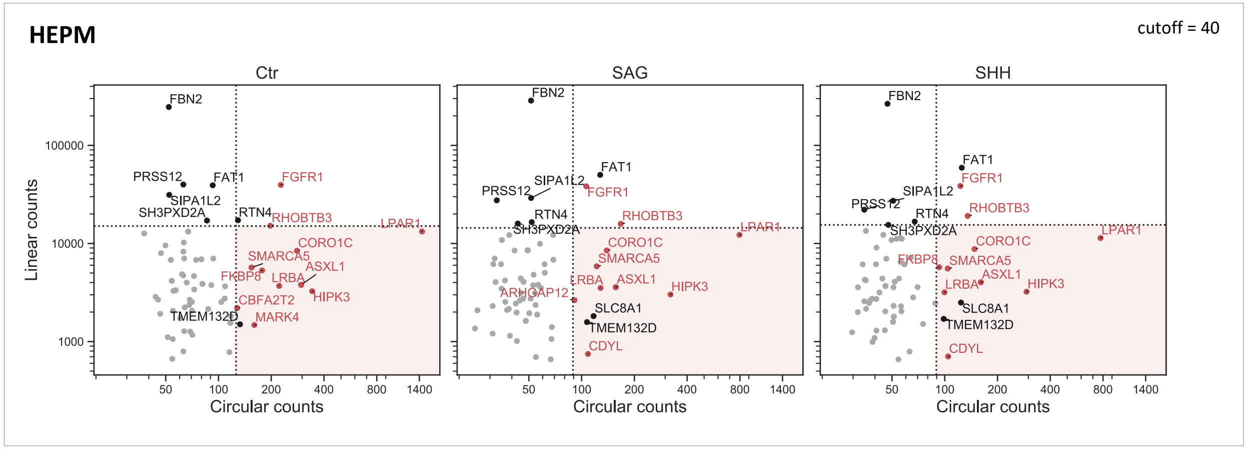
HEPM
cutoff = 40
Ctr
SAG
SHH
Linear counts
Circular counts
FBN2
PRSS12
FAT1
FGFR1
SIPA1L2
SH3PXD2A
RTN4
RHOBTB3
LPAR1
CORO1C
SMARCA5
ASXL1
FKBP8
LRBA
HIPK3
CBFA2T2
TMEM132D
MARK4
ARHGAP12
SLC8A1
CDYL

F

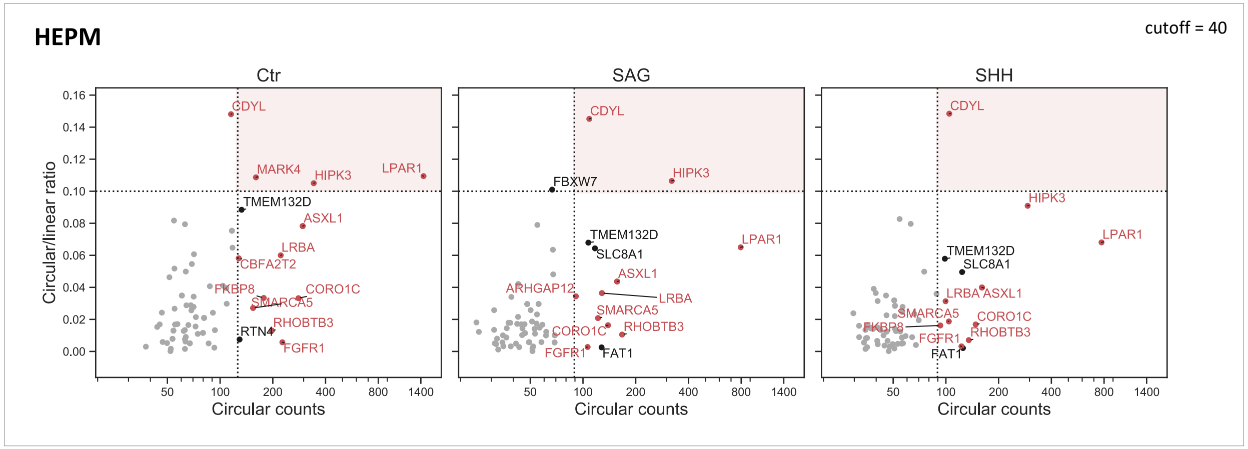
HEPM
cutoff = 40
Ctr
SAG
SHH
Circular/linear ratio
Circular counts
CDYL
MARK4
HIPK3
LPAR1
TMEM132D
ASXL1
LRBA
CBFA2T2
FKBP8
CORO1C
SMARCA5
RHOBTB3
RTN4
FGFR1
FBXW7
SLC8A1
ARHGAP12
FAT1

Supplementary Figure 3. Circular to linear ratios of lowly and highly expressed circRNAs in Daoy and HEPM cells. Related to Figure 2.

(**A**) circRNA abundance in Daoy and HEPM cells. circRNA transcripts (x axis) are plotted by ascending order according to their abundance (y axis), determined as mean reads of DESeq2 normalized RNA-seq data across all 16 (Daoy) and 12 (HEPM) samples. A mean read cutoff = 2 is implemented. Only a small portion of circRNAs (≈ 3%) in both cell types are relatively highly expressed (defined as higher than a mean read cutoff = 40).

(B) Venn diagram representing the overlap of circRNAs expression in Daoy and HEPM cells at cutoff = 2.

(C - F) Highly expressed circRNAs (mean read cutoff = 40) plotted against corresponding linear mRNA from the same host gene or circular/linear ratios in (C, D) Daoy and (E, F) HEPM cells. (C, E) Annotated are the circles within the upper 80th percentile circular and the upper 90th percentile linear reads scores for each treatment group (SAG or SHH) and the respective controls (SAG_Ctr or SHH_Ctr in Daoy and Ctr in HEPM). circRNAs within the red box are both above the 80th percentile of circular reads and below the 90th percentile of linear reads. (D, F) Annotated are the circles above the 80th percentile of circular reads or with circular/linear ratio > 0.1. circRNAs within the red box meet both criteria. Selected genes (Table 1) are highlighted in red.

Figure S4

A

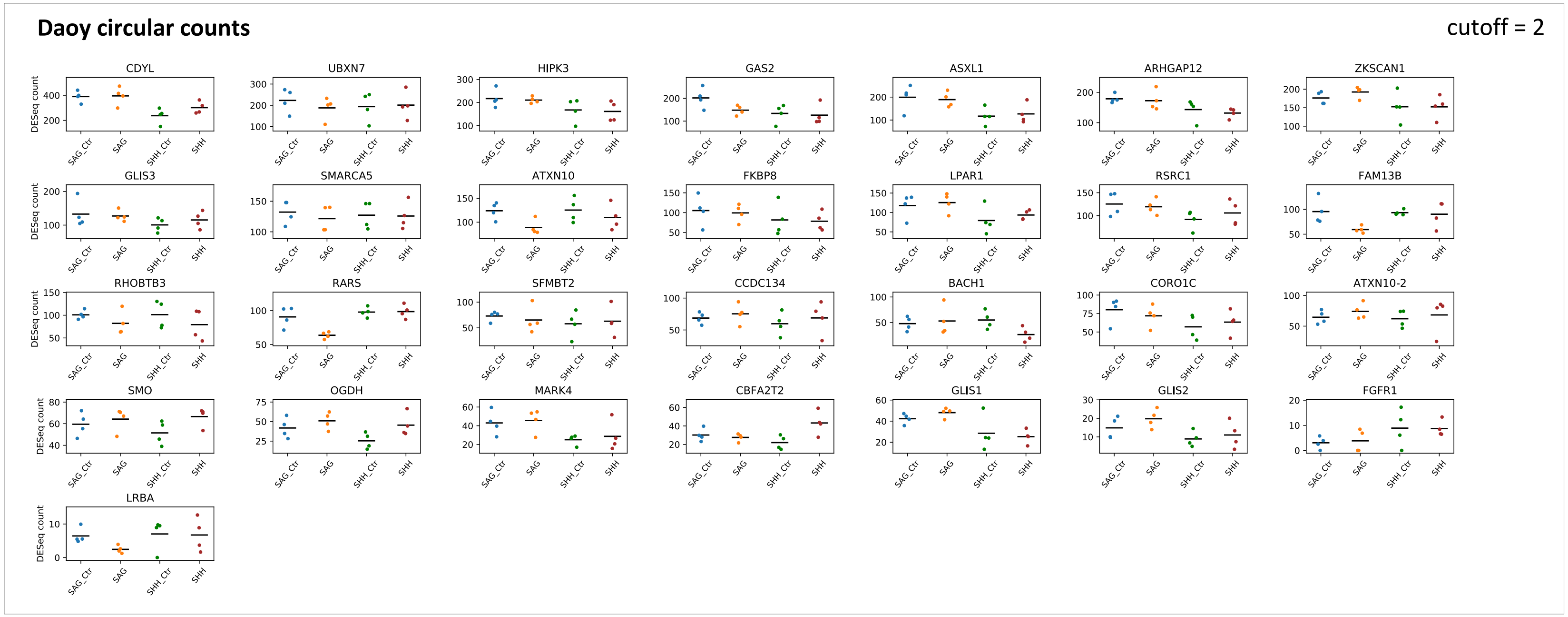
Daoy circular counts
cutoff = 2
DESeq count
CDYL
UBXN7
HIPK3
GAS2
ASXL1
ARHGAP12
ZKSCAN1
GLIS3
SMARCA5
ATXN10
FKBP8
LPAR1
RSRC1
FAM13B
RHOBTB3
RARS
SFMBT2
CCDC134
BACH1
CORO1C
ATXN10-2
SMO
OGDH
MARK4
CBFA2T2
GLIS1
GLIS2
FGFR1
LRBA
SAG_Ctr
SAG
SHH_Ctr
SHH

B

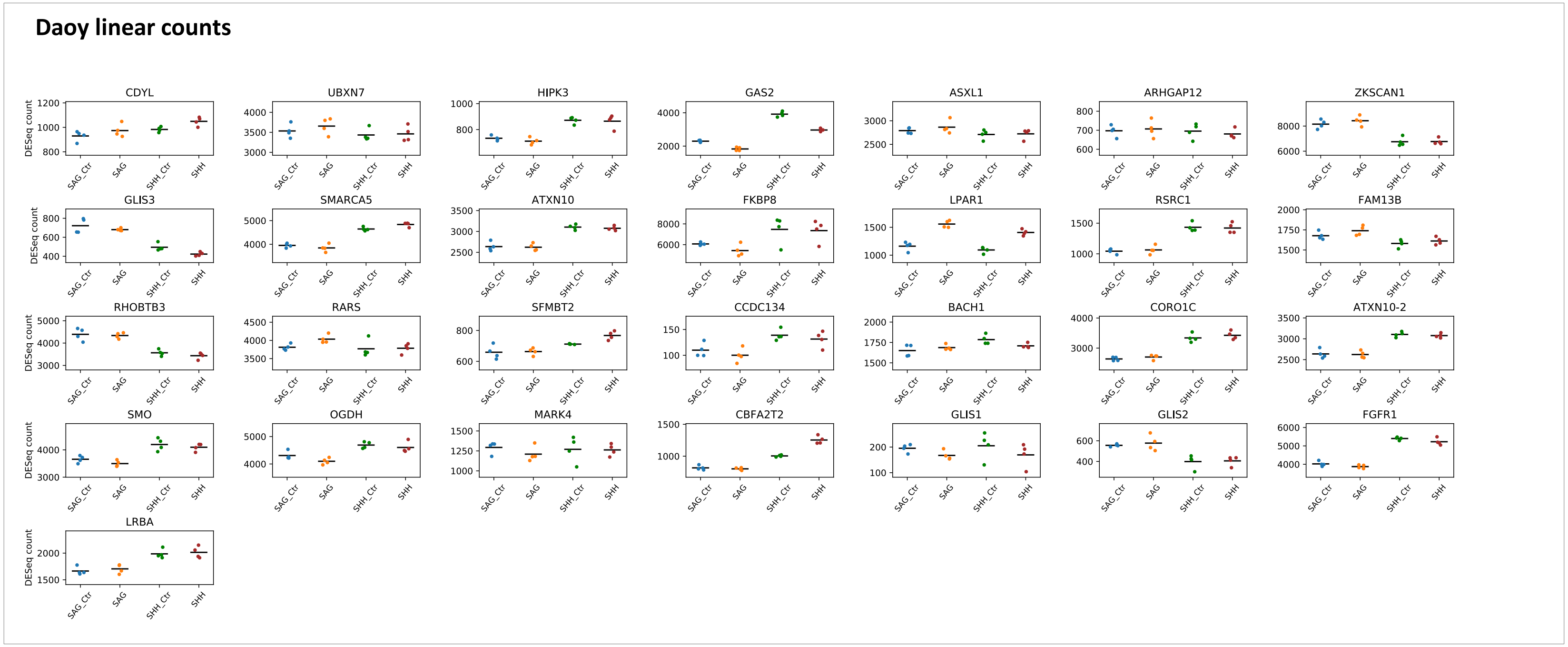
Daoy linear counts
DESeq count
CDYL
UBXN7
HIPK3
GAS2
ASXL1
ARHGAP12
ZKSCAN1
GLIS3
SMARCA5
ATXN10
FKBP8
LPAR1
RSRC1
FAM13B
RHOBTB3
RARS
SFMBT2
CCDC134
BACH1
CORO1C
ATXN10-2
SMO
OGDH
MARK4
CBFA2T2
GLIS1
GLIS2
FGFR1
LRBA
SAG_Ctr
SAG
SHH_Ctr
SHH

Figure S4

C

HEPM circular counts

cutoff = 2

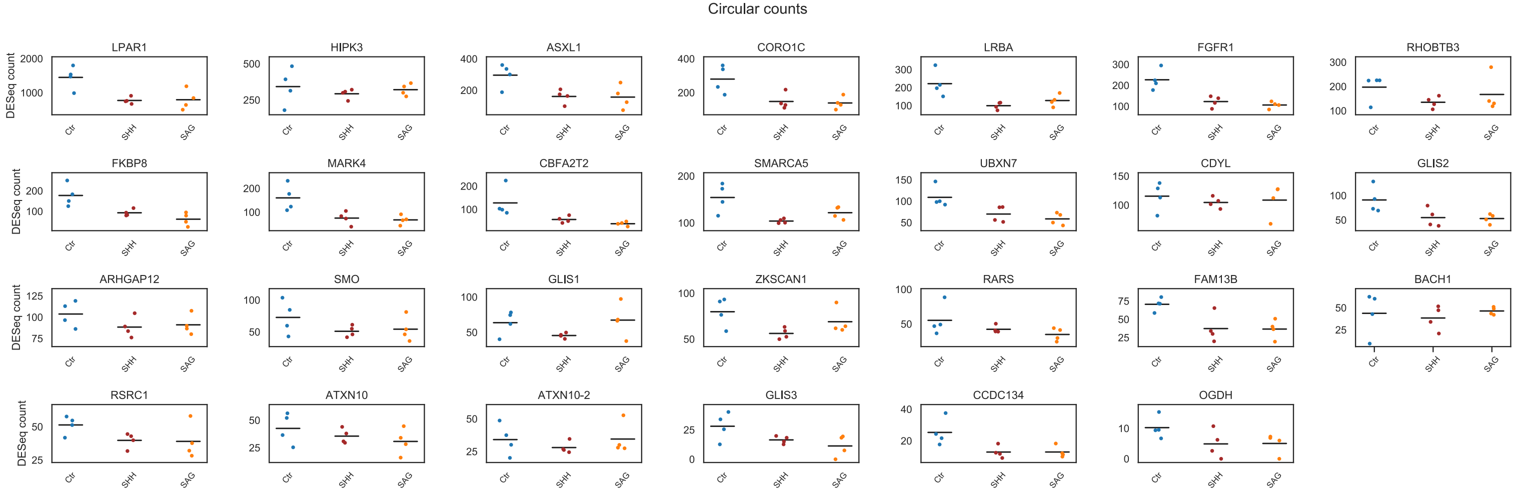

D

HEPM linear counts

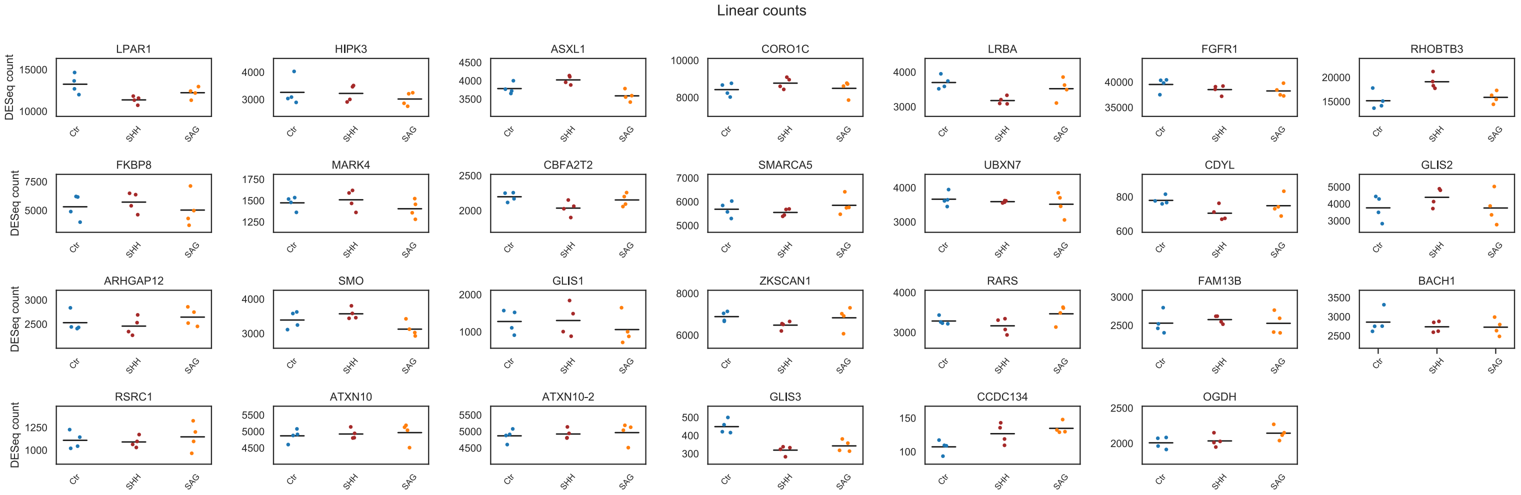

Supplementary Figure 4. Expression of RNA circles based on DESeq2 normalized RNA-seq counts by SAG/SHH treatment of Daoy and HEPM cells. Related to Table 1, Figure 1 (D, E) and Figure 2 (A, B).

The RNA expression in Daoy (**A, B**) and HEPM (**C, D**) cells of the selected genes (Table 1), with a mean read count for circRNAs above 2, is presented. Note, that both the expression of circRNA (A, C) and the respective linear mRNAs (B, D) is shown. In Daoy all 29 circRNAs are included, but in HEPM only 27 circRNAs, as GAS2 and SFMBT2 have < 2 mean read counts.

Figure S5

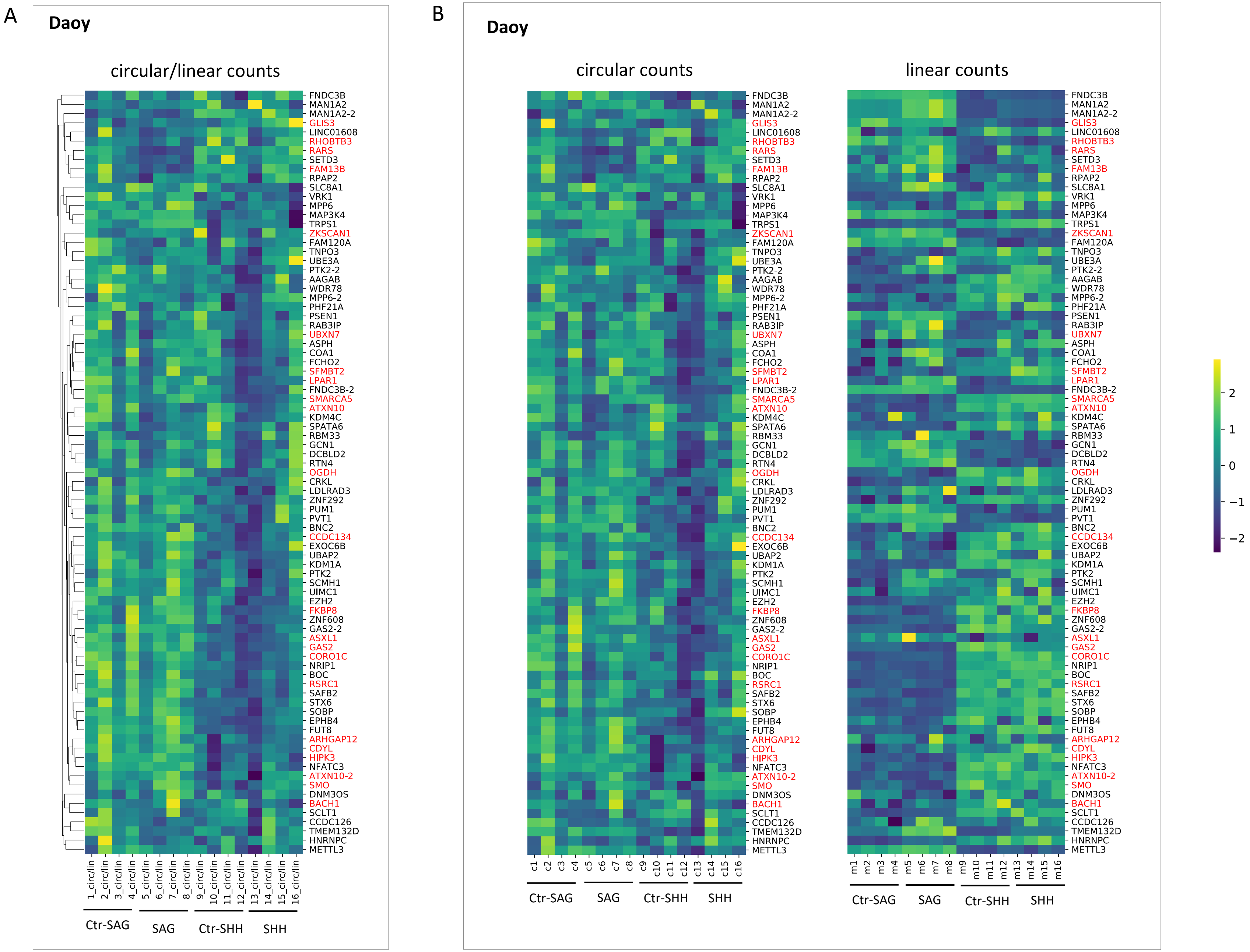
A
Daoy
circular/linear counts
B
Daoy
circular counts
linear counts
FNDC3B
MAN1A2
MAN1A2-2
GLIS3
LINC01608
RHOBTB3
RARS
SETD3
FAM13B
RPAP2
SLC8A1
VRK1
MPP6
MAP3K4
TRPS1
ZKSCAN1
FAM120A
TNPO3
UBE3A
PTK2-2
AAGAB
WDR78
MPP6-2
PHF21A
PSEN1
RAB3IP
UBXN7
ASPH
COA1
FCHO2
SFMBT2
LPAR1
FNDC3B-2
SMARCA5
ATXN10
KDM4C
SPATA6
RBM33
GCN1
DCBLD2
RTN4
OGDH
CRKL
LDLRAD3
ZNF292
PUM1
PVT1
BNC2
CCDC134
EXOC6B
UBAP2
KDM1A
PTK2
SCMH1
UIMC1
EZH2
FKBP8
ZNF608
GAS2-2
ASXL1
GAS2
CORO1C
NRIP1
BOC
RSRC1
SAFB2
STX6
SOBP
EPHB4
FUT8
ARHGAP12
CDYL
HIPK3
NFATC3
ATXN10-2
SMO
DNM3OS
BACH1
SCLT1
CCDC126
TMEM132D
HNRNPC
METTL3
1_circ/lin 2_circ/lin 3_circ/lin 4_circ/lin 5_circ/lin 6_circ/lin 7_circ/lin 8_circ/lin 9_circ/lin 10_circ/lin 11_circ/lin 12_circ/lin 13_circ/lin 14_circ/lin 15_circ/lin 16_circ/lin
c1 c2 c3 c4 c5 c6 c7 c8 c9 c10 c11 c12 c13 c14 c15 c16
m1 m2 m3 m4 m5 m6 m7 m8 m9 m10 m11 m12 m13 m14 m15 m16
Ctr-SAG
SAG
Ctr-SHH
SHH
2
1
0
−1
−2

Figure S5

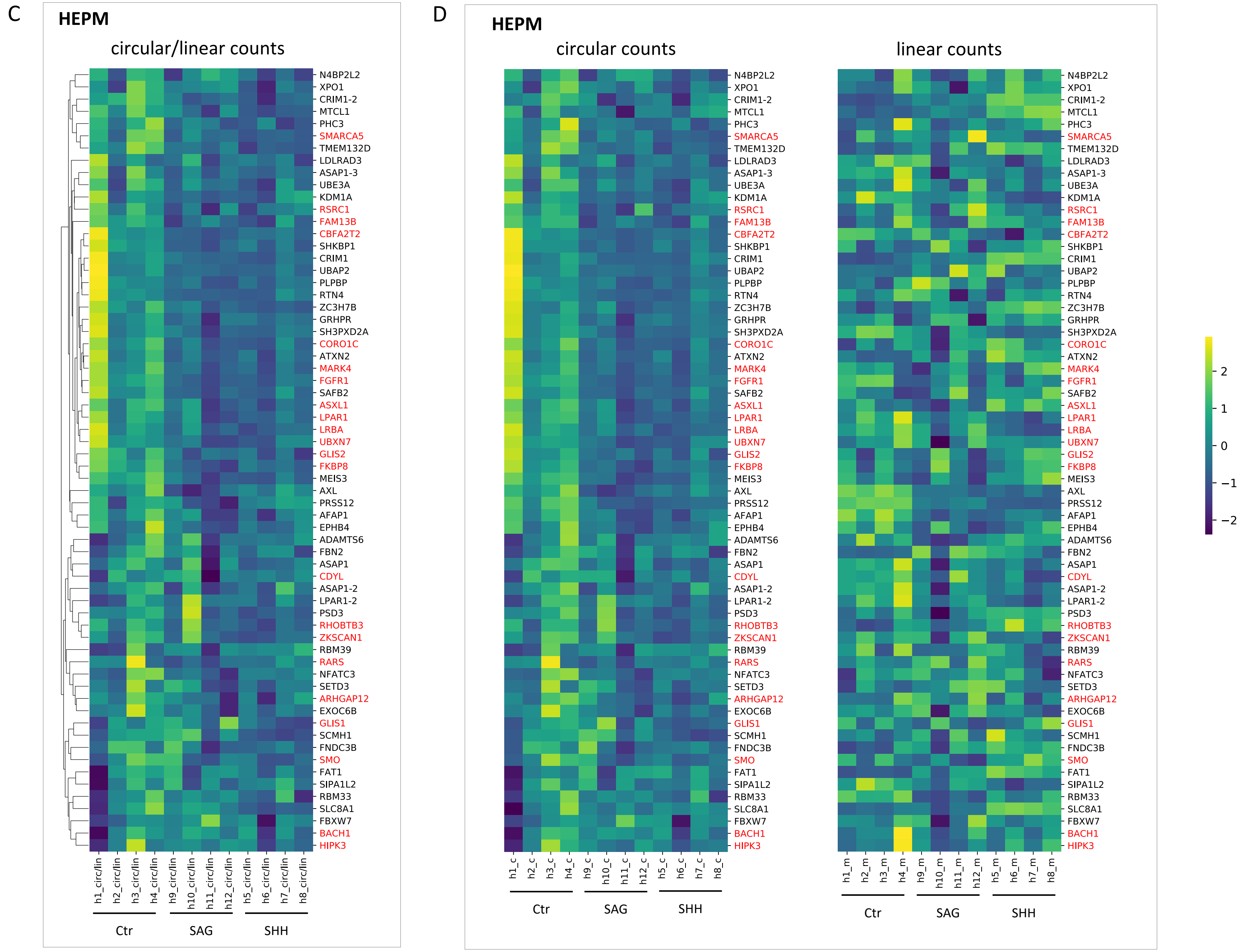
C
HEPM
circular/linear counts
N4BP2L2
XPO1
CRIM1-2
MTCL1
PHC3
SMARCA5
TMEM132D
LDLRAD3
ASAP1-3
UBE3A
KDM1A
RSRC1
FAM13B
CBFA2T2
SHKBP1
CRIM1
UBAP2
PLPBP
RTN4
ZC3H7B
GRHPR
SH3PXD2A
CORO1C
ATXN2
MARK4
FGFR1
SAFB2
ASXL1
LPAR1
LRBA
UBXN7
GLIS2
FKBP8
MEIS3
AXL
PRSS12
AFAP1
EPHB4
ADAMTS6
FBN2
ASAP1
CDYL
ASAP1-2
LPAR1-2
PSD3
RHOBTB3
ZKSCAN1
RBM39
RARS
NFATC3
SETD3
ARHGAP12
EXOC6B
GLIS1
SCMH1
FNDC3B
SMO
FAT1
SIPA1L2
RBM33
SLC8A1
FBXW7
BACH1
HIPK3
h1_circ/lin
h2_circ/lin
h3_circ/lin
h4_circ/lin
h9_circ/lin
h10_circ/lin
h11_circ/lin
h12_circ/lin
h5_circ/lin
h6_circ/lin
h7_circ/lin
h8_circ/lin
Ctr
SAG
SHH
D
HEPM
circular counts
linear counts
N4BP2L2
XPO1
CRIM1-2
MTCL1
PHC3
SMARCA5
TMEM132D
LDLRAD3
ASAP1-3
UBE3A
KDM1A
RSRC1
FAM13B
CBFA2T2
SHKBP1
CRIM1
UBAP2
PLPBP
RTN4
ZC3H7B
GRHPR
SH3PXD2A
CORO1C
ATXN2
MARK4
FGFR1
SAFB2
ASXL1
LPAR1
LRBA
UBXN7
GLIS2
FKBP8
MEIS3
AXL
PRSS12
AFAP1
EPHB4
ADAMTS6
FBN2
ASAP1
CDYL
ASAP1-2
LPAR1-2
PSD3
RHOBTB3
ZKSCAN1
RBM39
RARS
NFATC3
SETD3
ARHGAP12
EXOC6B
GLIS1
SCMH1
FNDC3B
SMO
FAT1
SIPA1L2
RBM33
SLC8A1
FBXW7
BACH1
HIPK3
h1_c
h2_c
h3_c
h4_c
h9_c
h10_c
h11_c
h12_c
h5_c
h6_c
h7_c
h8_c
Ctr
SAG
SHH
h1_m
h2_m
h3_m
h4_m
h9_m
h10_m
h11_m
h12_m
h5_m
h6_m
h7_m
h8_m
Ctr
SAG
SHH
2
1
0
−1
−2

Supplementary Figure 5. Circular to linear RNA ratios of individual samples from Control, SAG and SHH treatment groups in Daoy and HEPM cells. Related to Figure 2 (F, G)

Heatmaps with hierarchical clustering in (**A, B**) Daoy and (**C, D**) HEPM cells based on (A, C) circular/linear ratios and (B, D) corresponding circular and linear reads of highly expressed circRNAs (cutoff = 40). Each treated or untreated sample is presented as a column. Shown in each row are the normalized z-scores (see Materials and Methods) for each gene. Yellow indicates higher z scores, while blue lower. Note, that gene clustering patterns based on circular counts are quite similar to the gene clustering based on circular/linear ratios, while linear counts cluster quite differently from that of circular/linear or circular counts. Selected genes (Table 1) are highlighted in red.

Figure S6

A

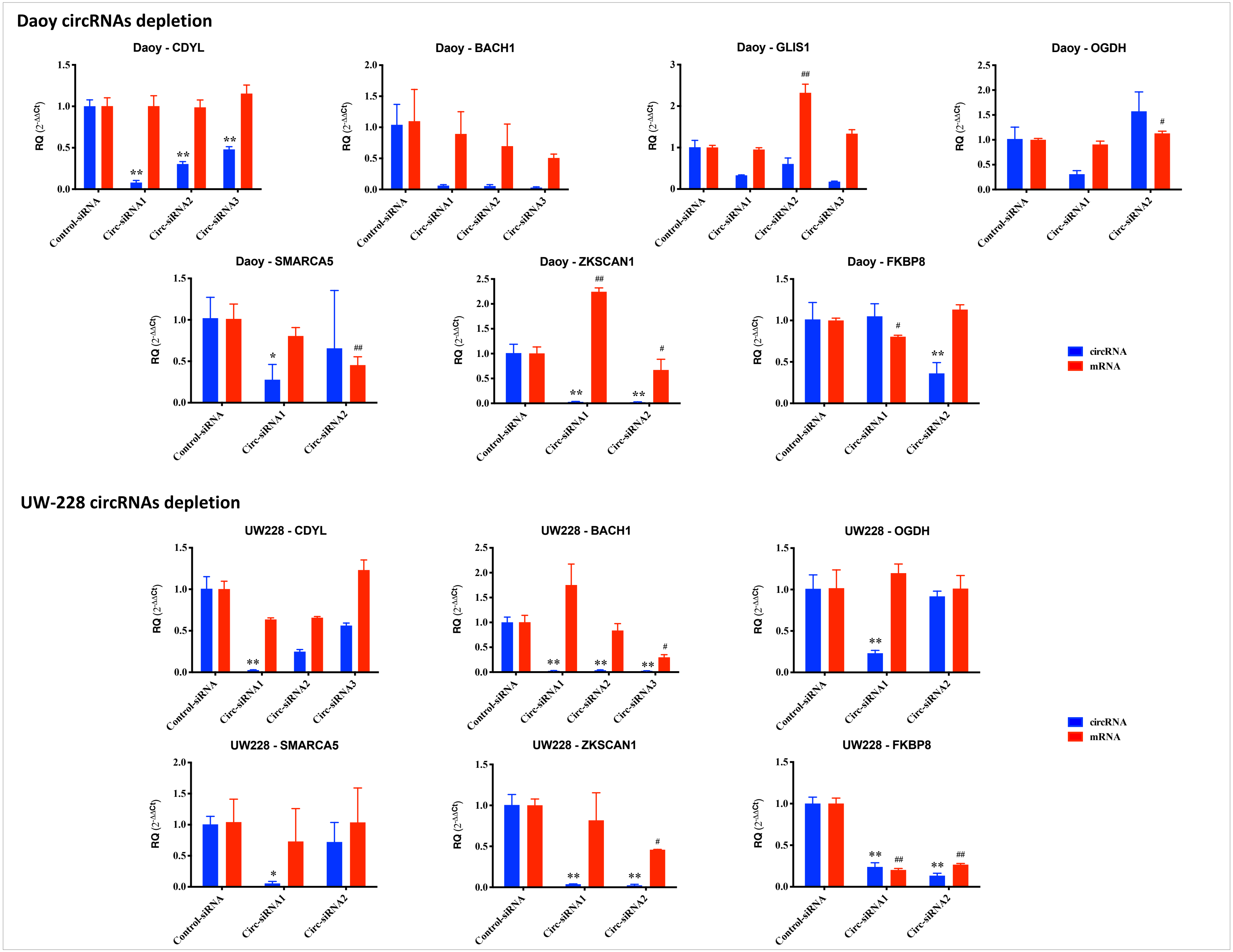
Daoy circRNAs depletion
Daoy - CDYL
Daoy - BACH1
Daoy - GLIS1
Daoy - OGDH
Daoy - SMARCA5
Daoy - ZKSCAN1
Daoy - FKBP8
RQ ($2^{-\Delta\Delta Ct}$)
Control-siRNA
Circ-siRNA1
Circ-siRNA2
Circ-siRNA3
circRNA
mRNA
UW-228 circRNAs depletion
UW228 - CDYL
UW228 - BACH1
UW228 - OGDH
UW228 - SMARCA5
UW228 - ZKSCAN1
UW228 - FKBP8

Figure S6

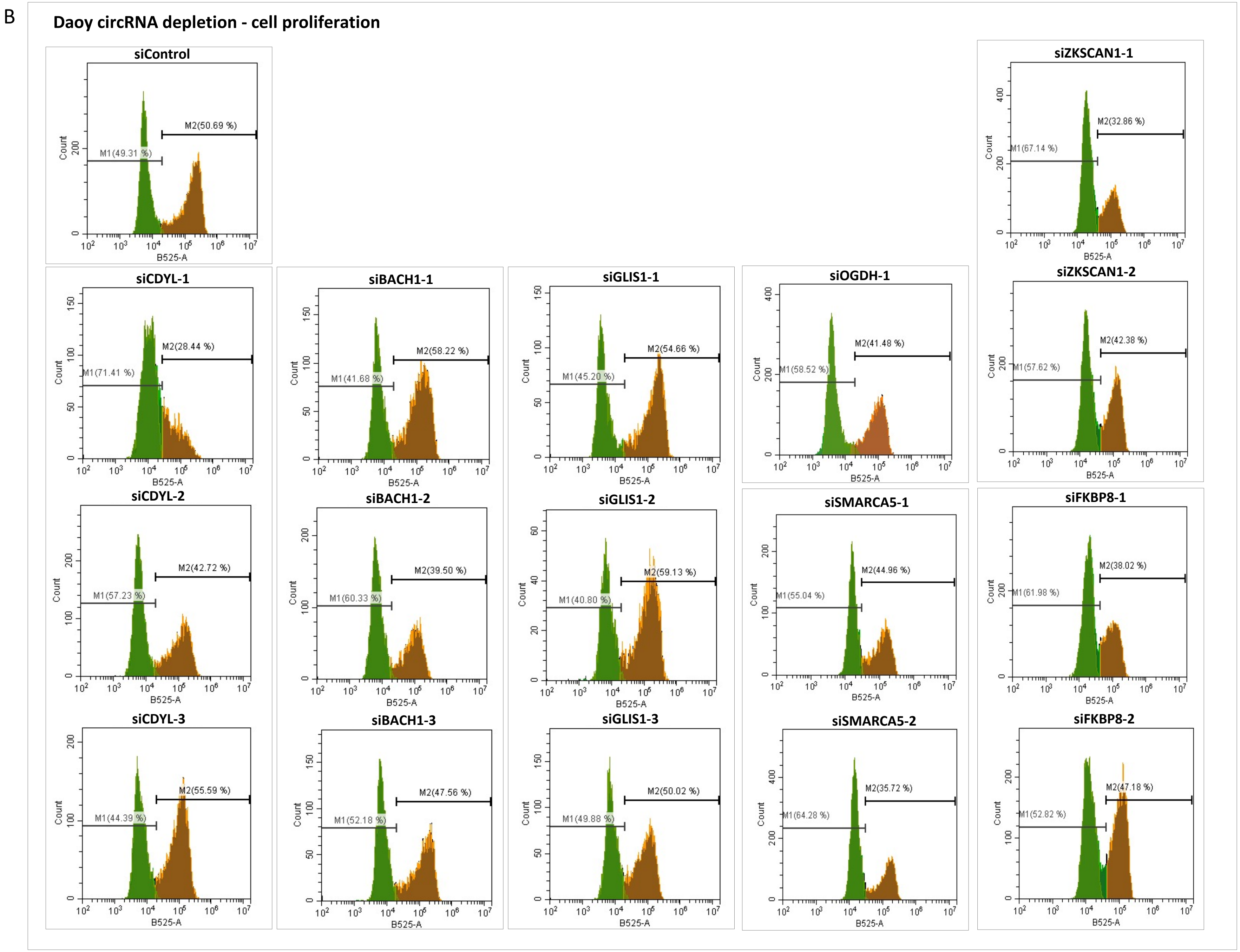
B
Daoy circRNA depletion - cell proliferation
siControl
M1(49.31 %)
M2(50.69 %)
siZKSCAN1-1
M1(67.14 %)
M2(32.86 %)
siZKSCAN1-2
M1(57.62 %)
M2(42.38 %)
siCDYL-1
M1(71.41 %)
M2(28.44 %)
siBACH1-1
M1(41.68 %)
M2(58.22 %)
siGLIS1-1
M1(45.20 %)
M2(54.66 %)
siOGDH-1
M1(58.52 %)
M2(41.48 %)
siCDYL-2
M1(57.23 %)
M2(42.72 %)
siBACH1-2
M1(60.33 %)
M2(39.50 %)
siGLIS1-2
M1(40.80 %)
M2(59.13 %)
siSMARCA5-1
M1(55.04 %)
M2(44.96 %)
siFKBP8-1
M1(61.98 %)
M2(38.02 %)
siCDYL-3
M1(44.39 %)
M2(55.59 %)
siBACH1-3
M1(52.18 %)
M2(47.56 %)
siGLIS1-3
M1(49.88 %)
M2(50.02 %)
siSMARCA5-2
M1(64.28 %)
M2(35.72 %)
siFKBP8-2
M1(52.82 %)
M2(47.18 %)
Count
B525-A

Figure S6

C

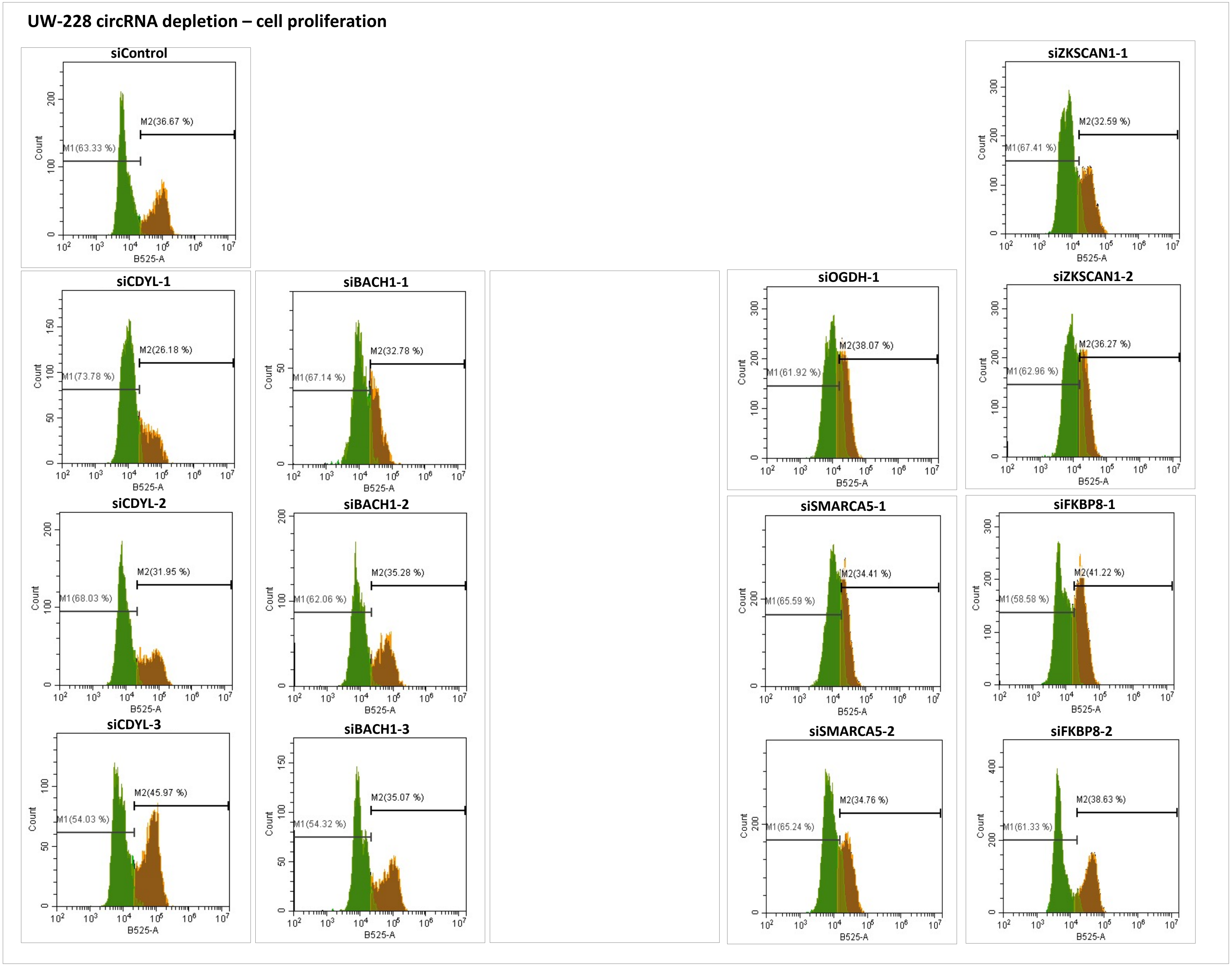

UW-228 circRNA depletion – cell proliferation
siControl
M1(63.33 %)
M2(36.67 %)
siZKSCAN1-1
M1(67.41 %)
M2(32.59 %)
siCDYL-1
M1(73.78 %)
M2(26.18 %)
siBACH1-1
M1(67.14 %)
M2(32.78 %)
siOGDH-1
M1(61.92 %)
M2(38.07 %)
siZKSCAN1-2
M1(62.96 %)
M2(36.27 %)
siCDYL-2
M1(68.03 %)
M2(31.95 %)
siBACH1-2
M1(62.06 %)
M2(35.28 %)
siSMARCA5-1
M1(65.59 %)
M2(34.41 %)
siFKBP8-1
M1(58.58 %)
M2(41.22 %)
siCDYL-3
M1(54.03 %)
M2(45.97 %)
siBACH1-3
M1(54.32 %)
M2(35.07 %)
siSMARCA5-2
M1(65.24 %)
M2(34.76 %)
siFKBP8-2
M1(61.33 %)
M2(38.63 %)
Count
B525-A

Figure S6

D

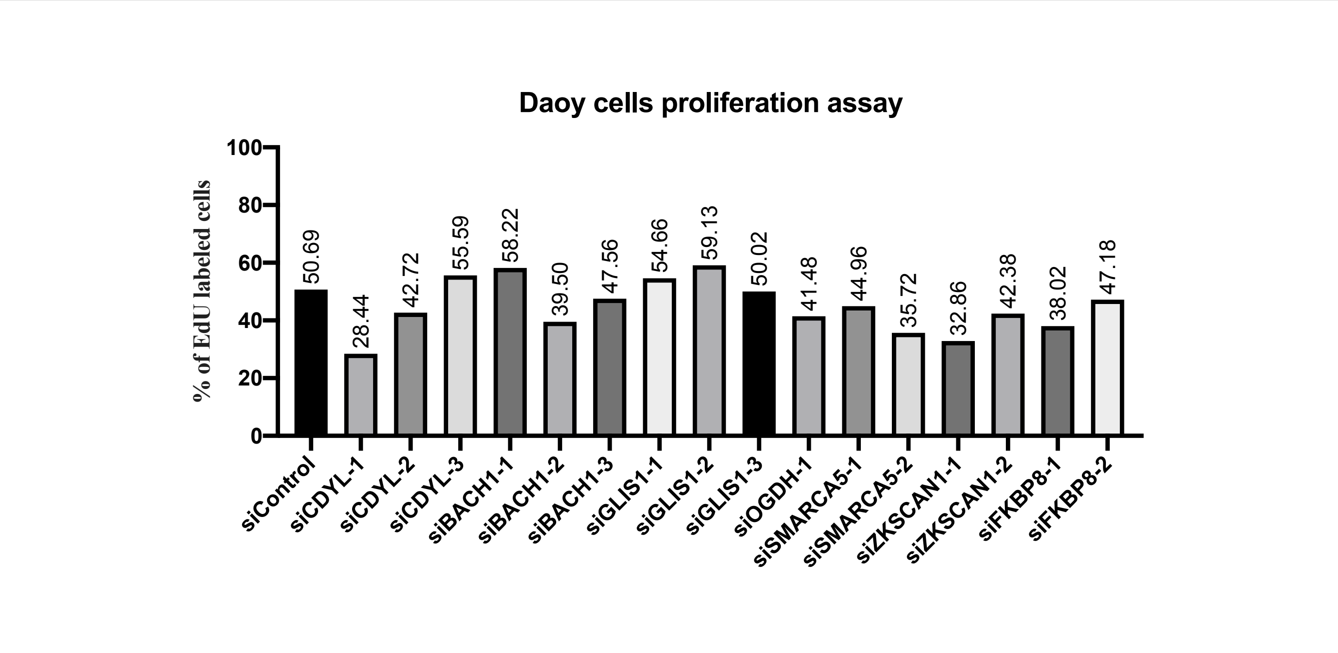
Daoy cells proliferation assay
% of EdU labeled cells
100
80
60
40
20
0
50.69
28.44
42.72
55.59
58.22
39.50
47.56
54.66
59.13
50.02
41.48
44.96
35.72
32.86
42.38
38.02
47.18
siControl
siCDYL-1
siCDYL-2
siCDYL-3
siBACH1-1
siBACH1-2
siBACH1-3
siGLIS1-1
siGLIS1-2
siGLIS1-3
siOGDH-1
siSMARCA5-1
siSMARCA5-2
siZKSCAN1-1
siZKSCAN1-2
siFKBP8-1
siFKBP8-2

E

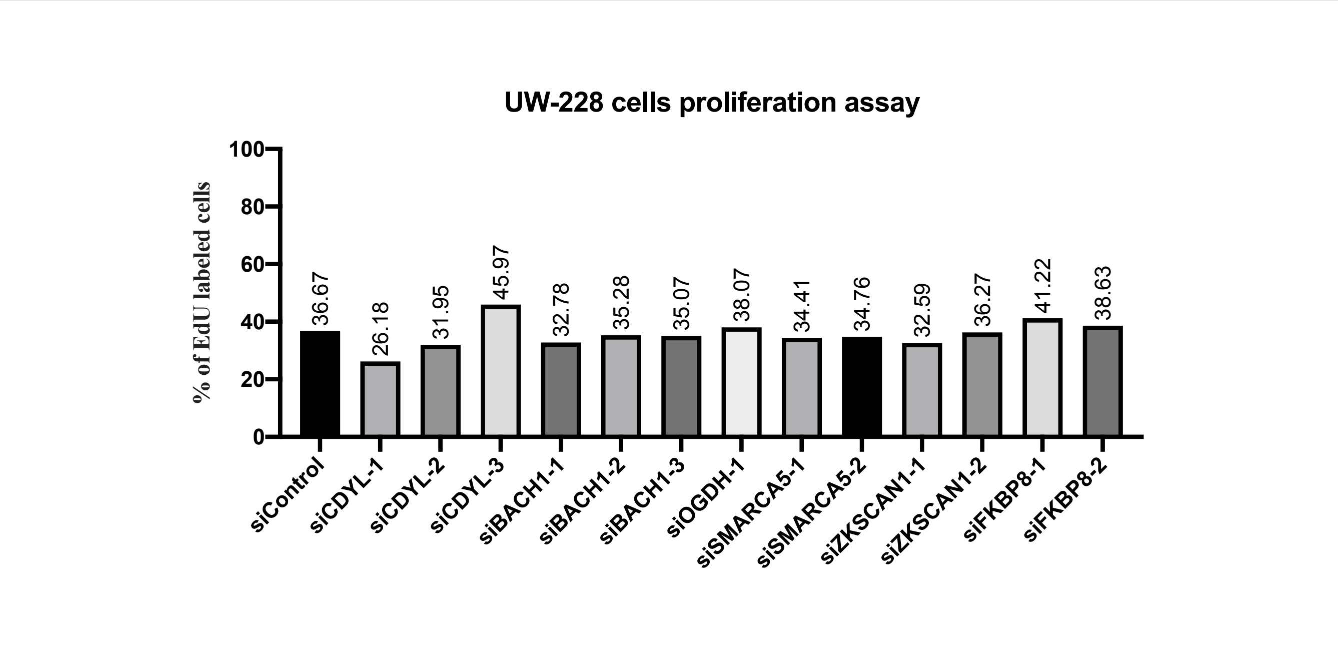
UW-228 cells proliferation assay
% of EdU labeled cells
100
80
60
40
20
0
36.67
26.18
31.95
45.97
32.78
35.28
35.07
38.07
34.41
34.76
32.59
36.27
41.22
38.63
siControl
siCDYL-1
siCDYL-2
siCDYL-3
siBACH1-1
siBACH1-2
siBACH1-3
siOGDH-1
siSMARCA5-1
siSMARCA5-2
siZKSCAN1-1
siZKSCAN1-2
siFKBP8-1
siFKBP8-2

Figure S6

F

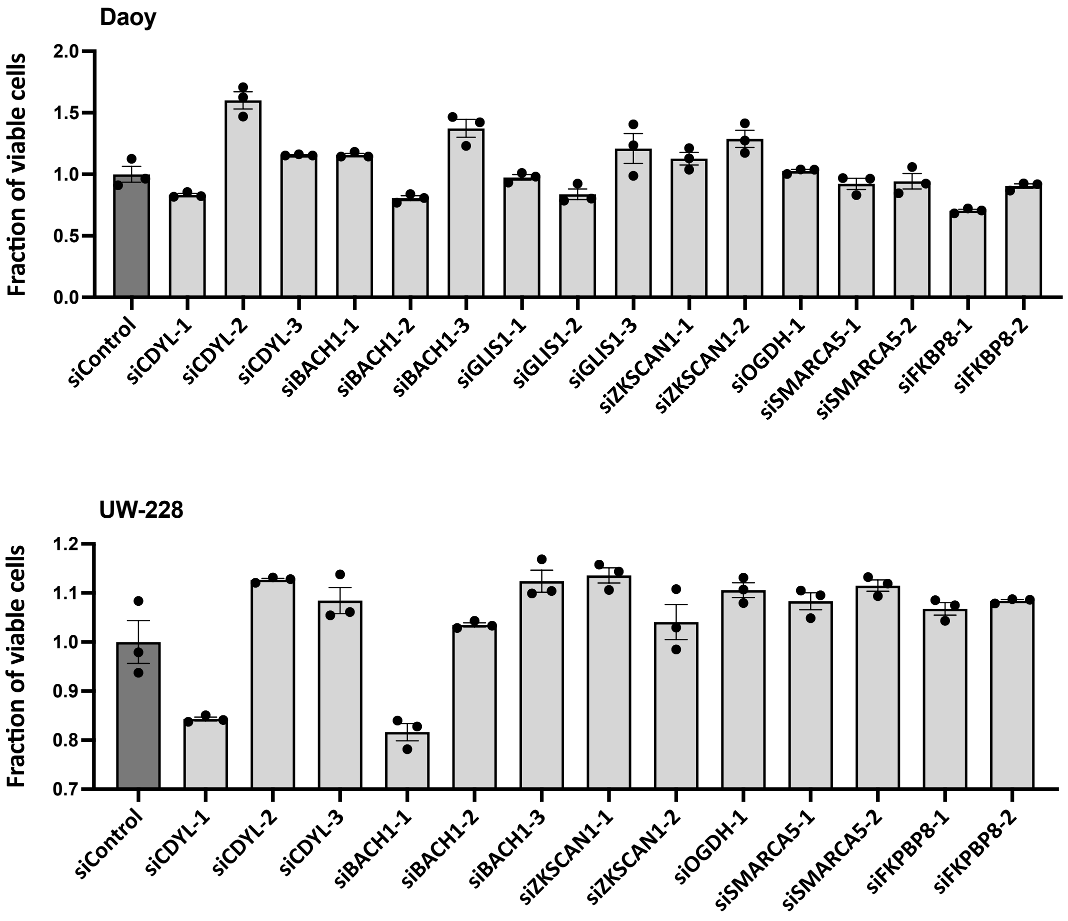
Daoy
Fraction of viable cells
2.0
1.5
1.0
0.5
0.0
siControl
siCDYL-1
siCDYL-2
siCDYL-3
siBACH1-1
siBACH1-2
siBACH1-3
siGLIS1-1
siGLIS1-2
siGLIS1-3
siZKSCAN1-1
siZKSCAN1-2
siOGDH-1
siSMARCA5-1
siSMARCA5-2
siFKBP8-1
siFKBP8-2
UW-228
Fraction of viable cells
1.2
1.1
1.0
0.9
0.8
0.7
siControl
siCDYL-1
siCDYL-2
siCDYL-3
siBACH1-1
siBACH1-2
siBACH1-3
siZKSCAN1-1
siZKSCAN1-2
siOGDH-1
siSMARCA5-1
siSMARCA5-2
siFKPBP8-1
siFKPBP8-2

Figure S6

G

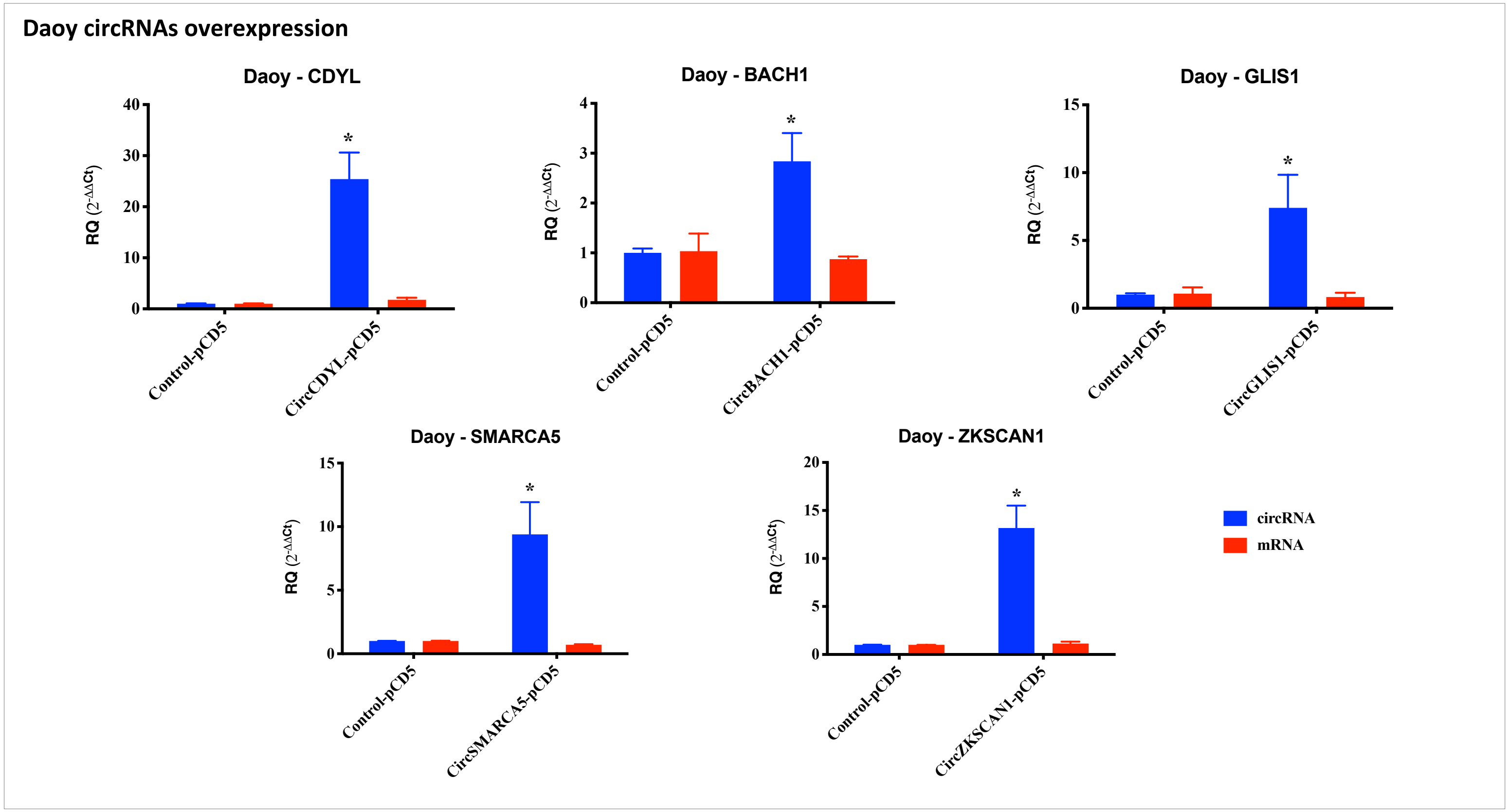
Daoy circRNAs overexpression
Daoy - CDYL
Daoy - BACH1
Daoy - GLIS1
Daoy - SMARCA5
Daoy - ZKSCAN1
RQ ($2^{-\Delta\Delta Ct}$)
Control-pCD5
CircCDYL-pCD5
CircBACH1-pCD5
CircGLIS1-pCD5
CircSMARCA5-pCD5
CircZKSCAN1-pCD5
circRNA
mRNA

Figure S6

H

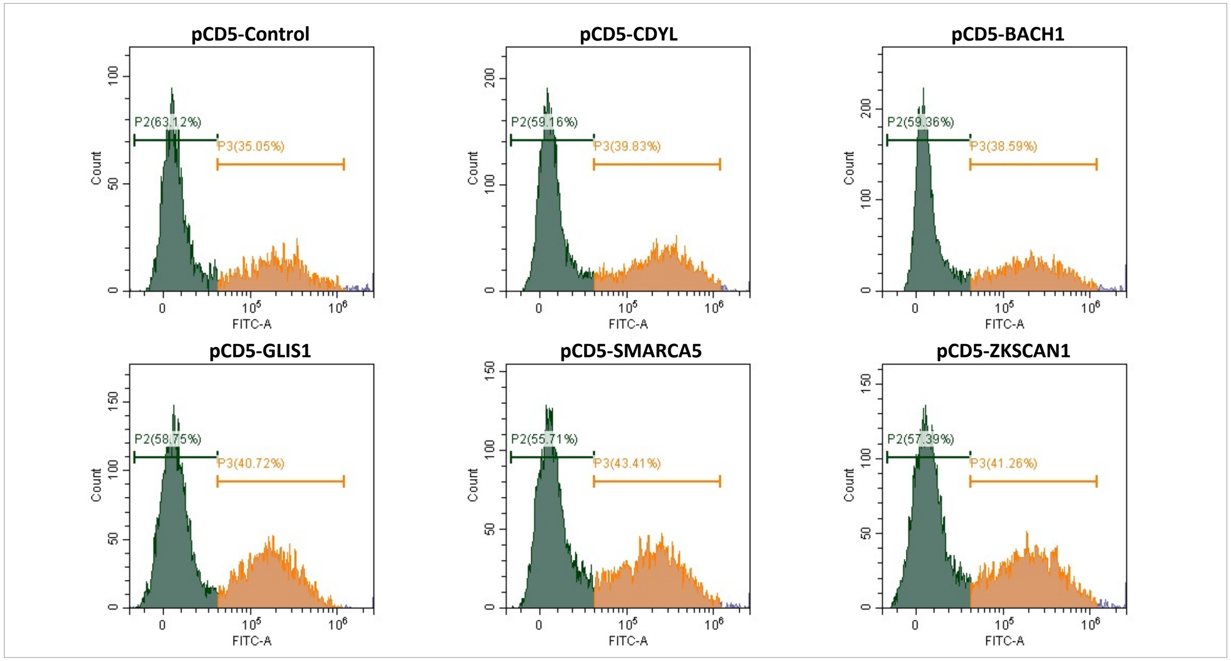
pCD5-Control
P2(63.12%)
P3(35.05%)
pCD5-CDYL
P2(59.16%)
P3(39.83%)
pCD5-BACH1
P2(59.36%)
P3(38.59%)
pCD5-GLIS1
P2(58.75%)
P3(40.72%)
pCD5-SMARCA5
P2(55.71%)
P3(43.41%)
pCD5-ZKSCAN1
P2(57.39%)
P3(41.26%)
Count
FITC-A

I

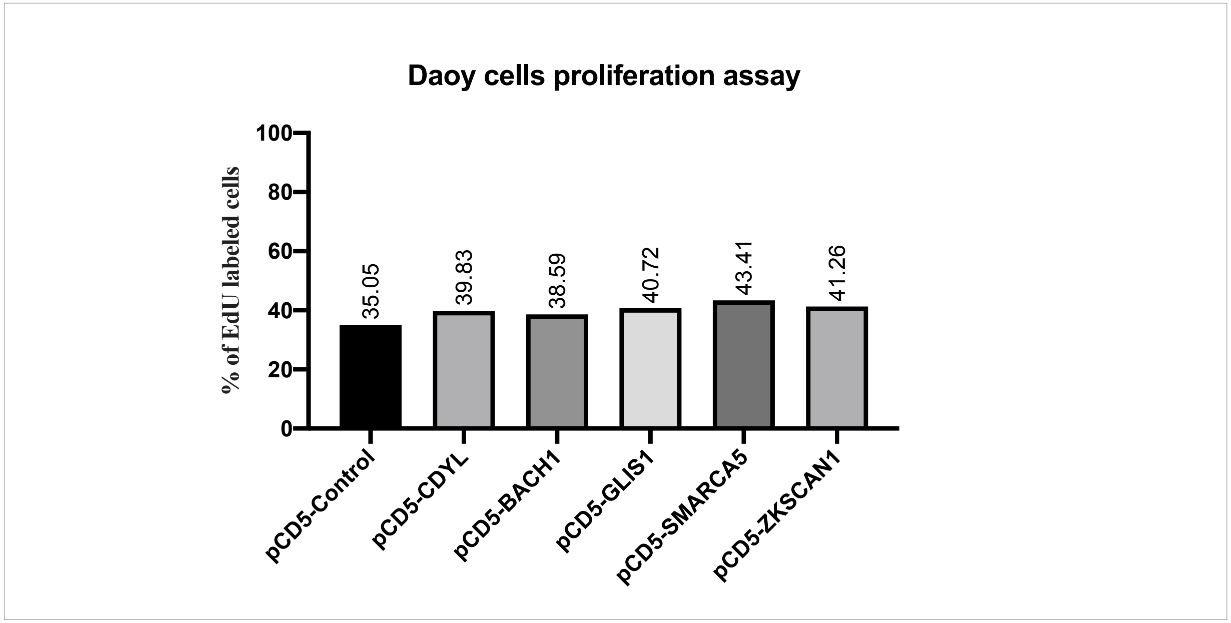
Daoy cells proliferation assay
% of EdU labeled cells
35.05
39.83
38.59
40.72
43.41
41.26
pCD5-Control
pCD5-CDYL
pCD5-BACH1
pCD5-GLIS1
pCD5-SMARCA5
pCD5-ZKSCAN1

Supplementary Figure 6. Effects of depletion or overexpression of selected circRNAs on cell proliferation in medulloblastoma cell lines. (**A**) Knock-down efficiency of the 7 selected circRNAs by siRNAs targeting the back-spliced junction in Daoy and UW-228 cells, determined by qPCR. The relative expression ($2^{-\Delta\Delta Ct}$ values) of circRNAs and mRNAs, normalized to the housekeeping gene (TBP) and the control siRNA treatment is shown in blue and red, respectively. Error bars indicate the SEM of three independent technical replicates. RQ denotes the relative quantification of the circRNA and mRNA expression. ANOVA test was applied to calculate statistically significant differences ($^{*,\#}$: $p < 0.05$, $^{**,\#\#}$: $p<0.01$) between each treatment versus the control. EdU incorporation assays of Daoy (**B**) and UW-228 cells (**C**) following 72 hr siRNA transfection. A representative experiment is shown, with the percentage of EdU labeled Daoy and UW-228 cells also summarized in (**D**) and (**E**), respectively. (**F**) Daoy and UW-228 cells were transfected with siRNAs for 72 hr, and the fraction of metabolically active cells normalized to the control siRNA treatment was determined by the WST-1 assay. Error bars indicate the SEM of three technical replicates. In all cases at least 2 different siRNAs targeting the back-spliced junction of a circRNA were used except for OGDH where the second siRNA is ineffective, as seen in (A). (**G**) Overexpression efficiency of the 5 circRNA expression constructs in Daoy cells. The qPCR analysis was performed as in (A). (**H, I**) EdU incorporation assays of Daoy cells following 48 hr transfection with circRNA expression constructs. The analysis was performed as in (B) and (D).
